# HAP40 functions as a proteostasis regulator by controlling huntingtin interactions and its release into the extracellular space

**DOI:** 10.1073/pnas.2611301123

**Published:** 2026-10-22

**Authors:** Eduardo Silva Ramos, Annett Boeddrich, Christian Haenig, Philipp Trepte, Orchid Ammar, Christopher Secker, Oliver Popp, Andranik Ivanov, Stephanie Beetz, Leonard Roth, Roxane M. Papawassiliou, Luisanna C. Hernandez Jeppesen, Theo Wallenfang, Brandon Keith, Rachel J. Harding, Franziska Schindler, Tomas Koudelka, Nadine Scharek, Martina Zenkner, Nancy Neuendorf, Sabrina Golusik, Philipp Mertins, Ilaria Piazza, Sigrid Schnoegl, Erich E. Wanker

**Affiliations:** ^a^ Neuroproteomics, Max-Delbrück-Center for Molecular Medicine in the Helmholtz Association, Berlin 13125, Germany; ^b^ Distributed Algorithms and Supercomputing, Zuse Institute Berlin, Berlin 14195, Germany; ^c^ Proteomics Platform, Max-Delbrück-Center for Molecular Medicine in the Helmholtz Association, Berlin 13125, Germany; ^d^Core Unit Bioinformatics, Berlin Institute of Health at Charité, University Medicine Berlin, Berlin 10117, Germany; ^e^ Structural Genomics Consortium, University of Toronto, Toronto, ON M5G1L7, Canada; ^f^ Department of Pharmacology and Toxicology, University of Toronto, Toronto, ON M5G1L7, Canada; ^g^ Leslie Dan Faculty of Pharmacy, University of Toronto, Toronto, ON M5S3M2, Canada; ^h^ Princess Margaret Cancer Centre, University Health Network, Toronto, ON M5G 2M9, Canada; ^i^ Allosteric Proteomics, Max-Delbrück-Center for Molecular Medicine in the Helmholtz Association, Berlin 13125, Germany

**Keywords:** Huntington’s disease, mutant HTT, HAP40, proteostasis, autophagy

## Abstract

Huntingtin-associated protein 40 (HAP40) forms a stable protein complex with huntingtin (HTT). Its cellular function and how HAP40 loss influences mutant HTT (mHTT) abundance and pathobiology are currently unclear. Here, using diverse cellular models and omics methods, we demonstrate that HAP40 is an obligate interaction partner of HTT and through its binding controls HTT interactions and the abundance of HTT-associated proteins, indicating that it functions as a HTT interaction regulatory unit. Also, loss of HAP40 in mHTT-expressing striatal cells impairs autophagic protein degradation and triggers transcriptional activation of the CLEAR network, demonstrating that it functions as a proteostasis regulator that acts on quality control pathways. Finally, mHTT-expressing cells lacking HAP40 showed increased secretion of a ~205 kDa mHTT fragment through an endoplasmic reticulum (ER)/Golgi-associated route, indicating that striatal cells reduce intracellular mHTT-induced proteotoxicity through activation of secretory pathways. Together, these results establish HAP40 as a critical proteostasis regulator that through controlling HTT interactions maintains cellular homeostasis.

## Significance

Huntingtin (HTT) interacts with thousands of proteins, including Huntingtin-associated protein 40 (HAP40), which stabilizes its conformation. How HAP40 influences HTT abundance and function is not fully defined. Here, using HAP40 knockout models, we report that HAP40 is an obligate binding partner which fundamentally controls HTT behavior. HAP40 not only alters HTT conformation but also defines its subcellular localization and protein interaction profile. Loss of HAP40 impairs autophagic protein degradation, triggers massive transcriptional dysregulation and leads to compensatory secretion of a large ~205 kDa mutant HTT fragment into the extracellular space through Golgi-associated pathways. These findings establish HAP40 as a proteostasis regulator that is required to stabilize canonical HTT interactions and mitigate mutant HTT proteotoxicity.

Huntington’s disease (HD) is a monogenic, neurodegenerative disorder characterized by a triad of motor, cognitive, and psychiatric symptoms (1, 2). It is caused by a CAG repeat expansion located in exon 1 of the *huntingtin* (*HTT*) gene, leading to the production of HTT protein with an elongated polyglutamine (polyQ) tract of largely unknown molecular function (1, 3). PolyQ-expanded HTT is believed to cause neurotoxicity through a dominant gain-of-function mechanism, potentially disrupting a wide range of cellular processes, including autophagy (4), transcription (5), splicing (6), and synaptic transmission (7). The pathogenic polyQ tract may also alter HTT’s normal function in medium spiny neurons, suggesting that both loss- and gain-of-function mechanisms may drive HD pathology. How the pathogenic polyQ tract in HTT causes dysfunction and neurodegeneration in HD brains is currently not well understood (8).

HTT is a large ~350 kDa protein that is widely expressed in different cell-types and tissues (3). In mice, HTT was shown to be essential for early embryogenesis (9), while its ablation in neurons of adult animals was nondeleterious (10). HTT is largely composed of α-helical “HEAT” repeats that form folded superhelical structures (11) and are thought to function as protein–protein interaction (PPI) modules (12). In line with this, thousands of HTT-associated proteins have been identified in interaction screens with full-length HTT or its fragments (13–15), suggesting that HTT functions as an interaction hub, which binds diverse partner proteins (3). However, despite our knowledge about HTT PPIs, it remains unclear through which domains HTT interacts with proteins and how full-length HTT controls the function of associated cellular proteins. Also, it is unknown how interaction partners specifically influence HTT abundance. Such knowledge is crucial for the development of HTT lowering strategies, because proteins that regulate mHTT abundance may be interesting targets for therapeutic development.

The ~40 kDa HTT-associated protein HAP40 stands out among the ~3,000 reported human HTT interaction partners (14, 16). It binds to full-length HTT in a cleft and stabilizes its secondary structure (16). Through the association of HAP40, a compact heterodimeric HTT–HAP40 complex is formed that likely interacts with other proteins and thereby may regulate cellular activities. Recent structural and functional investigations with recombinant proteins, cell models, and *Drosophila melanogaster* support the hypothesis that HTT–HAP40 heterodimers are functionally relevant (17, 18). This is also supported by overexpression and gene knock-down experiments, indicating that HTT overproduction as well as depletion influence HAP40 protein levels (19, 20).

Currently, the cellular function of HAP40 is unknown. Also, it is unclear how HAP40 influences mHTT abundance and pathobiology. Experimental evidence indicates that HTT interacts with the cargo receptor p62 and functions as a scaffold for selective autophagy (21, 22), suggesting that HAP40 binding to HTT might influence this degradation process. Furthermore, it was shown that HTT associates with the Golgi apparatus (23, 24) and facilitates vesicle trafficking (25), suggesting that HAP40 may also influence this subcellular process. An association of HAP40 with the GTPase Rab5, which regulates vesicle fusion at endosomes and lysosomes (26), has also been described (27). Whether inactivation of HAP40 influences specific protein degradation pathways or vesicle transport processes, however, remains unclear.

In this study, we assessed the cellular function of HAP40, utilizing multiple HAP40 knock-out (KO) cell lines, omics methods as well as a set of biochemical and functional assays. Also, we addressed the question of how HAP40 controls mHTT abundance and pathobiology in cells. We found that HAP40 is an obligate HTT interaction partner and controls its association with other cellular proteins, indicating that it functions as an interaction regulatory unit that indirectly controls the abundance of HTT-associated proteins. Also, we observed that depletion of HAP40 in mHTT-expressing mouse striatal cells causes transcriptional dysregulation, activation of the CLEAR (Coordinated Lysosomal Expression and Regulation) pathway and impairment of autophagy, indicating that HAP40 functions as a proteostasis regulator, which controls the activity of protein degradation pathways. Finally, we found that loss of HAP40 increases the secretion of mHTT into the extracellular space, suggesting that cells reduce intracellular mHTT abundance and proteotoxicity through activation of secretory pathways. The potential implications of our findings for the development of causal therapies for HD treatment are discussed.

## Results

### Loss of HAP40 Decreases the Abundance of Full-Length HTT in HEK293 and Striatal Cells.

**Fig. 1.**
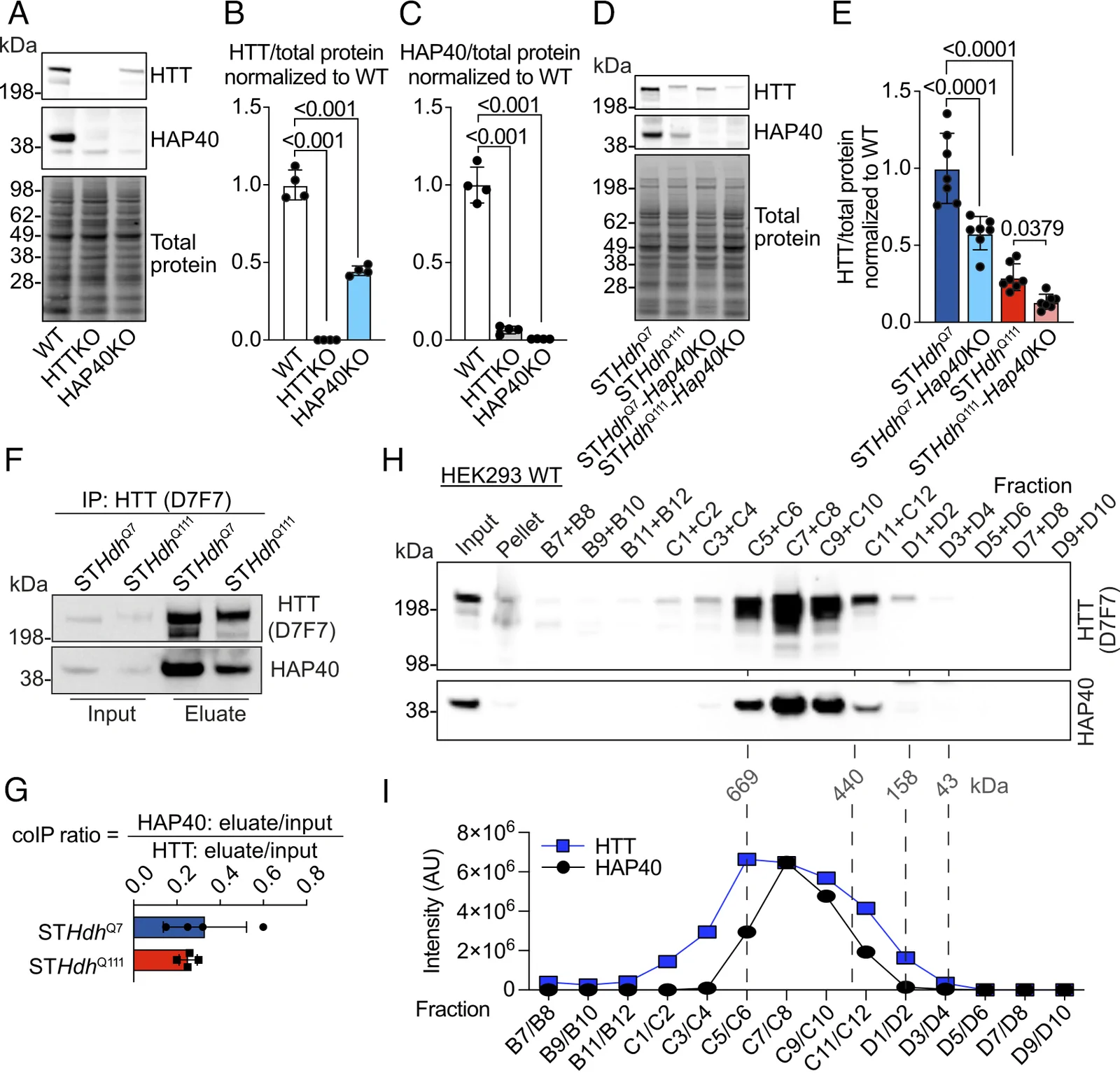
HTT and HAP40 form a stable complex. (*A*) Representative immunoblot of HEK293 total cell lysates. Membranes were probed with antibodies against HTT (D7F7) and HAP40. Quantification of (*B*) HTT and (*C*) HAP40 protein levels in *A*, expressed relative to total protein and normalized to HEK WT. Data are presented as mean ± SD of four biological replicates. Statistical significance was determined via one-way ANOVA followed by Dunnett’s multiple comparisons test. (*D*) Representative immunoblot of mouse striatal total cell lysates. Membranes were probed with antibodies against HTT (D7F7) and HAP40. For (*A* and *D*) total protein was visualized using No-Stain protein labeling reagent, which served as a loading control. (*E*) Quantification of HTT protein levels in *D*, expressed relative to total protein and normalized to ST*Hdh*^Q7^. Data are presented as mean ± SD of seven biological replicates. Statistical significance was determined using one-way ANOVA with Holm–Šídák correction for multiple comparisons. (*F*) Representative immunoblots of co-IPs of HTT with anti-HTT (D7F7) antibody using cultured mouse striatal cell lysates. IP fractions represent 5% input sample and 100% eluate. Samples were subjected to SDS-PAGE and immunoblotted for HTT using anti-HTT (D7F7) and anti-HAP40 antibodies. (*G*) Quantification of the co-IP enrichment ratio of HAP40 relative to HTT from co-IPs shown in *F*. Eluate band intensities for HAP40 and HTT were normalized to their respective input band intensities. The enrichment value of HAP40 was then normalized to that of HTT. Data is presented as mean ± SD of three biological replicates. No significant difference (ns) was observed (Student’s *t* test). (*H*) Representative SEC of HEK293 cell total lysates. Fractions were pooled yielding 14 final fractions. Acetone-precipitated proteins were subjected to SDS-PAGE and immunoblotting using anti-HTT (D7F7) and anti-HAP40 antibodies. From input and pellet samples 10 µg were loaded, while 10% of two pooled fractions was loaded. Molecular weight markers are displayed below the HAP40 immunoblot. Two independent experiments were performed. (*I*) HTT and HAP40 immunoblot intensities in fractions B7 to D10 of SEC from *H*. On top of the diagram molecular weight standards are shown which were used for calibration of the SEC column. Band intensities are shown as an arbitrary value (AU) and plotted against the pooled fractions.

Previous investigations suggest that HAP40 and HTT protein levels in cells are interdependent (17). To further explore this relationship, we used CRISPR/Cas9 genome editing to generate a HEK293 HAP40 knockout (HEK293-HAP40KO) cell line (*SI Appendix*, Fig. S1*A*). We found that HTT^Q23^ (also termed “apo-HTT” i.e., HTT not bound by HAP40) levels are ~50% lower in HEK293-HAP40KO cells than in wild-type (WT) cells (Fig. 1 *A* and *B*). In comparison, the depletion of HTT^Q23^ in HEK293-HTTKO cells caused an almost complete loss of HAP40 (Fig. 1 *A* and *C*), confirming previous reports (17). The levels of both HAP40 and HTT^Q23^ were partially rescued when the respective proteins were overproduced in HAP40KO or HTTKO cell lines (*SI Appendix*, Fig. S1 *B* and *C*).

To assess whether these effects extend to mouse striatal cells and pathogenic HTT alleles, we used homozygous cell lines (28) that endogenously express either nonpathogenic (ST*Hdh*^Q7/Q7^; here on ST*Hdh*^Q7^) or pathogenic (ST*Hdh*^Q111/Q111^; here on ST*Hdh*^Q111^) HTT. We generated *Hap40*KO derivatives of both lines using CRISPR/Cas9 (*SI Appendix*, Fig. S1 *D* and *E*). In the unmodified parental lines, we found that steady-state HTT^Q111^ protein levels are ~50% lower than HTT^Q7^ levels (Fig. 1 *D* and *E*), in line with previous observations (29, 30). Knock-out of HAP40 further reduced HTT levels in both ST*Hdh*^Q111^-*Hap40*KO and ST*Hdh*^Q7^-*Hap40*KO cell lines (Fig. 1 *D* and *E*). A similar result was obtained when HTT^Q111^ protein levels were quantified using the monoclonal anti-HTT antibody MAB2166 (*SI Appendix*, Fig. S1 *F* and *G*).

To assess whether transcriptional dysregulation is responsible for the HTT protein abundance changes, we quantified HTT transcript levels utilizing a q-PCR assay (31). This analysis revealed that both *Htt*^Q111^ and *Htt*^Q7^ transcripts were mildly decreased in striatal *Hap40*KO cells compared to controls (*SI Appendix*, Fig. S1*H*), suggesting that transcriptional changes do not account for the observed ~50% reductions in protein levels.

### HAP40 Is an Obligate Interaction Partner of HTT.

The association of HAP40 and HTT in cells is currently not well characterized. To address this, we first performed coimmunoprecipitations (co-IPs) to enrich the HTT–HAP40 complex from protein extracts of striatal cells. Anti-HTT (D7F7) antibody was utilized for co-IPs, because it recognizes an epitope of HTT not required for HAP40 binding (*SI Appendix*, Fig. S1*I*). We observed that HAP40 amounts enriched from striatal ST*Hdh*^Q7^ and ST*Hdh*^Q111^ cell extracts are proportional to HTT protein levels (Fig. 1 *F* and *G*), indicating that both HTT^Q7^ and HTT^Q111^ bind similar amounts of HAP40. Enrichment of stable HTT–HAP40 complexes was also observed when crude brain extracts of zQ175 HD knock-in (32) and control mice were investigated (*SI Appendix*, Fig. S1*J*).

Next, to investigate colocalization of HTT and HAP40, we generated a HEK293 knock-in cell line expressing HTT^Q23^ fused at its C-terminus with mNeonGreen (HTT^Q23^-mNG) (*SI Appendix*, Fig. S1*K*). Immunolabeling of HTT^Q23^-mNG producing cells with an anti-HAP40 antibody revealed a high degree of HTT^Q23^-mNG and HAP40 colocalization in distinct foci in the perinuclear region of HEK293 cells (*SI Appendix*, Fig. S2 *A* and *B*), supporting the hypothesis that both proteins form a complex under physiological conditions. Very similar results were obtained when WT HEK293 cells and HTT^Q23^-mNG-producing cells were immunolabeled with anti-HTT and anti-HAP40 antibodies (*SI Appendix*, Fig. S2 *C*–*J*). The specificities of the applied antibodies for their respective targets were confirmed with HEK293-HTTKO and -HAP40KO cell lines (*SI Appendix*, Fig. S2 *K* and *L*). Next, the localization of HTT^Q23^ was examined in HEK293-HAP40KO cells using a validated anti-HTT (MAB2166) antibody. In contrast to the WT cells, HEK293-HAP40KO cells displayed a weaker perinuclear HTT^Q23^ localization and a more dispersed cellular distribution (*SI Appendix*, Fig. S2 *M* and *N*). Surprisingly, the corrected total cell fluorescence quantified with MAB2166 antibody was higher in HEK293-HAP40KO than in WT cells (*SI Appendix*, Fig. S2*O*), suggesting that antibody binding to apo-HTT with a native conformation is somehow stronger than to HTT in the HAP40 complex. However, such a difference in antibody binding was not observed after protein denaturation by SDS-PAGE (Fig. 1*A*).

Next, to rule out that the perinuclear HTT foci observed by immunofluorescence microscopy (IFM) represent aggresomes (33), we performed filter retardation assays and found no higher molecular weight HTT aggregates in HTT^Q23^-mNG-producing HEK293 cells (*SI Appendix*, Fig. S3*A*). In contrast, such structures were detectable when short HTT exon-1 fragments with pathogenic and nonpathogenic polyQ tracts (HTTexon1Q48- and HTTexon1Q23-EYFP) were overproduced in HTT^Q23^-mNG HEK293 cells. Furthermore, coimmunostaining for HDAC6 and vimentin, two proteins known to colocalize with aggresomes (33, 34), showed no colocalization with the perinuclear HTT^Q23^-mNG foci (*SI Appendix*, Fig. S3 *B* and *C*). Instead, coimmunostaining with an antibody for the Golgi protein GOLGA2 revealed significant colocalization with HTT^Q23^-mNG (*SI Appendix*, Fig. S3 *D* and *E*), suggesting that HTT^Q23^-mNG prominently associates with perinuclear Golgi structures in HEK293 cells. This is also supported by IFM with striatal cells (*SI Appendix*, Fig. S3 *F* and *G*) and biochemical studies, indicating that both HTT and HAP40 are enriched in Golgi membrane fractions (*SI Appendix*, Fig. S4), while control proteins such as GAPDH or LAMP1 are not.

Finally, we performed size-exclusion chromatography (SEC) experiments using HEK293 cells to determine the size distribution of endogenously formed HTT–HAP40 heterodimers. Analysis by SDS-PAGE and immunoblotting revealed molecular weights between ~440 and ~670 kDa for size-fractionated HTT^Q23^ and HAP40 (Fig. 1 *H* and *I* and *SI Appendix*, Fig. S5 *A* and *B*). This suggests that additional proteins may be bound to HTT–HAP40 heterodimers, which in vitro exhibit a molecular weight of ~390 kDa (16). An analysis of mouse brain extracts with SEC yielded similar cofractionation profiles (*SI Appendix*, Fig. S5*C*). Interestingly, overlapping elution profiles of HAP40 and HTT^Q23^ were obtained (Fig. 1*I*), suggesting that most if not all HAP40 protein is stably bound to HTT^Q23^ in HEK293 cells. HTT^Q23^, but not HAP40, was detectable in high molecular weight protein fractions (>669 kDa), suggesting that not all HTT^Q23^ molecules in cells are bound by HAP40. We explored this notion further by performing SEC experiments with HEK293-HAP40KO cells and found that these cells compared to WT cells exhibited an even higher amount of high molecular weight HTT^Q23^ (*SI Appendix*, Fig. S5 *D*–*F*). Thus, apo-HTT in the absence of HAP40 likely forms large abnormal protein complexes in cells.

### HAP40 Binding Alters the Conformation of Full-Length HTT^Q23^.

We hypothesized that loss of HAP40 may alter the structure of HTT and perturb its protein-binding properties. To address this, we first investigated the interaction between HTT^Q23^ and HAP40 utilizing a quantitative BRET assay (35). With this method the interactions between nanoluciferase (NL, donor)- and ProteinA-mCitrine (PA-mCit, acceptor)-tagged fusion proteins can be detected with high specificity and sensitivity in cells, when the sensor proteins NL and PA-mCit are in close proximity (<10 nm). The available cryo-EM structures of HTT–HAP40 heterodimers (16, 19) predict that N and C termini of HTT and HAP40 are in close proximity (*SI Appendix*, Fig. S6*A*), suggesting that N-N and C-C tagging configurations with sensor proteins might reveal BRET, while N-C and C-N tagging might not, because the sensor proteins are far apart. Strikingly, our measurements revealed strong BRET signals, when N-N (NL-HTT^Q23^ vs. PA-mCit-HAP40) or C-C (HTT^Q23^-NL vs. HAP40-mCit-PA) tagged reporter protein fusions were coproduced in HEK293 WT cells (*SI Appendix*, Fig. S6 *B* and *C*). In contrast, BRET signals were undetectable with the N-C (NL-HTT^Q23^ vs. HAP40-mCit-PA) or C-N (HTT^Q23^-NL vs. PA-mCit-HAP40) tagging configurations, confirming our structure-based predictions. Next, we assessed whether BRET measurements can detect the impact of amino acid changes on HTT–HAP40 interactions. Informed by the structure of the HTT–HAP40 complex, we focused on five negatively charged residues in HAP40 (E316, E317, E331, D333, and E335), which were predicted to be critical for complex stability (16). We generated three PA-mCit-tagged HAP40 protein variants in which two (E316K, E317K; M2), three (E331K, D333K, and E335K; M3), or five (E316K, E317K, E331K, D333K, and E335K; M5) negatively charged residues were exchanged for positive lysine residues (*SI Appendix*, Fig. S6*D*). Then, each of these proteins was coproduced together with NL-HTT^Q23^ in HEK293 cells and BRET ratios were quantified. In comparison to PA-mCit-HAP40^WT^ the protein variants PA-mCit-HAP40^M2, M3, and M5^ showed significantly reduced BRET values (*SI Appendix*, Fig. S6*E*), indicating reduced binding of mutated HAP40 protein variants to HTT.

Next, to investigate the impact of amino acid exchanges on the stability of HTT–HAP40 heterodimers, we coproduced each protein variant together with HTT^Q23^-FLAG and copurified protein complexes with FLAG-affinity and SEC (19). We observed protein complexes with HAP40^WT^ and all investigated protein variants (HAP40^M2, M3, and M5^). Compared to HAP40^WT, M2, and M3^, however, coenrichment of HAP40^M5^ was low (*SI Appendix*, Fig. S6*F*), confirming that this M5 protein variant binds weakly to HTT^Q23^. This result is also supported by differential scanning fluorimetry experiments (*SI Appendix*, Fig. S6*G*).

**Fig. 2.**
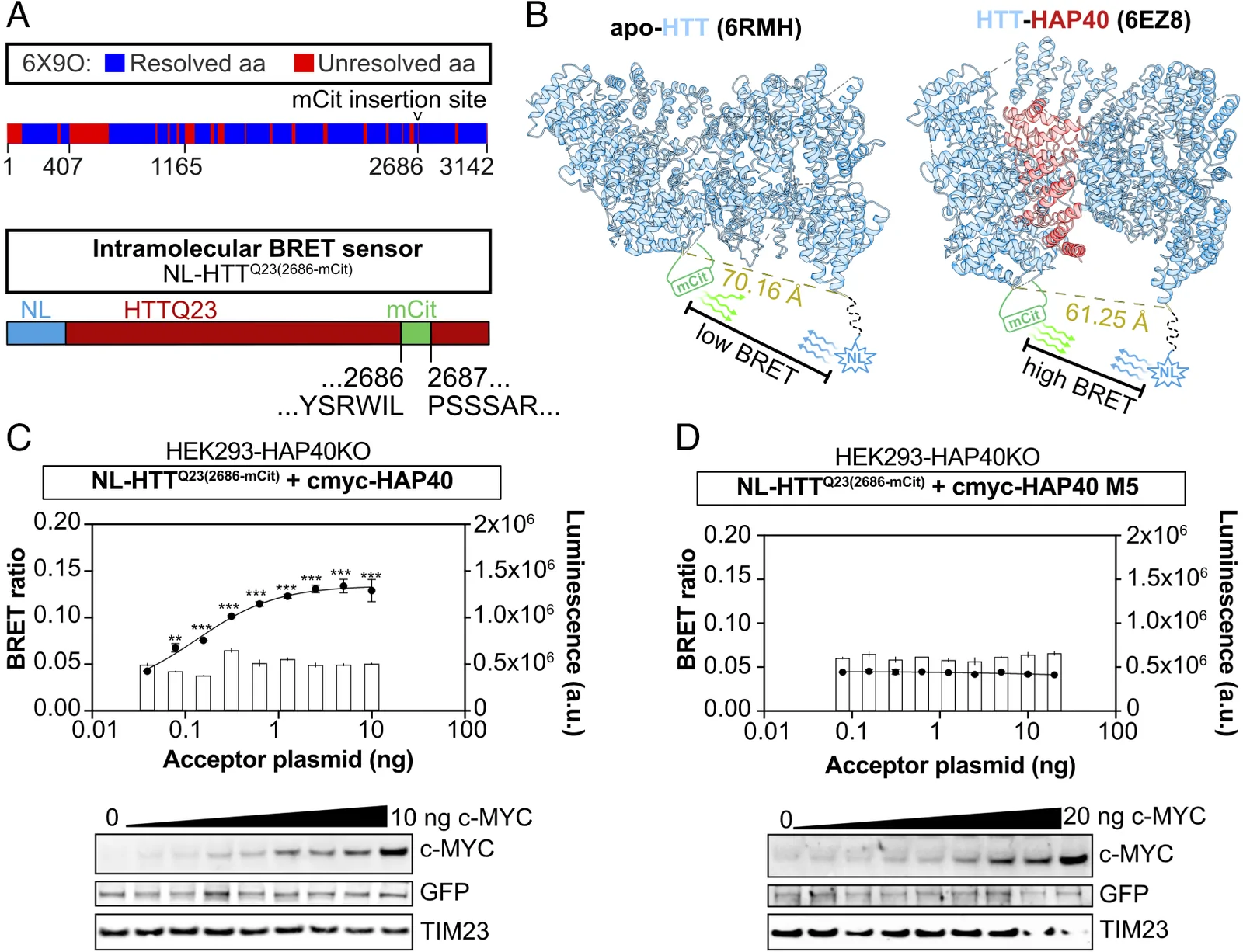
HAP40 binding alters the conformation of the HTT–HAP40 complex. (*A*) Intramolecular HTT BRET sensor. *Top*: linear schematic representation of resolved (blue) and unresolved (red) segments of the cryo-EM structure of HTT–HAP40 (6X9O), indicating the mCitrine (mCit) insertion site. Below: schematic representation of the HTT intramolecular BRET sensor (NL-HTT^Q23(2686-mCit)^), displaying the tagging positions of NanoLuc at the N terminus and mCitrine between amino acids 2686 and 2687 of human HTT. (*B*) Cryo-EM structures of apo-HTT (6RMH) and HTT–HAP40 (6EZ8), illustrating the distances between resolved residues near the mCitrine insertion site and the N terminus of HTT. Distances are shown in angstroms (Å). The relative positions of NanoLuc and mCitrine are indicated, along with predicted differences in BRET signal based on donor-acceptor distance. (*C*) BRET saturation assay with cotransfection of the HTT intramolecular BRET sensor (150 ng, pcDNA-NL-HTT^Q23(2686-mCit)^) and increasing amounts of pcDNA-c-myc-HAP40 (wild-type; 0 to 10 ng) in HEK293-HAP40KO cells. The plot displays BRET ratio values (dashed line) and luminescence values as a proxy for HTT levels of the BRET sensor (bar graph). Data are presented as mean ± SD (n = 2). Statistical significance was determined via one-way ANOVA followed by Dunnett’s multiple-comparisons test. Corresponding immunoblots related to BRET assay using anti-c-myc, anti-GFP, and anti-TIM23 (loading control) antibodies are shown below. (*D*) BRET saturation assay using the HTT intramolecular BRET sensor with increasing amounts of c-myc-HAP40 (5 M mutant). Experimental conditions and statistical analysis were performed as described in *C*.

Based on the reported cryo-EM structures (16, 19, 36), we hypothesized that full-length HTT^Q23^ in the presence and absence of HAP40 in cells might be structurally distinct. To test this in live cells, we engineered the intramolecular BRET sensor NL-HTT^Q23(2686-mCit)^ (Fig. 2*A*). In this protein NL is fused to the N terminus of HTT and mCitrine is incorporated into an unstructured loop at leucine 2686. We reasoned that HAP40 binding might induce a conformational compaction of HTT^Q23^, bringing the N- and C-terminal regions into closer proximity and thereby increasing BRET (Fig. 2*B*). Consistent with this, coproduction of NL-HTT^Q23(2686-mCit)^ together with HAP40^WT^ in HEK293-HAP40KO cells revealed a significant BRET increase (Fig. 2*C*), while such an effect was not observed with the HAP40^M5^ protein variant (Fig. 2*D*). This indicates that the molecular distance between the N terminus and the unstructured C-terminal loop region (L2686) is significantly larger in apo-HTT in the absence of HAP40, confirming previously reported structural models (19). Importantly, SDS-PAGE and immunoblotting confirmed that both HAP40^WT^ and HAP40^M5^ are expressed at comparable levels (Fig. 2 *C* and *D*), ruling out expression differences as a confounding factor.

### Loss of HAP40 Alters HTT’s Interaction Profile.

**Fig. 3.**
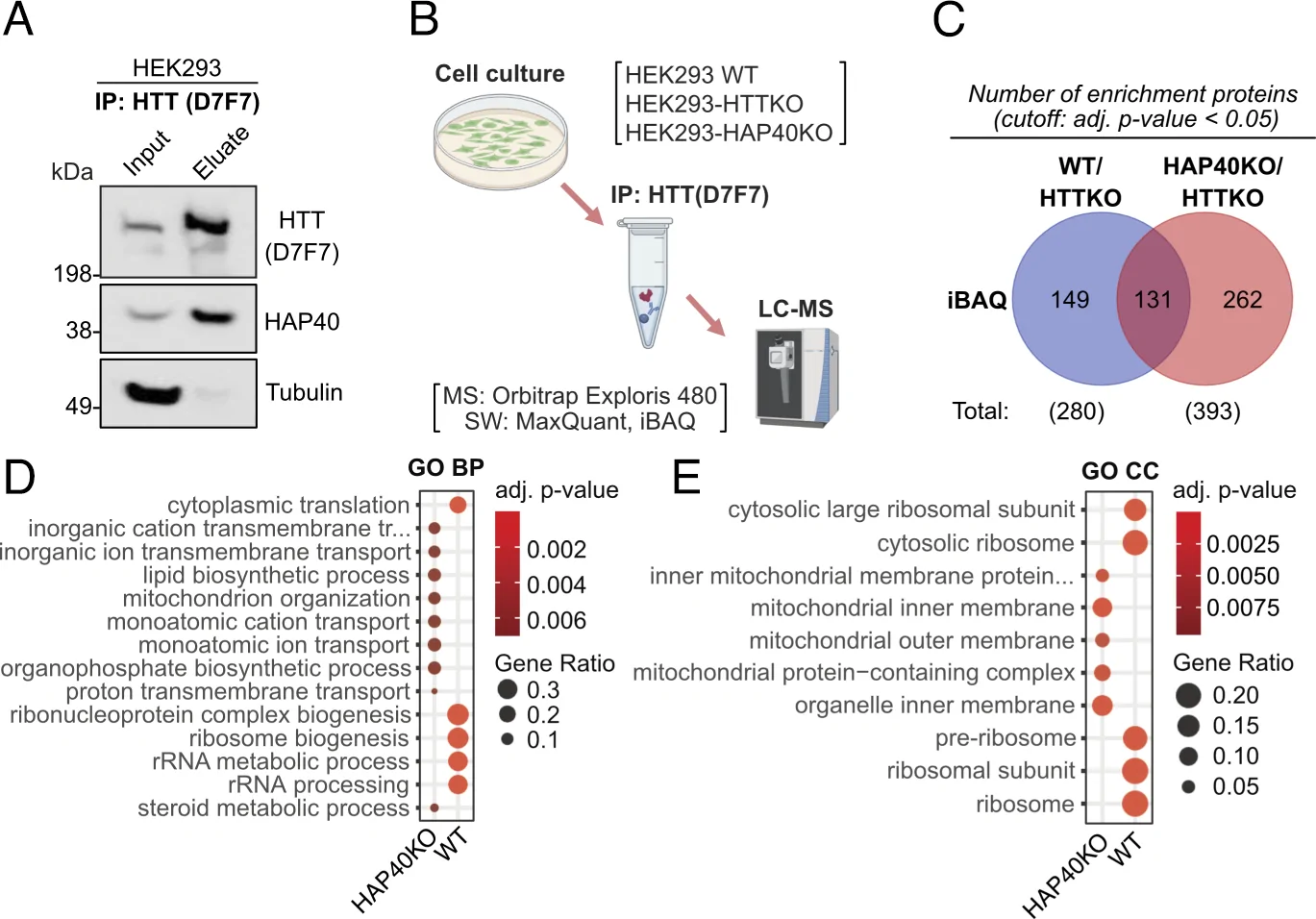
HTT interactome changes in the absence of HAP40. (*A*) Immunoblots of HTT co-IPs from WT HEK293 lysates using anti-HTT (D7F7) antibody. Lanes display 5% input and 80% eluate relative to starting material. Samples were analyzed by SDS-PAGE and probed for HTT and HAP40. Data are representative of four independent experiments. (*B*) Schematic of the HTT co-IP-MS workflow. Three HEK293 cell lines (WT, HTTKO, and HAP40KO) were subjected to co-IP with anti-HTT (D7F7) followed by LC-MS/MS analysis. Four biological replicates of each cell line were used. (*C*) Venn diagram of proteins significantly enriched (adj. *P*-value < 0.05) by co-IP in the two experimental contexts: i) HTT–HAP40 complexes isolated from WT cells and ii) apo-HTT complexes isolated from HAP40KO cells. Proteins enriched from HTTKO extracts were excluded. (*D*) GO enrichment analysis of biological process (BP) and (*E*) cellular component (CC) terms comparing HTT-interacting proteins from WT and HAP40KO HEK293 cells (as defined in *C*, excluding HTTKO background). Terms were identified using the *enrichGO* package in R and ranked by Benjamini–Hochberg FDR-adjusted *P*-values.

We hypothesized that binding of HAP40 to HTT^Q23^ might influence its association with other proteins in cells. To uncover HTT^Q23^ interaction partners, we employed a coimmunoprecipitation-mass spectrometry (co-IP-MS) approach, which enables the detection of PPIs with high specificity and sensitivity using label-free quantification (37). A pilot experiment with anti-HTT (D7F7) antibody demonstrated efficient enrichment of the HTT–HAP40 complex from protein extracts (Fig. 3*A*). Building on this, we performed comprehensive co-IP-MS experiments with extracts from HEK293 WT, HEK293-HTTKO, and HEK293-HAP40KO cells (Fig. 3*B*). HEK293-HTTKO extracts were used to evaluate nonspecific antibody binding. Relative protein abundances were quantified in antibody-enriched samples using intensity-based absolute quantification (iBAQ) (Dataset S1).

Principal component analysis (PCA) revealed that protein abundance profiles from D7F7-enriched samples clustered well within biological replicates (*SI Appendix*, Fig. S7*A*). Furthermore, PCA showed that sample groups display HAP40 dependency, indicating that HTT^Q23^ in the presence and absence of HAP40 associates with different sets of cellular proteins. To identify specific apo-HTT interactors, we evaluated protein enrichment by statistical significance (Fig. 3*C* and *SI Appendix*, Fig. S7 *B* and *C*). Only proteins that were reproducibly enriched (adj. *P*-value < 0.05) with D7F7 from WT or HEK293-HAP40KO protein extracts but not from HEK293-HTTKO extracts were considered as HTT interaction partners (*SI Appendix*, Fig. S7 *B* and *C*). With these criteria, we detected a total of 542 unique HTT^Q23^-associated proteins, of which 149 were exclusively coenriched from WT and 262 from HEK293-HAP40KO protein extracts (Fig. 3*C*). Notably, 131 proteins (~20%) were shared between both groups, indicating that the absence of HAP40 significantly alters HTT’s interaction landscape. Compared with the HTT^Q23^–HAP40 complex, apo-HTT bound a greater number of proteins, suggesting that it is more interaction-prone and aberrantly associates with various cellular proteins (Fig. 3*C*). Gene Ontology (GO) term enrichment analysis supports this conclusion. Apo-HTT associated with a diverse array of functionally distinct proteins, including those involved in mitochondrial processes, intracellular transport, and lipid biosynthesis (Fig. 3 *D* and *E*). In contrast, proteins involved in ribosome biogenesis and rRNA metabolism were predominately coenriched with the HTT^Q23^–HAP40 complex (Fig. 3 *D* and *E*). KEGG pathway enrichment analysis yielded similar results (*SI Appendix*, Fig. S7 *D* and *E*). Cluster analysis of high-confidence interactors (adj. *P*-value < 0.001) also confirmed two distinct groups: one preferentially associated with HTT^Q23^–HAP40 and the other with apo-HTT (*SI Appendix*, Fig. S7*F*), whereby the apo-HTT group was larger. GO term enrichment analysis revealed that apo-HTT-bound proteins are involved in mitochondrial and transport processes, whereas the HTT^Q23^–HAP40-bound proteins are involved in filament assembly and trafficking pathways (*SI Appendix*, Fig. S7*G*). Collectively, these data suggest that HTT^Q23^ in complex with HAP40 associates with proteins involved in cytoplasmic translation, ribosome biogenesis, and vesicle trafficking, while in the absence of HAP40 it binds to proteins involved in dispersed subcellular processes. To validate these findings, we used a BRET-based binding assay to assess the impact of HAP40 loss on HTT interactions. We focused on the E3 ubiquitin-protein ligase RNF126 (38), which was enriched with apo-HTT (*SI Appendix*, Fig. S7*F*). Using HEK293 WT and HEK293-HAP40KO cells, we coproduced NL-HTT^Q23^ and PA-mCit-RNF126 and quantified *in-cell* BRET. The BRET signal was significantly higher in HEK293-HAP40KO compared to WT cells, confirming that RNF126 preferentially interacts with apo-HTT in the absence of HAP40 (*SI Appendix*, Fig. S7*H*).

Finally, co-IP-MS experiments of HTT using striatal cell extracts, demonstrated a similar shift in GO enrichment terms upon the loss of HAP40, where HTT–HAP40 enriched from ST*Hdh*^Q7^ or ST*Hdh*^Q111^ cells predominantly associated with proteins involved in ribosome biogenesis and translation, while apo-HTT enriched from ST*Hdh*^Q7^-*Hap40*KO or ST*Hdh*^Q111^-*Hap40*KO cells associated with other proteins (*SI Appendix*, Fig. S8 *A*–*C*). Additionally, the extent of shared HTT interactors in striatal and HEK293 cells was examined. Comparative analysis of enriched proteins with HTT–HAP40 identified 68 common interactors in HEK293 WT and ST*Hdh*^Q7^ cells (*SI Appendix*, Fig. S8*D* and Dataset S2). Most of these proteins belong to pathways related to ribosomal biogenesis and translation (*SI Appendix*, Fig. S8*E*). Comparison of shared interactors in HEK293 and striatal cells lacking HAP40 revealed seven common proteins (*SI Appendix*, Fig. S8*F*) such as BAG2, DERL1, or DNAJB6 that play functional roles in stress and protein quality control pathways.

### Loss of HAP40 Decreases the Abundance of HTT-Associated Proteins in HEK293 Cells.

To assess global proteomic changes resulting from HAP40 or HTT knockout in HEK293 cells, we performed Tandem Mass Tag (TMT)-based mass spectrometry for total proteomes across HEK293 WT, HAP40KO, and HTTKO cell lines (*SI Appendix*, Fig. S9*A* and Dataset S3). PCA of TMT datasets revealed distinct clustering per genotype. HEK293-HAP40KO samples exhibited a moderate shift along both PCs, while HEK293-HTTKO samples displayed a substantial divergence (*SI Appendix*, Fig. S9*B*). For example, HEK-HAP40KO cells showed ~3,000 significantly downregulated proteins, while HEK-HTTKO cells exhibited a markedly larger effect, with ~4,000 proteins downregulated (adj. *P*-value ≤ 0.05; *SI Appendix*, Fig. S9 *C* and *D*). These data underscore the profound proteome-wide consequences of losing either HAP40 or HTT. To explore the functional relevance of these changes, we performed Ingenuity Pathway Analysis (IPA) on the significantly downregulated proteins in each KO line. Interestingly, in both HEK293-HTTKO and HEK293-HAP40KO cells proteins involved in rRNA processing and eukaryotic translation were significantly reduced in abundance (*SI Appendix*, Fig. S9*E*).

We next examined the abundance of proteins identified as HTT^Q23^–HAP40 interactors in our co-IP-MS experiments. TMT intensity data revealed a significant reduction in their abundance in both KO lines (*SI Appendix*, Fig. S9*F*). To determine whether this subset of proteins was disproportionately decreased in abundance, we performed Fisher’s exact tests comparing the proportion of significantly downregulated proteins in the interactor group vs. the background proteome. In HEK293-HAP40KO cells, ~50% of the HTT^Q23^–HAP40-associated proteins were significantly decreased in abundance, compared to 27% in the background proteome (Fisher’s exact test, *P*-value < 0.05). Similarly, in HEK293-HTTKO cells, ~60% of the subgroup was significantly reduced in abundance in comparison to ~30% of the background (*SI Appendix*, Fig. S9*G*). We observed similar trends among apo-HTT^Q23^-associated proteins (*SI Appendix*, Fig. S9*H*). This pattern is notably also pronounced among the high-confidence HTT-enriched proteins (*SI Appendix*, Fig. S9 *I*–*L*). Together these findings suggest that HTT–HAP40 heterodimers, through PPIs, function as stabilizers for associated proteins in mammalian cells.

### Loss of HAP40 Leads to Widespread Transcriptional Changes and Upregulation of CLEAR Pathway-Associated Proteins in Striatal Cells.

**Fig. 4.**
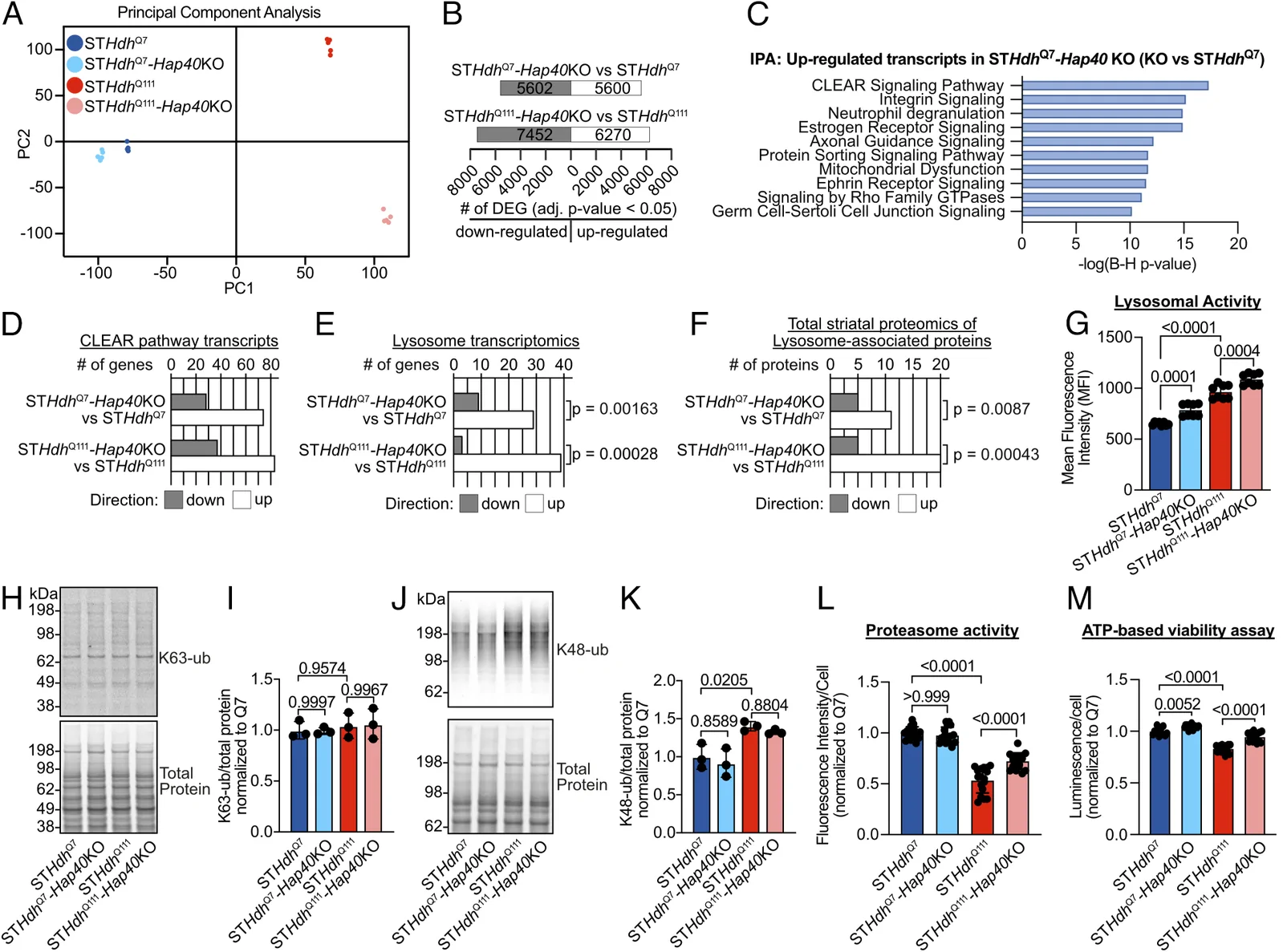
Transcriptional and lysosomal remodeling in WT and mutant HTT-expressing striatal cells lacking HAP40. (*A*) PCA of transcriptome profiles from ST*Hdh*^Q7^, ST*Hdh*^Q7^-*Hap40*KO, ST*Hdh*^Q111^, and ST*Hdh*^Q111^-*Hap40*KO cell lines. Each point represents a biological replicate (n = 4) and is colored by genotype. The plot visualizes global transcriptional differences among the conditions. (*B*) Numbers of significantly upregulated (white) and downregulated (gray) transcripts (adj. *P*-value < 0.05) in the two *Hap40*KO lines relative to their respective parental controls. The total number of DEGs is shown in each bar. (*C*) IPA of transcripts significantly upregulated (adj. *P*-value ≤ 0.05) in ST*Hdh*^Q7^-*Hap40*KO vs. ST*Hdh*^Q7^ cells. Canonical pathways are ranked by Benjamini–Hochberg FDR-adj. *P*-values. (*D*) Panel shows the number of genes that belong to the CLEAR network and are either increased or decreased in the two different striatal knockout cell lines compared with their respective parental lines. For each genotype, ST*Hdh*^Q7^-*Hap40*KO and ST*Hdh*^Q111^-*Hap40*KO, we identified transcripts whose expression changed significantly (adj. *P*-value ≤ 0.05). These transcripts were then matched against a curated list of CLEAR-pathway genes taken from the IPA database. The resulting counts of upregulated and downregulated CLEAR genes are presented side-by-side for each knockout condition. (*E*) Number of lysosomal genes significantly up- or downregulated in the two *Hap40*KO lines relative to controls. Upregulation of lysosomal genes is significantly overrepresented relative to downregulation, in contrast to the up/down-distribution observed in the total dataset (one-sided Fisher’s exact test). (*F*) Same analysis as in *E* but showing the number of lysosomal proteins quantified by proteomics. (*G*) Lysosomal activity measured by flow cytometry and mean fluorescence intensity (MFI) was assessed. Data are presented as mean ± SD of two independent experiments with four technical replicates of 10,000 cells each. Statistical significance was evaluated using one-way ANOVA with Šídák’s multiple comparisons test. (*H*) Representative immunoblot of mouse striatal total lysates probed for K63-linked ubiquitin. (*I*) Quantification of K63-linked ubiquitin levels relative to total protein and normalized to ST*Hdh*^Q7^, related to *H*. (*J*) Representative immunoblot of mouse striatal total lysates probed for K48-linked ubiquitin. For (*H* and *J*) no-stain labeling as protein loading control. (*K*) Quantification of K48-linked ubiquitin levels relative to total protein and normalized to ST*Hdh*^Q7^, related to *J*. For *I* and *K*, data are presented as mean ± SD from three biological replicates and statistical significance was assessed by one-way ANOVA with Tukey’s multiple comparison test. (*L*) Quantification of proteasome activity assay performed with mouse striatal cell lines. Data are presented as mean ± SD of four independent experiments with four technical replicates each. Statistical significance was assessed by one-way ANOVA with Bonferroni’s multiple comparison test. (*M*) Quantification of ATP-based viability based on the luminescence signal relative to the number of cells per well, normalized to ST*Hdh*^Q7^. Data represent the mean ± SD of two independent experiments, each with 7 to 8 technical replicates. Statistical significance was assessed by two-way ANOVA with Bonferroni’s multiple-comparisons test.

We next investigated the effects of HAP40 loss on gene expression programs in striatal cells. PCA of transcriptome datasets revealed divergent gene expression profiles between ST*Hdh*^Q7^ and ST*Hdh*^Q111^ cells, with further divergence observed upon HAP40 loss in each background (Fig. 4*A*). Differential gene expression analysis revealed >11,000 differentially expressed genes (DEGs) (adj. *P*-value < 0.05) when datasets of ST*Hdh*^Q7^ and ST*Hdh*^Q7^-*Hap40*KO cell lines were compared (Fig. 4*B* and Dataset S4), indicating that HAP40 loss exerts a profound impact on the transcriptome. Notably, the number of DEGs was significantly higher in the mutant background, implying that cells producing HTT^Q111^ may experience greater transcriptional stress when HAP40 is absent. IPA of DEGs of ST*Hdh*^Q7^ and ST*Hdh*^Q7^-*Hap40*KO cells revealed dysregulation across a wide range of pathways and cellular processes (Fig. 4*C*). Among the top significantly upregulated pathways in ST*Hdh*^Q7^-*Hap40*KO and ST*Hdh*^Q111^-*Hap40*KO cells (Fig. 4*C* and *SI Appendix*, Fig. S10*A*), we identified the CLEAR gene network (Fig. 4*D*), which is induced by the transcription factor TFEB (39) governing autophagy and lysosomal biogenesis (40, 41). This suggests that HAP40 in striatal cells functions as a proteostasis regulator and its loss activates protein degradation pathways. Closer inspection of CLEAR network-associated genes confirmed the upregulation of genes encoding key lysosomal proteins (Fig. 4*E*). The downregulated transcriptional pathways mostly affected in ST*Hdh*^Q7^-*Hap40*KO cells were related to the mitotic machinery and the RHO GTPase cycle. In comparison, in ST*Hdh*^Q111^-*Hap40*KO cells signaling pathways controlling axonal guidance and extracellular processes were downregulated (*SI Appendix*, Fig. S10 *B* and *C*). We also repeated the IPA with more stringently selected gene expression datasets (cut-off |LFC| > 0.5 and adj. *P*-value < 0.05; *SI Appendix*, Fig. S10 *D*–*H* and Dataset S5), revealing similar dysregulated pathways. This analysis also showed the activation of CLEAR pathway genes in ST*Hdh*^Q111^-*Hap40*KO cells (*SI Appendix*, Fig. S10*G*), confirming its relevance in striatal *Hap40*KO cells. Strikingly, cellular fractionation experiments confirmed that the nuclear abundance of the transcription factors TFEB and TFE3, which are known to activate CLEAR pathway genes (39), is increased in ST*Hdh*^Q111^-*Hap40*KO in comparison to ST*Hdh*^Q111^ cells (*SI Appendix*, Fig. S10 *I*–*K*), supporting the hypothesis that HAP40 loss activates CLEAR pathway genes. In contrast, no such a HAP40-dependent effect was observed with the transcription factor MITF (*SI Appendix*, Fig. S10*L*).

Furthermore, we performed a total proteome analysis on the striatal cell lines (*SI Appendix*, Fig. S11 *A*–*C* and Dataset S6) and observed increased levels of lysosomal proteins in ST*Hap40*KO in comparison to control cells (Fig. 4*F*). Importantly, a cross-data comparison of proteomic and RNA-seq datasets demonstrated that a large fraction of lysosomal proteins and transcripts are concordantly changed (*SI Appendix*, Fig. S11 *D* and *E* and Dataset S7). Thus, our omics analyses suggest that HAP40 loss in striatal cells transcriptionally activates the autophagy-lysosome pathway (ALP) (42), which is also supported by measurements of lysosomal activity in ST*Hdh*^Q7^-*Hap40*KO and ST*Hdh*^Q111^-*Hap40*KO cell lines (Fig. 4*G*).

In comparison, biochemical studies revealed that the abundance of K63-linked ubiquitin chains was not significantly altered by HAP40 loss in striatal cells (Fig. 4 *H* and *I*). However, the abundance of K48-linked ubiquitin chains was significantly higher in ST*Hdh*^Q111^ compared to ST*Hdh*^Q7^ cells, suggesting that proteasome activity is impaired in HTT^Q111^-expressing striatal cells. Indeed, quantification of proteasome-activity revealed reduced activity in ST*Hdh*^Q111^ compared to ST*Hdh*^Q7^ cells (Fig. 4*L*), corroborating the immunoblotting result (Fig. 4 *J* and *K*). However, a slight increase in proteasome-activity was observed in ST*Hdh*^Q111^-*Hap40*KO compared to ST*Hdh*^Q111^ cells (Fig. 4*L*), indicating that loss of HAP40 also weakly activates the ubiquitin proteasome system (UPS) in ST*Hdh*^Q111^-*Hap40*KO cells. This is also supported by IPA, indicating that similar to the CLEAR pathway (Fig. 4*D*), the UPS is also transcriptionally activated in ST*Hdh*^Q111^-*Hap40*KO cells (*SI Appendix*, Fig. S10*A*).

Last, we quantified ATP levels to assess whether loss of HAP40 in ST*Hdh*^Q111^-*Hap40*KO cells and cellular toxicity are associated. We found that ATP levels were significantly increased in ST*Hdh*^Q111^-*Hap40*KO in comparison to control ST*Hdh*^Q111^ cells (Fig. 4*M*), suggesting that activation of transcriptional programs such as CLEAR through HAP40 loss improves the energetic state of striatal cells. Together these results indicate that HAP40 functions as a transcriptional proteostasis regulator that controls protein degradation pathways and ATP levels in striatal cells.

### Loss of HAP40 Leads to an Accumulation of the Autophagy-Associated Proteins p62, LC3B-II, and STX17 in ST*Hdh*^Q111^-*Hap40*KO Cells.

**Fig. 5.**
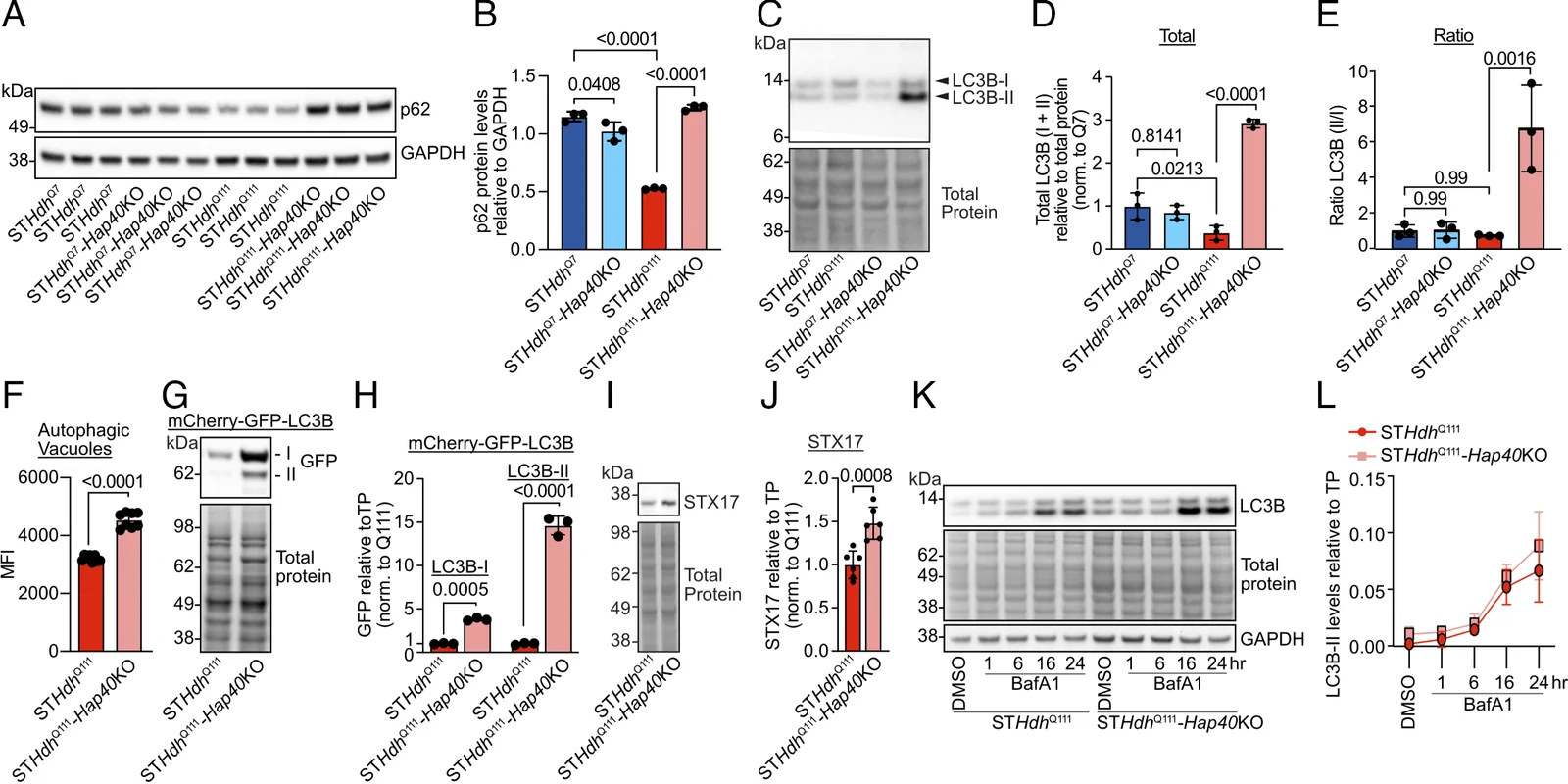
Loss of HAP40 results in impaired autophagosome-lysosome flux. (*A*) Representative immunoblots of mouse striatal total cell lysates. Membranes were probed with antibodies against p62 and the loading control GAPDH. (*B*) Quantification of p62 protein levels in *A*, expressed relative to GAPDH. Data are presented as mean ± SD of four biological replicates. Statistical significance was determined by one-way ANOVA with Tukey’s multiple comparisons test. (*C*) Representative immunoblot of mouse striatal lysates probed for LC3B with total protein staining as loading control. (*D*) Quantification of total LC3B I & II protein bands relative to total protein and (*E*) Quantification of ratio of LC3BII/LC3BI, both related to *C*. For both (*D* and *E*) data are presented as mean ± SD of three biological replicates. Statistical significance was determined by one-way ANOVA with Tukey’s multiple comparisons test. (*F*) Quantification of autophagic vacuoles was performed using an autophagy assay kit and measured by flow cytometry. Data are presented as the MFI ± SD of two independent experiments, each with four technical replicates of 10,000 cells. Statistical significance was assessed by one-way ANOVA with Tukey’s multiple comparisons test. (*G*) Representative immunoblot of mouse striatal lysates transfected with pDEST-mCherry-GFP-LC3B and probed using an anti-GFP antibody, with total protein staining as loading control. (*H*) Quantification of total mCherry-GFP-LC3B-I and -II protein bands relative to total protein (TP). Data are presented as mean ± SD of three biological replicates. Statistical significance was assessed by Welch’s *t* test. (*I*) Representative immunoblot of striatal lysates probed with anti-STX17, and total protein staining. (*J*) Quantification of STX17 relative to TP; data are presented as mean ± SD of three biological replicates. Statistical significance was assessed by Welch’s *t* test. (*K*) Representative immunoblot of mouse striatal lysates treated with 100 nM Bafilomycin A1 (BafA1) or an equal volume of DMSO for various time points. (*L*) Measurement of LC3B-II levels relative to TP and across BafA1 treatment timepoints (n = 2).

We explored whether changes in lysosomal activity (Fig. 4*G*) may influence the abundance of the autophagy receptor p62 (43), whose steady state levels are controlled by the ALP (44). Analysis of protein extracts by SDS-PAGE and immunoblotting revealed that p62 levels are slightly lower in ST*Hdh*^Q7^-*Hap40*KO than in ST*Hdh*^Q7^ cells (Fig. 5 *A* and *B*). Interestingly, significantly higher p62 levels were quantified in ST*Hdh*^Q7^ than in ST*Hdh*^Q111^ cells, suggesting that HTT^Q111^ enhances p62 degradation through ALP (Fig. 5*B*). However, to our surprise we observed that steady state levels of p62 in ST*Hdh*^Q111^-*Hap40*KO cells in comparison to ST*Hdh*^Q111^ cells were significantly increased (Fig. 5 *A* and *B*), indicating that HAP40 loss in cells with pathogenic HTT^Q111^ impairs autophagy and leads to accumulation of p62, although lysosome activity is high (Fig. 4*G*).

To assess whether loss of HAP40 in ST*Hdh*^Q111^-*Hap40*KO cells alters autophagic flux (45), we next quantified the lipidated marker protein LC3B-II (46), which measures the abundance of autophagic vesicles in neuronal cells (47). Analysis of protein extracts by SDS-PAGE and immunoblotting revealed significantly higher LC3B-II levels and a higher LC3B-II/I ratio in ST*Hdh*^Q111^-*Hap40*KO than in ST*Hdh*^Q111^ cells (Fig. 5 *C*–*E*), indicating that autophagic flux is impaired and autophagic vesicles accumulate in HTT^Q111^-producing striatal cells lacking HAP40. This was also confirmed by IFM studies showing a significantly higher number of LC3B-positive puncta in ST*Hdh*^Q111^-*Hap40*KO than in ST*Hdh*^Q111^ control cells (*SI Appendix*, Fig. S12 *A* and *B*). Furthermore, treatment of cells with a fluorescent dye that selectively labels autophagic vacuoles revealed higher signals in ST*Hdh*^Q111^-*Hap40*KO than in ST*Hdh*^Q111^ cells (Fig. 5*F* and *SI Appendix*, Fig. S12*C*), supporting our initial observations that autophagic vesicles accumulate in *Hap40*KO cells. To quantify autophagic LC3B degradation, we also expressed a tandem monomeric mCherry-GFP-tagged LC3B (48) fusion protein in striatal cells. Notably, higher amounts of mCherry-GFP-LC3B (both I and II forms) were detectable in ST*Hdh*^Q111^-*Hap40*KO cells compared to ST*Hdh*^Q111^ control cells (Fig. 5 *G* and *H*), confirming that autophagic protein degradation is decreased in the absence of HAP40. A key step in the delivery of cargo to the lysosome is the fusion of autophagosomes with lysosomes. This step is facilitated by STX17, an autophagosome SNARE protein, which is recruited to mature autophagosomes (49). Immunoblotting revealed increased STX17 levels in ST*Hdh*^Q111^-*Hap40*KO compared to ST*Hdh*^Q111^ cells (Fig. 5 *I* and *J*). Also, a reduced association of STX17 with the lysosome marker protein LAMP1 was observed by IFM (*SI Appendix*, Fig. S12 *D* and *E*), supporting the hypothesis that turnover of late autophagic compartments is impaired in HTT^Q111^-producing cells, when HAP40 is absent.

Finally, investigations with Bafilomycin A1 (BafA1), a specific V-type H+-ATPase inhibitor (50) that blocks autophagosome-lysosome fusion (51) revealed that the kinetics of LC3B-II accumulation in striatal *Hap40*KO cells is not significantly altered compared to controls (Fig. 5 *K* and *L*). This indicates that HAP40 loss in HTT^Q111^ striatal cells does not alter autophagosome biogenesis. Together, these studies suggest that HAP40 function is critical for autophagic protein degradation in ST*Hdh*^Q111^-*Hap40*KO cells and its loss leads to an accumulation of LC3B-II-positive-autophagic vesicles.

### Loss of HAP40 Promotes the Release of Pathogenic HTT^Q111^ into the Extracellular Space.

**Fig. 6.**
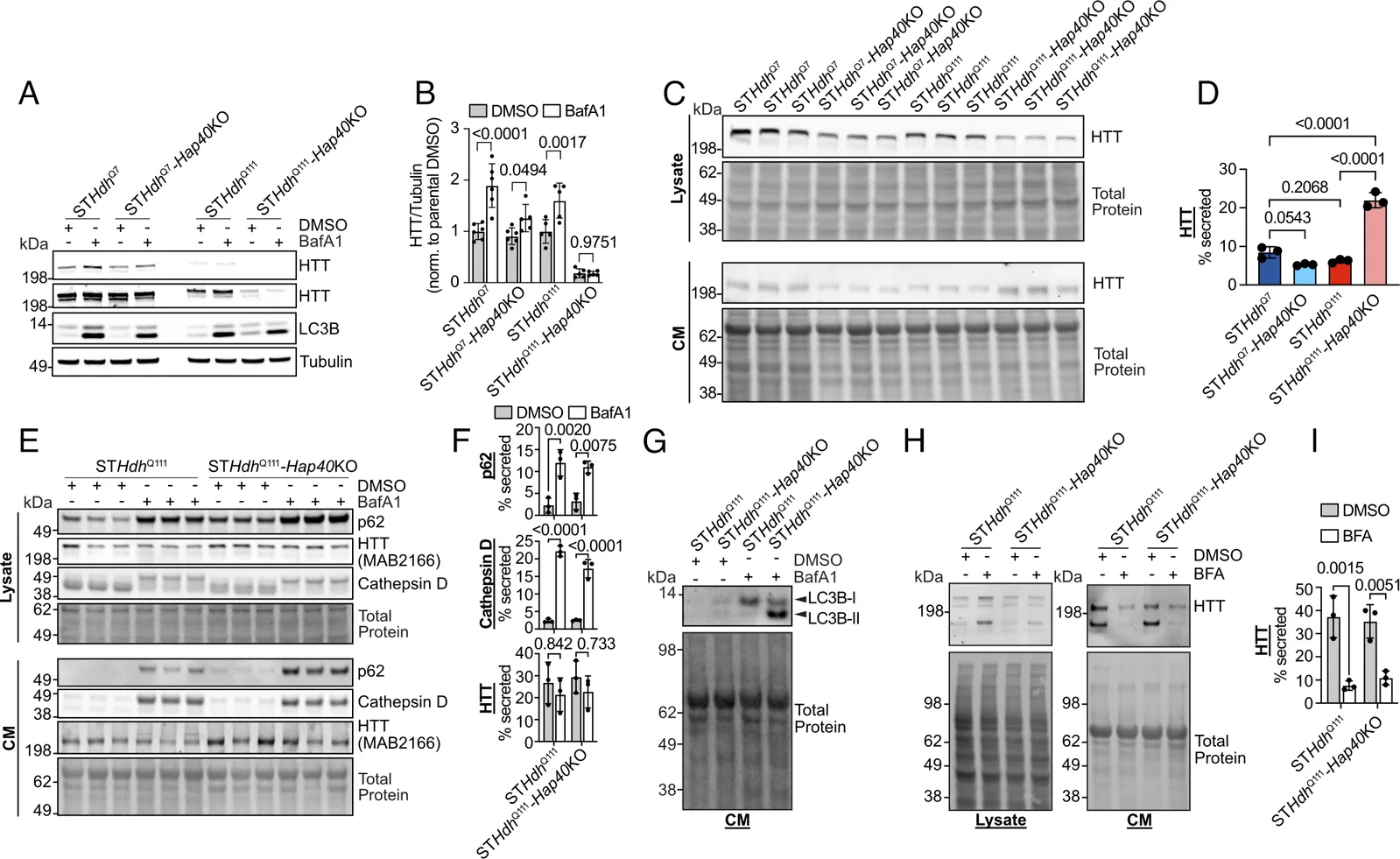
Loss of HAP40 stimulates mHTT secretion in ST*Hdh*^Q111^-*Hap40*KO striatal cells. (*A*) Representative immunoblots of total cell lysates treated with DMSO or 100 nM BafA1 for 24 h. (*B*) Quantification of HTT protein levels relative to tubulin, related to *A*. Data were normalized to parental control (either ST*Hdh*^Q7^ or ST*Hdh*^Q111^). Data are presented as mean ± SD of two independent replicates, each with three biological replicates. Statistical significance was assessed by two-way ANOVA with Bonferroni’s multiple-comparisons test. (*C*) Representative immunoblots of striatal lysates and CM, probed with anti-HTT (MAB2166) and total protein staining. (*D*) Quantification assessing the percentage (%) of secreted protein in CM. Data are presented as mean ± SD of three biological replicates. Statistical significance was assessed by one-way ANOVA with Tukey’s multiple comparisons test.(*E*) Immunoblot of striatal lysates and CM in the presence of 100 nM BafA1 or DMSO for 24 h, probed with anti-p62, anti-HTT (MAB2166), anti-Cathepsin D, and total protein staining. (*F*) Quantification assessing the percentage (%) of secreted protein in CM. Data are presented as mean ± SD of three biological replicates. Statistical significance was assessed by two-way ANOVA with Tukey’s multiple comparisons test. (*G*) Representative immunoblot of striatal CM treated for 24 h with 100 nM BafA1 or equal volume of DMSO, probed with anti-LC3B and total protein staining. (*H*) Representative immunoblots of striatal lysates and CM in the presence of 5 µg/mL Brefeldin A (BFA) or DMSO for 4 h, probed with anti-HTT (MAB2166) and total protein staining. (*I*) Quantification assessing the percentage (%) of HTT secreted protein in CM. Data are presented as mean ± SD of three biological replicates. Statistical significance was assessed by two-way ANOVA with Tukey’s multiple comparisons test.

Previous studies indicate that full-length HTT gets readily degraded by ALP in cells (52, 53). Therefore, we next investigated the impact of HAP40 loss on HTT degradation by this pathway. Striatal cell lines were first treated with BafA1 to assess HTT accumulation when autophagosome-lysosome fusion is blocked (46). As expected, we observed a significant increase of HTT^Q7^, when ST*Hdh*^Q7^ or ST*Hdh*^Q7^-*Hap40*KO cells were treated with BafA1 (Fig. 6 *A* and *B*), confirming previous observations that the ALP degrades HTT (54). Similarly, an increase of HTT^Q111^ was observed in BafA1-treated ST*Hdh*^Q111^ cells, while such an effect was not detected in ST*Hdh*^Q111^-*Hap40*KO cells (Fig. 6 *A* and *B*), indicating that this protein is not cleared through a BafA1-sensitive autophagy route (55). Thus, alternative pathways such as secretory autophagy (56) might be responsible for decreasing HTT^Q111^ abundance in striatal *Hap40*KO cells.

To address this question, we next prepared protein fractions from striatal cells and conditioned media (CM) and analyzed them by SDS-PAGE and immunoblotting using an anti-HTT (MAB2166) antibody. As expected, we observed that HTT^Q7^ protein levels in ST*Hdh*^Q7^-*Hap40*KO cells are significantly lower than in ST*Hdh*^Q7^ cells (Fig. 6*C*), confirming our initial observations (Fig. 1*D*). A similar result was obtained when cell extracts of ST*Hdh*^Q111^-*Hap40*KO cells were analyzed. Strikingly, in conditioned medium of ST*Hdh*^Q111^-*Hap40*KO cells we detected significantly higher amounts of HTT^Q111^ than in controls (Fig. 6 *C* and *D*). Importantly, low levels of HTT^Q7^ were also released in ST*Hdh*^Q7^ cells, demonstrating that both mutant and WT HTT are secreted. However, loss of HAP40 in WT cells did not exaggerate HTT^Q7^ secretion, suggesting that most WT protein gets degraded intracellularly. Cumulatively, these studies demonstrate that pathogenic HTT^Q111^ in the absence of HAP40 is not degraded by canonical autophagy. However, upon HAP40 loss its secretion into the extracellular space is significantly enhanced. To assess whether full-length HTT or HTT fragments are secreted, cell extracts and CM were investigated with SDS-PAGE and immunoblotting (*SI Appendix*, Fig. S13 *A* and *B*). Surprisingly, we found that rather than full-length HTT (~350 kDa), a truncated ~205 kDa HTT fragment was released into the extracellular space. This fragment was detected with the MAB2166 antibody, which recognizes residues 445 to 459 in HTT, as well as the MW1 antibody that binds the polyQ tract (57). This indicates that striatal cells release a C-terminally truncated polyQ-containing HTT fragment into the extracellular space. Interestingly, in addition to the ~205 kDa HTT fragment also two smaller N-terminally truncated HTT fragments lacking the polyQ domain (~145 kDa and ~135 kDa) were detectable in the conditioned medium, suggesting that both N- and C-terminally truncated HTT fragments are released into the extracellular space.

To determine the secretory pathway (58) responsible for HTT^Q111^ secretion, we employed chemical compounds that inhibit different secretory pathways. We first treated cells with BafA1, a potent inhibitor of lysosome activity that stimulates secretory autophagy (56). We observed that BafA1 treatment neither influences HTT^Q111^ secretion in ST*Hdh*^Q111^-*Hap40*KO nor in ST*Hdh*^Q111^ cells (Fig. 6 *E* and *F*), indicating that stimulation of secretory autophagy does not influence HTT^Q111^ release. However, BafA1 treatment significantly increased the secretion of p62, cathepsin D, and LC3-II under these experimental conditions (Fig. 6 *E*–*G*), confirming previous observations (56).

Finally, we investigated whether Brefeldin A (BFA)-sensitive ER/Golgi-associated trafficking influences HTT^Q111^ secretion. Striatal cells were treated with BFA, which blocks conventional ER/Golgi-associated protein secretion (59, 60). Treatment with this compound potently decreased the abundance HTT^Q111^ in the conditioned medium (Fig. 6 *H* and *I*), suggesting that molecular events in a BFA-sensitive ER/Golgi-associated trafficking pathway are critical for mHTT release. Finally, we knocked down *Sec13* gene expression in striatal ST*Hdh*^Q111^-*Hap40*KO cells with siRNA to validate the relevance of the ER/Golgi trafficking route for releasing mHTT. The function of SEC13 is essential for protein sorting out of the ER and the secretion of cargo through the ER/Golgi pathway (61). We found that knock-down of *Sec13* decreased the release of mHTT into the extracellular space (*SI Appendix*, Fig. S13 *C* and *D*), supporting our results with BFA treated cells. Thus, an ER/Golgi-associated secretory pathway is likely critical for releasing mHTT from striatal cells.

## Discussion

In this study, we investigated the function of the α-helical, tetratricopeptide repeat protein HAP40, including its influence on HTT interactions and mHTT pathobiology. Utilizing imaging and biochemical methods, we found that HAP40 is an obligate HTT interaction partner under endogenous conditions (Fig. 1*H*), meaning that it is permanently associated with HTT and controls its function through binding. One aspect of HTT is that it was reported to bind thousands of proteins (62, 63), a unique feature that was attributed to its ability to operate as a Swiss-army knife. To date, there are no interaction studies that have assessed the impact of HAP40 on HTT interactions. Our investigations revealed that apo-HTT in the absence of HAP40 binds more cellular proteins than the HTT–HAP40 complex (Fig. 3*C*), supporting the hypothesis that HAP40 binding to HTT controls its association with other cellular proteins. We suggest that HAP40 regulates HTT interactions by stabilizing its 3D conformation and defining its cellular localization. Strikingly, utilizing the sensor protein NL-HTT^Q23(2689-mCit)^ and quantitative *in-cell* BRET measurements, we here show that HAP40 binding stabilizes HTT conformation in mammalian cells (Fig. 2 *A*–*D*). Additionally, loss of HAP40 altered the subcellular distribution of full-length HTT. While HTT–HAP40 complexes in HEK293 cells were concentrated in the perinuclear region, the distribution of apo-HTT was more dispersed (*SI Appendix*, Fig. S2*M*), suggesting that it has the potential to interact with many other cellular proteins. We propose that HAP40 binding enables HTT to localize to specific subcellular processes and to expose interaction surfaces that are critical for its molecular function.

Our GO term enrichment analysis of interaction datasets supports the hypothesis that apo-HTT and HTT–HAP40 complexes bind distinct proteins in mammalian cells. While proteins involved in transmembrane transport processes, lipid biosynthesis, or mitochondrial functions (Fig. 3*D*) were found to predominantly associate with apo-HTT, proteins involved in ribosome biogenesis and rRNA metabolism were preferentially coenriched with HTT–HAP40 heterodimers. We propose that multiprotein complexes with HTT–HAP40 fulfill specific functional tasks in cells, while protein complexes with apo-HTT are potentially deleterious, because they are nonphysiological or unregulated interactions. Hence, the association of apo-HTT with proteins implicated in mitochondrial functions may contribute to cellular dysfunction and stress, while interactions of HTT–HAP40 heterodimers with proteins involved in translation and/or ribosome biogenesis likely are functionally relevant. An association of HTT with proteins involved in translation has been reported previously (64, 65). Furthermore, functional investigations in cell models (66) have shown that full-length HTT directly binds RNA (67) and facilitates protein synthesis (64, 66).

Our proteomics studies further support the notion that HAP40 functions as a HTT PPI regulator. The comparison of HTT interaction and proteomics datasets revealed that a large fraction of identified HTT–HAP40-associated proteins is significantly decreased in abundance in HEK293-HAP40KO and HEK293-HTTKO cells (*SI Appendix*, Fig. S9 *F*–*L*), indicating that HTT–HAP40 heterodimers stabilize interacting proteins in mammalian cells. A prime example is the neurofilament medium protein NEFM, which is enriched with the HTT–HAP40 complex in co-IP experiments, but upon loss of HTT or HAP40, its abundance is decreased in cells. A previous proteomic study in a HD mouse model also showed a correlation of NEFM and HTT abundance, supporting our finding (63).

Analysis of gene regulatory networks and biochemical studies position HAP40 as proteostasis regulator. The transcriptome profiles of HAP40 KO striatal cell lines revealed a massive transcriptional dysregulation of thousands of genes leading to reprogramming of multiple cellular pathways (Fig. 4*B*). We observed that the transcriptional dysregulation in HTT^Q111^-producing HAP40 KO cells was significantly more pronounced than in ST*Hdh*^Q7^-*Hap40*KO cells (Fig. 4*B* and *SI Appendix*, Fig. S10*D*), indicating that, in the absence of HAP40, mHTT is more proteotoxic and induces a stronger transcriptional response. Intriguingly, our analysis revealed an activation of the CLEAR network (Fig. 4*C* and *SI Appendix*, Fig. S10 *A* and *G*), which regulates the activity of the ALP in mammalian cells (39). We suggest that HAP40 through binding HTT may function as proteostasis regulator that controls protein degradation. This view is also supported by our functional studies with HAP40 KO and control striatal cell lines, demonstrating that loss of HAP40 significantly increases lysosome activity (Fig. 4*G*). In addition, we observed an abnormal accumulation of the autophagy marker proteins p62, LC3-II, and STX17 in ST*Hdh*^Q111^-*Hap40*KO cells (Fig. 5 *A*–*J*), supporting our hypothesis that autophagic protein degradation is impaired. Importantly, previous investigations indicating that HTT plays a functional role in selective autophagy (21, 22) are in agreement with our observations. In summary, we propose that HAP40 serves as a proteostasis regulator by controlling HTT interactions and gene regulatory networks. In this function it maintains the steady-state levels of thousands of cellular proteins and governs the activity of lysosomal genes (*SI Appendix*, Fig. S14).

The activation of lysosome-based protein clearance pathways (Fig. 4*G*) may explain our observations that the abundance of full-length HTT is significantly lower in cells without HAP40 than in controls (Fig. 1 *D* and *E*). Surprisingly, however, our experimentation showed that autophagic flux in ST*Hdh*^Q111^-*Hap40*KO cells is impaired and mutant HTT^Q111^ likely is not cleared through ALP in HAP40 KO striatal cells (Fig. 6 *A* and *B*). As previous investigations showed that cells can secrete mHTT (68, 69), we hypothesized that secretion may decrease HTT^Q111^ levels in striatal cells. Strikingly, our biochemical studies showed that ST*Hdh*^Q111^-*Hap40*KO cells indeed release significantly higher amounts of HTT^Q111^ than control cells (Fig. 6 *C* and *D*), supporting the idea that secretory pathways are activated in HAP40 KO cells. Interestingly, the release of mHTT into the extracellular space was blocked when striatal cells were treated with the secretion inhibitor BFA (Fig. 6 *H* and *I*), which inhibits ARF1 activation at ER and Golgi membranes (70) and disrupts protein trafficking from the ER to the Golgi (71). Thus, we suggest that an ER/Golgi-associated secretory route is critical for the release of mHTT into the extracellular space. This is also supported by siRNA knockdown of *Sec13* (*SI Appendix*, Fig. S13 *C* and *D*), a critical COPII coat protein, and our observations that HTT is associated with Golgi membranes in striatal cells (*SI Appendix*, Fig. S4). Furthermore, HTT function was shown to be required for efficient ER-to-Golgi transport and the fusion of secretory vesicles with the plasma membrane (72). The release of mHTT fragments into the extracellular space was recently also found to be influenced by knockdown of the Golgi-associated structural protein GRASP55 (73). However, more detailed investigations are necessary to elucidate the specific molecular events that facilitate the secretion of polyQ-containing HTT fragments in the extracellular space.

We propose that our observations with striatal cell lines have important implications for a better understanding of the disease mechanism in HD and the development of future therapeutic strategies. Our finding that HAP40 functions as a proteostasis regulator in cells, determining the steady-state levels of thousands of cellular proteins, suggests that therapeutic strategies targeting HAP40 for protein lowering, such as siRNAs or ASOs (74), may not be a beneficial strategy. Although lowering HAP40 could lead to a decrease in mHTT levels, it poses the risk of also reducing the levels of many other cellular proteins. Moreover, decreasing HAP40 protein levels with therapeutic molecules might activate comprehensive transcriptional programs and stress pathways to readjust protein homeostasis. Additionally, knock-down of HAP40 in neurons could also promote the release of mHTT into the extracellular space, which in turn might stimulate mHTT spreading, a phenomenon that has been observed in *Drosophila* (75) and cell models (76). Spreading of misfolded disease proteins such as tau or α-synuclein is associated with dysfunction and neurotoxicity in neurodegenerative diseases, including Alzheimer’s disease (AD), Parkinson’s disease (PD), and amyotrophic lateral sclerosis (ALS) (77, 78). Taken together, our findings define HAP40 as a proteostasis regulator that is required to stabilize canonical HTT interactions and to mitigate mHTT proteotoxicity.

## Materials and Methods

Human HEK293 and mouse striatal cell lines were engineered using CRISPR-Cas9 genome editing. PPIs and cellular phenotypes were evaluated using confocal microscopy and quantitative BRET assays. Proteomic profiling was conducted through quantitative mass spectrometry and transcriptional landscapes were mapped via stranded mRNA sequencing. Bioinformatic and pathway enrichment analyses were performed using DESeq2, limma, clusterProfiler, and shinyGO. Complete protocols and experimental details are provided in *SI Appendix*.

## Supplementary Material

Appendix 01 (PDF)

Dataset S01 (XLSX)

Dataset S02 (XLSX)

Dataset S03 (XLSX)

Dataset S04 (XLSX)

Dataset S05 (XLSX)

Dataset S06 (XLSX)

Dataset S07 (XLSX)

## Data Availability

Study data, including transcriptomic and proteomic datasets, are included in the article and/or supporting information.
