# Supplementary material for: HAP40 functions as a proteostasis regulator by controlling huntingtin interactions and its release into the extracellular space
Source: Proc Natl Acad Sci U S A. 2026 Oct 22;123(43):e2611301123. doi: 10.1073/pnas.2611301123 (PMC13715816; doi:10.1073/pnas.2611301123)
Supplement: Supplementary file 1 [file pnas.2611301123.sapp.pdf]

PNAS

Supporting Information for

HAP40 functions as a proteostasis regulator by controlling huntingtin interactions and its release into the extracellular space

Eduardo Silva Ramos, Annett Boeddrich, Christian Haenig, Philipp Trepte, Orchid Ammar, Christopher Secker, Oliver Popp, Andranik Ivanov, Stephanie Beetz, Leonard Roth, Roxane M. Papawassiliou, Luisanna C. Hernandez Jeppesen, Theo Wallenfang, Brandon Keith, Rachel J. Harding, Franziska Schindler, Tomas Koudelka, Nadine Scharek, Martina Zenkner, Nancy Neuendorf, Sabrina Golusik, Philipp Mertins, Ilaria Piazza, Sigrid Schnoegl, Erich E. Wanker*

*Corresponding author, Erich E. Wanker
Email: erich.w@mdc-berlin.de

This PDF file includes:

- Materials and Methods
- Figures S1 to S14
- Legends for Datasets S1 to S7
- Supplementary References

Other supporting materials for this manuscript include the following:

- Datasets S1 to S7

Materials and Methods

Cell lines

Human embryonic kidney line 293 (HEK293) wild-type and HEK293-HTTKO cells (1) were grown in Dulbecco's modified Eagle's medium (DMEM) GlutaMAX (Thermo Fisher Scientific, 31966047) supplemented with 10% heat-inactivated fetal bovine serum (FBS) (Thermo Fisher Scientific, 10500064), 1x MEM non-essential amino acids (NEAA) (Thermo Fisher Scientific, 11140035), and 1% penicillin-streptomycin (P/S) (Thermo Fisher Scientific, 15140122) at 37°C, and 5% CO_2. Mouse striatal (ST) Hdh^{Q7} (ST$Hdh^{Q7/Q7}$, CH00097) and STHdh^{Q111} (ST$Hdh^{Q111/Q111}$, CH00095) neuronal cell lines (2) were obtained from the HD Community Biorepository at the Coriell Institute. Striatal cells were maintained in complete DMEM medium (Thermo Fisher Scientific, 41965) supplemented with 10% FBS, 1% P/S, 1x NEAA, 0.4 mg/mL geneticin (Thermo Fisher Scientific, 10131035) and incubated at 33°C and 5% CO_2. After thawing, cells were cultured for one week to reach log phase before cell pellets were collected or experiments were performed. Cells were subcultured every three to four days.

CRISPR-Cas9 engineering

The HEK293-HAP40KO-clone1B4 (HEK293-HAP40KO) cell line was generated using CRISPR-Cas9 genome editing to induce indels close to the start triplet 'ATG' of the human *HAP40* (*F8A1*) gene. A single gRNA (sgRNA) sequence (ACCCGAGGCCGGGGACTTCC) was cloned into pSpCas9(BB)-2A-Puro (Addgene plasmid #62988) and was used for gene editing and selection of cells expressing the puromycin cassette (Puro). One million cells were seeded into a six-well plate and 2 µg of plasmid was transfected into cells using FuGENE transfection reagent (Promega, E2311) at a 3:1 (µL:µg) reagent-to-DNA ratio. 24 hours later, cells were selected for uptake of the Cas9 plasmid by adding puromycin (1 µg/mL final). After 3 days of puromycin selection, single cells were obtained by serial dilution into 96-well plates, one cell per every three wells. Single-cell colonies were expanded and PCR amplification was performed targeting the edited site using the following primers, forward primer: CTTTAGCAACCTAGACCAC; reverse primer: CACATGA-GGAGTACAAGAGTAG. The expected PCR product was purified and subjected to Sanger sequencing with the same PCR oligos. Sequencing confirmed that the clonal cell line had a frameshift mutation resulting in a premature stop codon. Immunoblot analysis using 25 µg of total cell lysate and an anti-HAP40 antibody (Atlas, HPA046960,1:500) confirmed the loss of full-length HAP40 expression in KO clones.

The HEK293 HTT-mNeonGreen-clone1B (HEK293-HTT-mNG) knock-in cell line was generated using a similar approach with CRISPR-Cas9 editing. In this case, a single guide RNA (sgRNA) targeting the 3' end of the human *HTT* gene (CCACCTGCTGAGCGCCATGG) was co-transfected with a custom-designed homology-directed repair template. This template contained a human codon-optimized mNeonGreen gene flanked by 500 base pairs of the HTT insertion site. Cells that exhibited mNeonGreen fluorescence were collected via fluorescence-activated cell sorting and serially diluted to obtain single-cell colonies. These colonies were subjected to PCR amplification using the following primers: GCTGGAGCAGGTGGACGTGAAC and TCTGGAA-GGCCTCAGGCTCAGC. An unedited clone produced an amplified DNA fragment of approximately 416 base pairs, while an edited knock-in clone amplified a 1154 base-pair fragment. Clones displaying only the 1154 base-pair band (indicating homozygous knock-in) were further analyzed by Sanger sequencing. Immunoblot analysis using total cell lysates and the anti-HTT D7F7 antibody (Cell Signaling, 5656, 1:1000) confirmed the increased molecular weight of HTT. Additionally, confocal microscopy using an anti-mNeonGreen antibody (Chromotek, 32F6, 1:500) confirmed the expression of the HTT-mNG fusion protein.

Mouse STHdh^{Q7}-*Hap40*KO-clone21 (STHdh^{Q7}-*Hap40*KO) and STHdh^{Q111}-*Hap40*KO-clone18 (STHdh^{Q111}-*Hap40*KO) cell lines were generated using CRISPR-Cas9 genome editing. The strategy for genetic editing employed two sgRNA sequences expressed from different pSpCas9(BB)-2A-Puro plasmids. The two gRNA sequences were: TCTGCGTCCTCCTTGGGCGG and CTTCTTGGCACGCTATCGGC. Each sgRNA induces a double-strand break at a distinct position near the start of the mouse *Hap40* gene, producing a deletion of up to 50 base pairs that results in a frameshift and a premature stop codon. The transfection and selection workflow was similar to that described above. Here, 150,000 cells were seeded into a six-well plate and 1 µg of

each plasmid was transfected into cells using FuGENE transfection reagent at a 3:1 (µL:µg) reagent-to-DNA ratio. Selection was performed with puromycin (2 µg/mL for ST*Hdh*^{Q7} and 4 µg/mL for ST*Hdh*^{Q111}). Single-cell colonies were screened by PCR for the 50 bp deletion using the knockout primer pair CATTTGCGTCACTCAAGCCC (forward) and CAAGAAGTCCCCAG-CCTCTG (reverse). An unedited clone yielded an amplified fragment of approximately 361 bp, whereas a successfully edited knockout clone produced no detectable product. Clones confirmed as knockouts were subjected to a second PCR with primers flanking the edited region (forward CGGAAGCGGAGGTGATGAAT, reverse CACGTTCGGCTTCCTCAAGA). Sanger sequencing of this amplicon verified the intended deletion and demonstrated a frameshift mutation that generates a premature stop codon. Chromatogram analysis was performed using Benchling.com software. Finally, immunoblot analysis of total cell lysates with the anti-HAP40 antibody (Atlas, HPA046960, 1:500) confirmed the loss of full-length HAP40 expression in the knockout clones.

Immunofluorescence

HEK293 and mouse striatal cells were cultured in DMEM GlutaMAX supplemented with 10% FBS, 1x NEAA, and 1% P/S; striatal cells were additionally supplemented with 0.4 mg/mL geneticin. HEK293 and mouse striatal cells were detached, counted, and then seeded on either uncoated coverslips (mouse striatal cells) or on fibronectin/poly-L-lysine-coated coverslips (HEK293). For coverslip coating, 12 mm coverslips in 24-well plates were washed once with PBS (Thermo Fisher Scientific, 14190-094) and then incubated with fibronectin (Sigma, F1141) and poly-L-lysine (Sigma, P8920), both diluted 1:100 in ddH2O, for four hours at 37°C. Following this incubation, the coverslips were washed once with ddH2O and once with PBS before the cells were seeded on top. HEK293 and mouse striatal cells were seeded between 20,000 to 25,000 cells per well. Cells were fixed 48 hours after seeding as follows: coverslips were washed once with PBS and incubated with 4% PFA (Thermo Fisher Scientific, 8908) for 10 min at room temperature. The PFA was removed, cells were washed 3x with PBS and then permeabilized using 0.01% Triton-X 100 (Sigma Aldrich, T9284) for 10 min at room temperature. After permeabilization, the cells were incubated with Hoechst (Thermo Fisher Scientific, H3570, 1:10,000 in PBS) for 2 to 5 min at room temperature. After washing the cells, the coverslips were incubated for one hour at room temperature with 3% bovine serum albumin (BSA) (Sigma, A3059-10G) in PBS for blocking. After blocking, the cells were incubated overnight at 4°C with primary antibodies against the proteins of interest diluted in 3% BSA: rabbit anti-HAP40 (Atlas, HPA046960,1:500), rabbit anti-LC3B (Abcam, ab192890, 1:500), mouse anti-mNeonGreen (Chromotek, 32F6, 1:500), anti-HTT MAB2166 (Millipore, MAB2166, 1:500), mouse anti-Vimentin (Sigma, L6630, 1:1000), rabbit anti-HDAC6 (Proteintech, 128341-AP, 1:500), anti-Phalloidin iFluor 647 (Abcam, ab176759, 1:1000), rabbit anti-GOLGA2 (Proteintech, 11308-1-AP, 1:1000), mouse anti-STX17 (Thermo Fisher, 24GB15930, 1:500), rat anti-LAMP1 (Santa Cruz, sc-19992, 1:200). The next day, primary antibodies were removed, cells were washed 5x with the 3% BSA solution and then incubated for two hours at room temperature with a combination of the following secondary antibodies diluted 1:500 in PBS: Alexa Fluor 488 (Thermo Fisher Scientific, A11006, A11008 or A11001), Alexa Fluor 647 (Thermo Fisher Scientific, A21245 or A21236), nano-secondary anti-rabbit A568 (Proteintech, srbAF568-1), nano-secondary anti-mouse A568 (Proteintech, sms1AF568-1), nano-secondary anti-mouse A647 (Proteintech, sms1AF647-1). The coverslips were washed 5x with PBS and then mounted onto microscopy slides using DAKO mounting medium (Agilent Technologies, S302380-2) and left to dry overnight at room temperature before imaging.

Microscopy

HEK293 and mouse striatal cells were imaged using a Leica TCS SP8 WLL confocal microscope or a Zeiss LSM980 confocal microscope equipped with Airyscan2. All samples were imaged using the 63x oil immersion objective. Images were taken as Z-stacks with an average of 15-25 steps (SP8) or between 50-70 steps (LSM980) and a system-optimized Z-step size. To capture images from samples labelled with Hoechst and fluorescent secondary antibodies, the following lasers were used: diode laser (405 nm), argon-ion laser (488 nm), 561 nm laser, or a HeNe laser (633 nm). Imaging the mNG signal in HEK293-HTT-mNG cells was done using the argon-ion laser for fluorophore excitation and the emission was captured at 500-570 nm. Sequential scanning was applied; in some cases, red or far-red acquisition was combined with Hoechst acquisition. Hoechst

and HDAC6 were imaged in the same track using the 405 nm and 561 nm diode lasers, and emission was captured at 379-486 and 596-679 nm, respectively. Hoechst and Vimentin were imaged in the same track using the 408 nm and 561 nm diode lasers; emission was captured at 379-486 and 565-679 nm, respectively. Hoechst and Phalloidin-iFluor 647 signals were imaged in the same track using 408 nm and 639 nm diode lasers, respectively; detection was set at 379-539 nm and 586-758 nm, respectively. Samples were scanned as a line average of 2 with a pixel size of 0.071 µm.

Image Analysis

Colocalization analysis

All images within an experimental group were processed with the same parameters using the Fiji software (3). The co-localization of two proteins of interest was analyzed qualitatively and quantitatively. For the former, a line scan analysis was performed by first drawing a line across a region of interest (ROI) using a Z-projected image (sum of slices). ROI was used for all channels and the “plot profile” analysis tool was applied for each channel. The values from all channels were then overlapped by plotting the gray values of the pixels against the distance in µm on the line. Quantitative analysis of colocalization was done by calculating the Pearson and Manders (M1 and M2) coefficients using the JACoP plugin in Fiji (4). Here, channels from Z-stacked images were first split; then, for each channel, “median filters” and “subtract background” processing tools were applied. Next, the JACoP plugin was activated, green and red channels were assigned to images A and B, and the threshold was set for each channel separately according to the signal intensities. The coefficients obtained from this analysis were plotted on a bar graph using GraphPad Prism.

LC3B puncta analysis

All images taken of striatal cells were processed in the same way using the Fiji software, unless otherwise stated. First, median filters were manually applied to the split channels, and the background signals were reduced. The channels were then merged again, and a Z-project was created using the maximum intensities of all Z positions. Look-up tables were assigned to each channel, then the images were analyzed. For LC3B puncta count and size analysis, single cells were cropped out of images and analyzed separately to obtain the number and size of LC3B puncta per cell. The signal threshold for the LC3B channel was manually set according to the observed signals; then, using the “Analyze particles” tool in Fiji, the number and area of the puncta were measured. For particle analysis, the size in µm^2 was set to 0-infinity, the circularity was set to 0.0-1.0, and particles touching the borders of the image were excluded by selecting the option “exclude on edges”. The average count and size of puncta per cell were calculated and plotted on bar graphs using GraphPad Prism. Statistical analysis using a one-way ANOVA was also performed using GraphPad Prism.

Perinuclear enrichment index and corrected total cell fluorescence

All images within an experimental group were processed with the same parameters using the Fiji software. First, a Z-project image (sum of slices) was generated for each channel. Next, the signal threshold for the Phalloidin and Hoechst channels was manually set according to the observed signals and converted to an 8-bit mask. The “Analyze particles” tool was applied with the size range set to 15-infinity µm^2. Only whole cells and respective nuclear regions detected by the tool were saved as ROIs. Individual nuclear ROIs were then subjected to the “enlarge” tool (edit > selection > enlarge) where they were enlarged by 20 pixels and this enlarged ROI was saved. Next, the nuclear and enlarged-nuclear ROIs were both selected in the ROI manager and the XOR tool (more > XOR) was used to craft a new ROI representing a perinuclear ring. The final ROIs were three background spots where no cells were present. Using the ROI manager, the phalloidin ROI (whole cell), the perinuclear ring ROI, and background ROIs were “measured” to record the area-integrated density and mean gray value. The data were exported and analyzed in the following manner. To determine the perinuclear enrichment index, the perinuclear-ring mean gray value was divided by the whole-cell mean gray value. A PEI value approaching one is considered to have a more diffused signal across the cell, whereas a large value indicates a higher degree of perinuclear enrichment. To determine the corrected total cell fluorescence, the average mean gray value of the three

background ROIs was multiplied by the whole-cell area. This value was then subtracted from the whole-cell integrated density to obtain the corrected total cell fluorescence.

Cell treatments

Striatal cells were plated in 6-well dishes at a density of 200,000-300,000 cells per well in 2 mL of complete medium and incubated overnight at 33°C. The following day, drug treatment, 100 nM Bafilomycin A1 solubilized in DMSO (Cell Signaling, 54645S) or 5 µg/mL Brefeldin A (Cell Signaling, 9972), was applied in complete DMEM or Opti-MEM (secretion experiments) medium for the times indicated in the figure legends.

Viability assay

Striatal cells were seeded into a transparent 96-well plate at a density of 10,000 cells per well in 100 µL of complete medium. The next day, the number of cells was determined by staining cells with Hoechst 33342 (Thermo Fisher Scientific, H3570) at a final concentration of 5 µg/mL in complete medium for 15 min at 33°C. Afterwards, wells were washed with PBS and imaged using a Tecan Spark Cyto device. Next, the CellTiter-Glo 2.0 cell viability assay (Promega, G924) was used by applying 25 µL of CellTiter-Glo per well. The plate was mixed using an orbital plate shaker for 2 min at 700 rpm and incubated for 10 min at room temperature. Total luminescence was measured with a Tecan Spark Cyto device with an integration time of 0.25-1 sec per well.

Protein secretion assay and trichloroacetic acid precipitation

Striatal cells were seeded into a 6-well plate at a density of 200,000-300,000 cells per well in 2 mL of complete medium and incubated overnight at 33°C. The following day, cells were washed 3x with warm PBS and 1.5 mL of warm Opti-MEM medium was added to each well. At this point, for certain experiments, drug treatment was performed (see cell treatments); otherwise, plates were incubated at 33°C for 24 hr. After this incubation period, all conditioned medium was collected in 2 mL tubes and placed on ice. Cell pellets were collected by trypsinization, neutralization with medium, and washed with PBS before freezing . For these steps, centrifugation was performed at 700 x g for 5 min at 4°C. Collected conditioned medium was first centrifuged at 700 x g for 10 min at 4°C and the supernatant was transferred to a new 2 mL tube. Samples were centrifuged again at 2,000 x g for 20 min at 4°C and 1,370 µL was transferred to a new 2 mL tube. Proteins in precleared conditioned medium were precipitated by addition of 151 µL of 100% TCA. Samples were inverted and incubated for 10 min on ice. Afterwards, 500 µL of ice-cold 10% TCA was added, inverted to mix, and incubated for 20 min on ice. Samples were then centrifuged at 20,000 x g for 10 min at 4°C. Supernatant was carefully removed and samples incubated with 1 mL ice-cold acetone overnight at -20°C. The next day, samples were centrifuged at 20,000 x g for 10 min at 4°C; acetone was carefully removed. Samples were left to air-dry before addition of 12 uL of sample loading buffer (100 mM DTT, 1x NuPAGE LDS sample buffer, diluted with TNT-lysis buffer). In parallel, the respective cell pellets were lyzed in 15 µL of TNT-lysis buffer (20 mM Tris-HCl pH 8.0, 150 mM NaCl, 1% Triton-X). Total cell lysates and conditioned medium were subjected to SDS-PAGE and immunoblotting together.

Proteasomal activity assay

Striatal cells were seeded into a transparent 96-well plate at a density of 10,000 cells per well in 100 µL of complete medium and incubated overnight at 33°C. The next day, the number of cells per well was determined by staining a designated set of cells with Hoechst 33342 (Thermo Fisher Scientific, H3570) at a final concentration of 5 µg/mL in complete medium for 15 min at 33°C. Afterwards, wells were washed with PBS and imaged using a Tecan Spark Cyto device; eight technical replicates were assessed per genotype to obtain a mean value. Next, the 20S Proteasome Assay Kit (Cayman Chemical, Cay10008041-1) was used for assessing proteasome activity. First, the plate was centrifuged at 500 x g for 5 min, medium was discarded from a separate designated set of wells, and 200 µL of 20S proteasome assay buffer was added to each well. Then, the plate was centrifuged at 500 x g for 5 min, the buffer was discarded, and 100 µL of 20S proteasome assay lysis buffer was added. The plate was incubated in an orbital shaker at 300 rpm for 30 min at room temperature. Afterwards, the plate was centrifuged at 1000 x g for 10 min at room temperature. Then 90 µL of supernatant was transferred to a clear-bottom black 96-well plate.

Next, 40x substrate was diluted to 1x using assay buffer, and 10 µL of solution was added to each well. The plate was incubated in an orbital plate shaker set to 300 rpm for one hour at 37°C. Fluorescence readout was performed with a Tecan Spark Cyto using the following settings: fluorescent intensity; Ex/360 nm, Em/480 nm.

Lysosomal activity assay

Striatal cells were seeded in 6-well plates at a density of 200,000-300,000 cells per well in 2 mL of complete medium and incubated overnight at 33°C. The next day, the medium was replaced with 1 mL of pre-warmed DMEM supplemented with 0.5% FBS, 1x Pen/Strep, and 15 µL of the self-quenched substrate from the Abcam Lysosomal Intracellular Activity Assay Kit (ab234622); all solutions were protected from light. Labeled cells were incubated for 1 hour at 33°C, then detached with trypsin, examined microscopically to confirm a single-cell suspension, and neutralized with complete medium. The cell suspension was transferred to a 1.5 mL microcentrifuge tube, gently resuspended with a 1 mL pipette tip to break up any remaining clumps and centrifuged at 700 x g for 3 min. The pellet was washed twice with 1 mL FACS buffer (PBS with 1% FBS), repeating the centrifugation step each time. Finally, the cells were resuspended in 400 µL cold FACS buffer and kept on ice until flow-cytometric acquisition.

Autophagic vacuoles labelling

Striatal cells in logarithmic growth were allowed to reach 70-80% confluence. They were then washed with PBS, detached with trypsin, and resuspended in complete medium. After cell counting, 200,000 viable cells were allocated to each condition (treated and untreated). The cells were pelleted at 700 x g for 3 min and washed with phenol-free DMEM containing 5% FBS. A labeling mixture was prepared by adding 1 µL of the green reagent from the Autophagy Detection Kit (Abcam 139484) to 1 mL of phenol-free DMEM with 5% FBS. The cell pellets were resuspended in 250 µL of labeling solution or just medium (FACS negative control) and incubated for 30 min at 33°C, protected from light. Following incubation, the samples were centrifuged again at 700 x g for 3 min and washed twice with ice-cold FACS buffer. The final pellet was resuspended in 400 µL of cold FACS buffer and kept on ice until flow-cytometric acquisition.

Flow cytometric analysis

Samples were analyzed using a BD LSR Fortessa cell analyzer equipped with 488 nm (530 / 30 BP) and 561 nm (710 /50 BP) lasers. Initial gating used forward-scatter versus side-scatter (FSC-A vs SSC-A) to isolate the cell population, and doublets were excluded by plotting FSC-H vs FSC-W followed by SSC-H vs SSC-W. The final gated population was evaluated on the 488 nm versus 561 nm channels. Untreated cells served as negative controls to define background fluorescence and set the final gates. Data acquisition was performed with FACSDiva software, collecting at least 10,000 live events per sample. Post-acquisition analysis was carried out in FlowJo v10 using the same gating strategy to obtain mean fluorescence intensity values; statistical comparisons were made with GraphPad Prism v9.

Western blotting

Cell pellets were lysed in 30 to 50 µL HEPES lysis buffer (50 mM HEPES pH 7.0, 150 mM sodium chloride, 10% glycerol, 1% NP-40, 20 mM NaF, 1.5 mM $MgCl_2$, 1 mM EDTA, 1 mM PMSF, 0.5% sodium deoxycholate, 1x Benzonase, 1x cOmplete EDTA-free protease inhibitor cocktail (Merck, 5056189001) for 30 min on ice. Lysates were centrifuged at 14,000 rpm for 10 min at 4°C and supernatants collected. For experiments where detection of LC3B was required, samples were lysed for 5 min on ice in LC3-lysis buffer containing Dulbecco's phosphate-buffered saline, 2% Triton-X, 1x cOmplete EDTA-free protease inhibitor cocktail and cleared by centrifugation at 16,000 x g for 10 min at 4°C. Samples treated with LC3-lysis buffer were lysed and subjected to SDS-PAGE on the same day. Protein concentrations were determined by BCA assay (Thermo Fisher Scientific, 23227) and 20-25 µg total protein was combined with 50 mM DTT and 1x NuPAGE LDS sample buffer, followed by 5 min at 95°C. Proteins and protein standards (Thermo Fisher Scientific, LC5925) were separated by SDS-PAGE using a NuPAGE 4-12% Bis-Tris gel with 1x MES buffer and wet-transferred onto nitrocellulose membranes (Cytiva, 10600002) using transfer buffer (25 mM Tris, 192 mM glycine, 20% methanol) at 30V for two hours. For certain experiments, SDS-

PAGE was performed using 3-8% Tris-Acetate gel with 1x Tris-Acetate SDS running buffer (Thermo Fisher Scientific, LA0041) and the HiMark pre-stained protein standard (Thermo Fisher Scientific, LC5699). After transfer, membranes were stained with No-Stain protein labelling reagent (Thermo Fisher Scientific, A44717) following manufacturer's instructions before blocking. Membranes were then blocked for one hour in 3% milk in PBS with 0.05% Tween. The following primary antibodies were applied overnight at 4°C: rabbit anti-HAP40 (Atlas Antibodies, HPA046960, 1:1000), anti-HTT D7F7 (Cell Signaling, 5656, 1:1000), anti-HTT MAB2166 (Millipore, MAB2166, 1:500), anti-HTT MW1 (DSHB, MW1, 1:500), mouse anti-SQSTM1(p62) (abcam, ab56416, 1:1000), rabbit anti-LC3B (abcam, ab192890, 1:1000), rabbit anti-K48-ubiquitin (Abclonal, A3606, 1:1000), rabbit anti-K63-ubiquitin (Cell Signaling, 5621, 1:1000), mouse anti-HTT (MW1) (DSHB, MW1, 1:1000), rabbit anti-SEC13 (PTG,15397-1-AP, 1:1000), rabbit anti-Cathepsin D (Cell Signaling 69854, 1:1000), rabbit anti-STX17 (PTG 17815-1-AP, 1:1000), mouse anti-FLAG (Sigma, F3165, 1:1000), mouse anti-mNeonGreen (Chromotek, 32F6, 1:200), rabbit anti-GFP (Abcam, ab290, 1:2500), mouse anti-c-Myc (Merck, M4439, 1:2000), mouse anti-Actin (abcam, ab8224, 1:1000), mouse anti-Tubulin (Sigma, T6074, 1:80,000), mouse anti-TIM23 (BD Biosciences, 611223, 1:1000), and mouse anti-GAPDH (Santa Cruz, sc-47724, 1:1000). The following POD-conjugated secondary antibodies were diluted 1:2000 in 3% milk-PBS with 0.05% Tween and applied for one hour at room temperature: goat anti-Rabbit IgG peroxidase (Sigma, A0545), goat anti-mouse IgG peroxidase (Sigma, A0168). In certain cases, the following secondary antibody was applied at 1:2000 for one hour at room temperature in 1x fluorescence blocking solution (Thermo Fisher Scientific, 37565): IRDye 800CW Donkey anti-Rabbit IgG (Li-Cor, NC0964679) or IRDye 680RD Donkey anti-Mouse IgG (Li-Cor, NC0963034). Each membrane was incubated with WesternBright Quantum (advanstar, K-12042-D20) solution for 2 min, followed by acquisition of a chemiluminescence image using an iBright imaging system (Thermo Fisher Scientific). Band intensities were quantified using Fiji (ImageJ) and normalized to the respective loading control within each corresponding lane. Statistical analysis was performed using Prism v9 software.

Co-Immunoprecipitation

Cell pellets were lysed in IP lysis buffer (25 mM Tris pH 7.5, 150 mM NaCl, 5% glycerol, 1% IGEPAL (NP-40),1x MS-SAFE protease, and 1x phosphatase inhibitor) for 30 min on ice. Lysates were centrifuged at 14,000 rpm for 10 min at 4°C and supernatants were collected. Mouse brain tissue (400 mg) was homogenized using a Precellys device (5000 rpm, 2x 20 sec, 15 sec break) with 1 mL ice-cold 50 mM Tris pH 7.5. The volume of homogenates was adjusted to a 10-fold excess with 1x RIPA Buffer (50 mM Tris pH 7.5, 150 mM NaCl, 1% Triton X-100, 0.1% SDS, 0.5% sodium deoxycholate, EDTA-free protease inhibitor and Benzonase 0.25 U/µL) and incubated for one hour on a rotating wheel at 4°C. Protein concentrations were determined by BCA assay (Thermo Fisher Scientific, 23227); about one mg of lysate was used per co-IP experiment. Immunoprecipitation experiments were performed using the Pierce Crosslink Immunoprecipitation kit (Thermo Fisher Scientific, 26147) in combination with Dynabeads Protein G (Thermo Fisher Scientific, 10004D) following manufacturer's instructions. Dynabeads were resuspended by vortexing for 30 seconds. For each co-IP reaction, 12.5 µL of beads was transferred to an individual 2 mL LoBind microtube. Beads were placed on the magnetic stand and storage liquid was discarded, followed by two washing steps with 200 µL 1x coupling buffer. For antibody crosslinking to protein G, beads were resuspended in 100 µL 1x coupling buffer together with the respective amount of antibody as determined in antibody-bead saturation experiments (anti-HTT D7F7 antibody, Cell Signaling, 5656, 7 µL) and incubated at room temperature under constant agitation for 60 min. Afterwards, beads were briefly pulse-centrifuged up to 500 x g, followed by magnetizing the beads and removing the supernatant. Beads were then washed twice with 200 µL 1x coupling buffer, magnetized, and supernatant was discarded. Next, 2.5 µL of 20x coupling buffer, 9 µL of 2.5 mM DSS in DMSO, and 38.5 µL MiliQ water was added to each bead preparation and placed under constant agitation for 60 min at room temperature. Beads were then magnetized, supernatant was removed, and beads were washed with 3x 500 µL elution buffer. Beads were then washed 3x with 500 µL lysis buffer. Then, lysis buffer was removed, and 1 mg of cell lysate in a final volume of 500 µL lysis buffer was added to the beads. A volume corresponding to 25 µg of protein was set aside from the cell lysate and used as input sample for downstream western blotting. Samples were

incubated overnight at 4°C on a rotating wheel for gentle end-over-end mixing. Afterwards, beads were magnetized and washed 2x with 500 µL wash buffer (0.05 % IGEPAL (NP-40), 50 mM Tris pH 7.5, 150 mM NaCl, 5% glycerol) followed by 2x washing with 500 µL IGEPAL-free wash buffer. For antigen elution, beads were resuspended in 24 µL 1x LDS sample buffer containing 100 mM DTT and incubated at 95°C for 10 min. Beads were then magnetized and supernatant was collected. Eluate samples were used immediately for SDS-PAGE or stored at -20°C.

Size Exclusion Chromatography (SEC)

HEK293 cells were prepared in the following manner. A pellet of 80 million cells was resuspended in 800 µL lysis buffer containing HEPES, 150 mM NaCl, 1 mM EDTA, 1 mM $MgCl_2$, 1 mM DTT, 0.1% NP40, protease inhibitor cocktail (cOmplete, EDTA-free; Roche, 05056489001) and phosphatase inhibitor (Pierce, A32957). Cell suspensions were mixed by pipetting up and down, and by vortexing. Lysis was supported by freezing cell suspensions on dry ice for 5 min, followed by thawing on ice. Then 20 U benzonase (1:10,000, Merck, 1016540001) were added and lysates were incubated for 30 min on ice. Lysates were centrifuged at 10,000 x g for 15 min at 4°C. Mouse brains (12-month-old C57BL/6 wild-type) were prepared in the following manner: Brain samples (5 mg) were lysed utilizing a manual homogenizer in lysis buffer (50 mM HEPES pH 7.4,150 mM NaCl, 1.5 mM $MgCl_2$,1 mM EDTA, 1 mM DTT, protease inhibitor cocktail, Benzonase 1:10,000). The volume of lysis buffer used was 10x the weight of tissue. Lysates were centrifuged at 15,000 rpm for 15 min at 4°C. For both cells and mouse brain lysates, supernatant was transferred to a new tube and protein concentration was determined using BCA Protein Assay (Pierce, 23228).

For SEC, an Äkta pure 25 chromatography system with a Superose 6 Increase 10/300 GL column (Cytiva, GE29-0915-96) at a flow rate of 0.5 ml/min was used. The column was equilibrated in running buffer (50 mM HEPES, 150 mM NaCl, 1 mM EDTA, 1 mM $MgCl_2$, 1x cOmplete Protease Inhibitor cocktail, 1 mM DTT). For calibration of the column, high- and low-molecular-weight standard proteins spanning a molecular weight range of 669 – 13.7 kDa (Cytiva, 28403842 and 28403841) were used. Supernatant of cell lysate (2.5 mg protein) was loaded onto the column and fractionated. Pairs of consecutive fractions of 400 µL fractions were pooled prior to precipitation. For protein precipitation, a fourfold volume of cold-acetone (99.7%) was added to the pooled fractions, mixed and incubated overnight at -20°C. The next day, proteins were precipitated by centrifugation at 3,428 x g at 4°C for 45 min. The supernatant (~95%) was carefully taken off and disposed. The residual solution was centrifuged again at 3,428 x g at 4°C for 5 min. The remaining supernatant was removed again without disturbing the protein pellet. The pellet was dried for 5 min, resuspended in 100 µL gel loading buffer (1x NuPage LDS sample buffer, 50 mM DTT) and heat-denatured at 95°C for 5 min. Samples were subjected to SDS-PAGE using NuPAGE Novex 4-12% Bis-Tris gels (Thermo Fisher Scientific, NP0329) followed by transfer of proteins onto a nitrocellulose membrane (0.45 µm; Cytiva, 10600002) using a wet blotting system (BioRad). Membranes were blocked and washed as described in Western blot section. Immunoblotting using anti-HTT D7F7 (Cell Signaling, 5656S, 1:1000) and anti-HAP40 (Atlas Antibody, HPA 046960, 1:500) antibodies in 3% milk/PBS was performed, followed by labelling with secondary peroxidase-conjugated anti-rabbit antibody (1:2000) for one hour. Membranes were incubated with WesternBrightTM Quantum (advanstar, K-12042-D20) solution; acquisition of a chemiluminescence image was performed using an iBright imaging system (Thermo Fisher Scientific).

Native filter retardation assay

HEK-HTT-mNG cells were seeded into a 96-well plate and transfected using Lipofectamine 2000 with pdEYFP-N1-HTTex1Q49-eYFP, pdEYFP-N1-HTTex1Q23-eYFP or pcDNA3.1. After 48 hours, media was removed and cells were lysed in 50 µL buffer containing 5% glycerol, 25 mM Tris pH 7.5, 150 mM NaCl, 1 mM MgCl2, 1% NP40, 1x protease inhibitor, and benzonase (1:10,000) on ice for 30 min. Then, 10 µg of total protein was dotted onto a cellulose acetate membrane in quadruplicates, filtered, and washed three times with 100 µL PBS. The membrane was then equilibrated in PBS, fluorescence EYFP signals were acquired using the iBright imaging system. Full-length and exon1 HTT were detected using rabbit anti-HD1 (in-house (5), 1:2000) following standard immunoblotting procedures.

Nucleus and cytoplasm fractionation

Subcellular fractionation into nuclear and cytoplasmic fractions was performed using a modified version of a protocol described by Senichikin et al. (6). Unless otherwise stated, all fractionation steps were carried out in Protein LoBind microcentrifuge tubes (Eppendorf). DTT, protease inhibitors, and phosphatase inhibitors were added freshly to all buffers immediately before use. Striatal cells were harvested by trypsinization, washed with ice-cold PBS and counted. For each cell line, approximately 20 million cells were used for fractionation. Cell pellets were resuspended at a density of 5 x 10^6 cells in 500 µL of ice-cold hypotonic buffer [20 mM Tris-HCl (pH 7.4), 10 mM KCl, 2 mM $MgCl_2$, 1 mM EGTA, 0.5 mM DTT, protease inhibitors (cOmplete Protease Inhibitor Cocktail, Roche), and phosphatase inhibitors (PhosSTOP, Roche)] by gentle pipetting up and down. After incubation on ice for 5 min, NP-40 was added to a final concentration of 0.3%. The suspension was mixed by gentle pipetting up and down and incubated on ice for an additional 3 min. An aliquot of cell lysate was retained for subsequent analysis as the total-cell lysate fraction. The remaining lysate was centrifuged at 1000 x g for 5 min at 4°C to separate the nuclear pellet from the cytoplasmic supernatant. To isolate the cytoplasmic fraction, the cytoplasmic supernatant was cleared of remaining cellular debris by centrifugation at 15,000 x g for 5 min at 4°C. The resulting supernatant was collected as the cytoplasmic fraction. The nuclear pellet was washed by resuspension in 800 µL ice-cold isotonic buffer [20 mM Tris-HCl (pH 7.4), 150 mM KCl, 2 mM $MgCl_2$, 1 mM EGTA, 0.5 mM DTT, protease and phosphatase inhibitors] supplemented with 0.3% NP-40 by gently pipetting the suspension up and down. Following a 5 min incubation on ice, the nuclei were pelleted at 1000 x g for 3 min at 4°C. For lysis, the isolated nuclei were resuspended in 100 µL ice-cold, SDS-free RIPA buffer [25 mM Tris-HCl (pH 7.4), 150 mM NaCl, 2 mM $MgCl_2$, 0.5% sodium deoxycholate, 1% NP-40, protease and phosphatase inhibitors] and mechanically disrupted by passing the solution sequentially through 20G and 22G cannulas. Genomic DNA was digested by incubating the lysate with Benzonase (Sigma-Aldrich) for 20 min on ice. Finally, SDS was added to a final concentration of 0.1%, followed by a 10 min incubation on ice. The total cell lysate aliquot was processed similarly by adjusting it to 0.5% sodium deoxycholate and 2 mM $MgCl_2$, followed by cannula shear-disruption and Benzonase digestion for 30 min on ice. Protein concentrations for the total, cytoplasmic, and nuclear fractions were determined using a BCA assay (Thermo Fisher Scientific). For Western blot analysis, samples were denatured in LDS sample buffer containing 50 mM DTT at 70°C for 10 min. Samples were subjected to SDS-PAGE using NuPAGE 4-12% Bis-Tris gels followed by transfer of proteins onto a nitrocellulose membrane using a wet blotting system. Membranes were blocked and washed as described in the Western blot section. Immunoblotting was performed using rabbit anti-Histone H3 (Abcam, ab1791, 1:10000), rabbit anti-GAPDH (Millipore, ABS16, 1:1000), mouse anti-HTT (Millipore, MAB2166, 1:500), rabbit anti-TFE3 (PTG, 14480-1-AP, 1:500), rabbit anti-MITF (PTG,13092-1-AP, 1:500), and rabbit anti-TFEB (PTG, 13372-1-AP, 1:1000) antibodies in 3% milk/PBS, followed by labelling with secondary peroxidase-conjugated anti-mouse or anti-rabbit antibody (1:2000) for one hour.

Golgi Enrichment

Striatal cells were grown in T175 flasks in complete DMEM medium. Approximately 26 million viable cells were used. Cells were washed twice with ice-cold PBS and organelles isolated using the Minute Golgi Apparatus Enrichment Kit according to the manufacturer's instructions (Invent Biotechnologies Inc, Cat #: GO-037). Pellets containing the respective organelle fractions were resuspended in 75 µL PBS supplemented with 1x protease inhibitor. Protein concentrations were determined using the Pierce BCA Protein Assay Kit. The following amounts of protein were used: 30 µg (Input), 10 µg (Mitochondrial-ER-plasma-membrane), 7 µg (Golgi). Samples were subjected to SDS-PAGE using NuPAGE 4-12% Bis-Tris gels followed by transfer of proteins onto a nitrocellulose membrane using a wet blotting system. Membranes were blocked and washed as described in the Western blot section. Immunoblotting was performed using rabbit anti-Histone H3 (Abcam, ab1791, 1:10000), rabbit anti-CLINT1 (Abcam, 86046, 1:2000), rabbit anti-GAPDH (Millipore, ABS16, 1:1000), rabbit anti-GOLGA2 (PTG, 11308-1-AP, 1:5000), rabbit anti-GRASP55 (PTG, 10598-1-AP, 1:1000), rabbit anti-HSP60 (PTG, 15282, 1:1000), rabbit anti-ERp72 (Cell Signaling, 5033, 1:1000), rabbit anti-GOLGIN97 (Cell Signaling, 13192, 1:1000), rabbit anti-HTT (Cell Signaling, D7F7, 1:1000), rabbit anti-HAP40 (Atlas Antibodies, HPA046960, 1:500), and rat

anti-LAMP1 (Santa Cruz, sc-19992, 1:1000) antibodies in 3% milk/PBS, followed by labelling with secondary peroxidase-conjugated anti-mouse or anti-rabbit antibody (1:2000) for one hour.

Plasmids

pDEST26-cmyc-HTTQ23 (full-length) and pDEST26-cmyc-HTTQ145 (full-length) were subcloned from pDONR221-HTTQ23 (human) or pDONR221-HTTQ145 (human) into pDEST26-cmyc-Gateway (GW), a gift from Matthias Selbach. pDEST26-FLAG-HA-Human HAP40 was subcloned from pDONR221-Human HAP40 into pDEST26-FLAG-HA-GW, a gift from Matthias Selbach. pDEST-CMV mCherry-GFP-LC3B WT was a gift from Robin Ketteler (Addgene plasmid #123230) (7). pSpCas9(BB)-2A-Puro-sgRNA Human HTT (3´), pSpCas9(BB)-2A-Puro-sgRNA Human F8A1 (5´), pSpCas9(BB)-2A-Puro-sgRNA#1 Mouse F8A1, and pSpCas9(BB)-2A-Puro-sgRNA#2 Mouse F8A1 were generated by ligating annealed sense and anti-sense oligos into BbsI-digested pSpCas9(BB)-2A-Puro (PX459) V2.0 vector (Addgene plasmid #62988). pMK-Human HTT-mNeonGreen homology-directed repair (HDR) template was designed using Benchling.com software, in which the mNeonGreen gene is flanked (left and right) by 500 base pairs corresponding to the integration site located before the stop codon of the HTT gene. The HDR template was synthesized by gene synthesis and cloned into the pMK backbone (Thermo Fisher Scientific). pDONR221-human HAP40 mutants (M2, M3, M5) were generated via site-directed mutagenesis of the pDONR221-HAP40 (human) plasmid. Plasmids used for BRET assays were generated by shuttling open reading frames in the pDONR221 donor vector into destination vectors (pcDNA3.1-PA-mCit-GW, pcDNA3.1-GW-PA-mCit, pcDNA3.1-NL-GW, pcDNA3.1-GW-NL) as previously described (8). BRET control plasmids (pcDNA3.1-PA-NL, pcDNA3.1-NL, pcDNA3.1-PA-mCit, pcDNA3.1-PA-mCit-NL) were previously generated and described (8). Generation of HTT intramolecular BRET sensor was previously described (9). Protein purification expression constructs for full-length HTT and HAP40 were previously described (10, 11). pFBOH-MHL-HAP40-M2, -M3, and -M5 vectors were generated via site-directed mutagenesis of the pFBOH-MHL-HAP40 vector (Addgene plasmid #124060). The following primers were used for mutagenesis: for the M2 construct, FWD TCCCAAGAAGCTCTTTCTGCTGCTCCAG and REV AAAGAGC-TTCTTGGGAAGCTGGCCGCTGCTC; for the M3 construct, FWD TTCGTCTTCTTT-TTGTGGGTAGCCATGACCAAAG and REV ACAAAAAGAAGACGAAAGCCATCAAGTCGC-TGCAG. All four mutagenic primers (M2_FWD, M2_REV, M3_FWD, M3_REV), were used to generate the M5 construct.

Transfections

For overexpression studies, one million HEK293 cells or 300,000 mouse striatal cells in a final volume of 2 mL complete DMEM medium were reverse transfected in six-well plates with a transfection mix composed of 2 µg of DNA in a volume of 100 µL Opti-MEM medium (Thermo Fisher Scientific, 51985034) and 6 µL FuGENE transfection reagent (1:3 ratio) following manufacturer’s instructions. After 48 hours, cells were dissociated using trypsin, washed with ice-cold PBS, pelleted and stored at -80°C until further processing for Western blotting.

siRNA transfections

Striatal cells were seeded into a 6-well plate at a density of 300,000 cells per well in 2 mL of complete medium. Directly after seeding, siRNA transfection mix was prepared in the following manner. First, ON-TARGETplus mouse *Sec13* siRNA SMARTpool (Horizon Discovery, L-051549-01-0005) and ON-TARGETplus non-targeting control pool (Horizon Discovery, D-001810-10-05) were resuspended in RNase-free 1x siRNA buffer to a final concentration of 10 µM. Then, per well, 9 µL of RNAiMAX reagent (Thermo Fisher, 13778-150) was mixed with 150 µL Opti-MEM medium (Thermo Fisher Scientific, 51985034), and, in separate tubes (target and control), 3 µL of 10 µM siRNA were combined. Next, diluted siRNA was combined with diluted RNAiMAX reagent (1:1 ratio) and incubated for 5 min at room temperature. Afterwards, 300 µL of transfection mix was added dropwise to the respective wells. Cells were incubated with siRNA transfection mix for 48 hours at 33°C. After 48 hours, medium was removed, cells were washed 3x with warm PBS, and 1.5 mL of warm Opti-MEM medium was added to each well to commence the secretion experiment (see Protein secretion assay and trichloroacetic acid precipitation section for further details). In total, cells were exposed to siRNA transfection mix for 72 hours.

BRET screening

BRET assays were performed as described (8). Here, human cDNAs for full-length HTTQ23 and HAP40 were shuttled into destination vectors (pcDNA3.1-PA-mCit-GW, pcDNA3.1-GW-PA-mCit, pcDNA3.1-NL-GW, pcDNA3.1-GW-NL) using LR clonase technology according to manufacturer's instructions (Thermo Fisher Scientific). HEK293 cells were reverse co-transfected with NanoLuc and mCitrine fusion constructs using linear polyethyleneimine (25 kDa, Polysciences, 23966) and incubated for 48 hours. Previously generated control vectors expressing only NanoLuc (pcDNA3.1-NL) or PA-mCitrine (pcDNA3.1-PA-mCit) were used as background controls. Additional readout controls included transfecting pcDNA3.1-PA-mCit-NL (tandem control), pcDNA3.1-PA-mCit + pcDNA3.1-NL (tag control), and pcDNA3.1-PA-NL (bleed-through control). Live cell BRET measurements were carried out in flat-bottom white 96-well plates (Greiner, 655983) with each tested interaction pair in triplicate. Infinite microplate readers M1000 or M1000Pro (Tecan) were used for the readout with the following settings: total luminescence, fluorescence of mCitrine recorded at Ex 500 nm/Em 530 nm, luminescence measured using blue (370–480 nm) and green (520–570 nm) band pass filters with 1,000 ms integration time. BRET ratios were calculated by dividing the background-corrected intensity at 520–570 nm by the intensity obtained at 370–480 nm and subsequent donor bleed-through subtraction from NanoLuc only expressing wells (pcDNA-PA-NL), as previously described (8). Threshold for significance was a BRET ratio that was above the BRET ratio of both control interaction pairs.

Intramolecular BRET assay

Screens were performed in 96-well microtiter plates at a density of 40,000 HEK293 cells per well. Cells were reverse co-transfected in duplicates using linear polyethylenimine with HTT intramolecular BRET sensor (150 ng, pcDNA-NL-HTT$^{Q23(2686\text{-}mCit)}$) and increasing amounts of pcDNA-cmyc-HAP40^{WT} (0.08, 0.16, 0.31, 0.63, 1.25, 2.5, 5, 10 ng) or HAP40^{M5} plasmid (0.16, 0.31, 0.63, 1.25, 2.5, 5, 10, 20 ng). After 48 hours, mCitrine fluorescence (Ex/Em: 500 nm/530 nm; gain 80), total luminescence, and BRET (BLUE, 370–480 nm; GREEN 520–570 nm; both filters at 1,000 ms integration time) was assessed using the Infinite microplate reader M1000 (Tecan). BRET ratios were calculated by dividing the background-corrected intensity at 520–570 nm by the intensity obtained at 370–480 nm and subsequent donor bleed-through subtraction from NanoLuc only expressing wells (pcDNA-PA-NL), as previously described (8). Statistical significance was determined via one-way ANOVA relative to HTT intramolecular BRET sensor alone.

Protein expression and purification

Sf9 insect cells were used for HTT expression as previously described (10, 11). Briefly, P3 recombinant baculovirus was used to infect cells, after which point cells were grown at 37°C until 80-85% viability, typically within 72 hours post-infection. For HTTQ23-HAP40, a 1:1 HTTQ23-HAP40 baculovirus ratio was used for infection. Following growth of baculovirus infected Sf9 cells, the cells were harvested by centrifugation, lysed by two freeze-thaw cycles, and insoluble debris was pelleted by centrifugation at 29,416 x g for one hour. Soluble protein was purified from the supernatant by FLAG affinity chromatography, after which point the crude elution was pooled, concentrated, and polished by size-exclusion chromatography on a Superose 6 Increase 10/300 GL column (Cytiva Life Sciences) in gel filtration buffer (20 mM HEPES pH 7.4, 300 mM NaCl, 2.5% (v/v) glycerol, 1 mM TCEP). Purity of eluted fractions was assessed by SDS-PAGE, and fractions containing pure protein were pooled, concentrated, flash-frozen in liquid N_2, after which point aliquots were stored at -80°C until further use.

Differential Scanning Fluorimetry

A Roche Applied Science Light Cycler 480 II was used for all differential scanning fluorimetry experiments. Protein samples were diluted in DSF buffer (20 mM HEPES pH 7.4, 150 mM NaCl, 1 mM TCEP, 5x Sypro Orange) to a final concentration of 100 µg/mL. 20 µL samples were plated in Roche LightCycler 384-well plates and protein unfolding was assessed by fluorescence measurement using 465 nm excitation and 580 nm emission filters over a 20-95°C temperature gradient (0.02°C/s ramp rate). Reactions were performed in triplicate. The temperature at which

the first derivative of the melting curve reached a maximum was considered the melting temperature (Tm) for each protein complex studied.

Proteomics Sample Preparation and Data Analysis

HEK Immunoprecipitation-MS (IP-MS): Sample preparation was conducted using on-bead tryptic digestion, adhering closely to the protocol established (12). Proteins immunoprecipitated from HEK293 cells were subjected to on-bead digestion and analyzed by label-free data-dependent acquisition (DDA) mass spectrometry. Beads were resuspended in 80 µL of digestion buffer (2 M urea, 50 mM Tris, pH 7.5) supplemented with 1 mM dithiothreitol (DTT) and 5 µg/mL sequencing-grade trypsin. The suspension was incubated for one hour at 25 °C with shaking at 1,000 rpm. The supernatant was collected, and the beads were washed twice with 60 µL of the same urea/Tris buffer. Supernatants and washes were combined (~200 µL total), centrifuged at 5,000 x g, and transferred to a clean tube. Reduction was completed by adding DTT to a final concentration of 4 mM and incubating for 30 min at 25°C, 1,000 rpm. Alkylation was performed with 10 mM iodoacetamide (IAA) for 45 min at 25°C in the dark. An additional 0.5 µg of trypsin was added, and samples were digested overnight at 25°C with shaking at 700 rpm. Peptide clean-up was performed using in-house StageTips prepared with two C18 discs (13). Digests were acidified to pH < 3 with 1% formic acid. StageTips were conditioned with 100 µL of 90% acetonitrile / 0.1% formic acid (FA), then equilibrated with two washes of 100 µL 3% acetonitrile / 0.1% FA. Acidified samples were loaded, washed twice with 100 µL 3% acetonitrile / 0.1% FA, and eluted with 50 µL of 50% acetonitrile / 0.1% FA. All centrifugation steps were performed at 3,000 x g using benchtop spin-column adapters. Peptides were separated by nanoLC and analyzed on an Orbitrap Exploris 480 mass spectrometer (Thermo Fisher Scientific) coupled to a Vanquish Neo UHPLC system operated in nano-flow mode using a two-hour gradient. Data were processed using MaxQuant v1.6.3.4 with iBAQ quantification and match-between-runs enabled. Searches were conducted against the Homo sapiens UniProt database (release 2018-07, including isoforms) supplemented with a custom HTT reference sequence (HTT.fasta, 21Q isoform). Protein groups identified as reverse hits, contaminants, or those identified solely by a modified peptide were excluded. Quantification was based on iBAQ values, with log2-transformation applied. Proteins were retained for downstream analysis if identified by at least two peptides and if at least one experimental group contained ≥4 valid values. Resulting data were median centered prior to statistical comparison.

HEK Total Proteome (TMT): HEK293 cell lysates were processed for Tandem Mass Tag (TMTpro) 16-plex labelling using an in-solution digestion workflow adapted from Mertins et al. (14). Proteins were reduced with 5 mM dithiothreitol (DTT) for one hour at 37°C, followed by alkylation with 10 mM iodoacetamide (IAA) for 45 min at room temperature in the dark. Samples were diluted 1:4 with 50 mM Tris-HCl (pH 8.0) and digested sequentially with Lys-C (1:50, w/w) for two hours at room temperature, then with trypsin (1:50, w/w) overnight at room temperature. Digests were acidified to 1% formic acid (FA; pH ~3), diluted to 1.5 mL with 0.1% FA, and cleared by centrifugation at 15,000 x g for 15 min. The supernatant was desalted using SepPak tC18 500 mg cartridges (Waters). For desalting, cartridges were conditioned with 3 mL acetonitrile (ACN) followed by 3 mL of 50% ACN / 0.1% FA, then equilibrated with four volumes of 3 mL 0.1% trifluoroacetic acid (TFA). Peptides were loaded, washed three times with 3 mL 0.1% TFA and once with 3 mL 1% FA, then eluted with 2x 1.5 mL of 50% ACN / 0.1% FA. The eluate was divided into 10 aliquots (~100 µg each), dried by vacuum centrifugation, and stored at –20 °C. For TMT labelling, 100 µg of desalted peptide was resuspended in 20 µL of 100 mM HEPES (pH 8.0). TMTpro reagents (Thermo Fisher Scientific) were equilibrated to room temperature, reconstituted in anhydrous ACN (0.5 mg per 20 µL), and vortexed for 5 min. For each labelling reaction, 4 µL of TMTpro reagent was added to the peptide solution and incubated for one hour at room temperature. Reactions were quenched with 1 µL of 5% hydroxylamine for 15 min. Labelled peptides were pooled in equimolar amounts, dried by SpeedVac, reconstituted in 1 mL of 3% ACN / 0.1% FA, and desalted using SepPak tC18 200 mg cartridges (Waters). High-pH reversed-phase fractionation was carried out using an UltiMate 3000 HPLC system (Thermo Fisher Scientific), and samples were separated into 24 fractions using a gradient optimized for TMT-labelled peptides. Each fraction was dried and stored prior to LC-MS analysis. Peptides were analyzed on an Orbitrap Exploris 480 mass spectrometer (Thermo Fisher Scientific) coupled to an EASY-nLC 1200 system (Thermo Fisher Scientific) with a two-hour LC gradient. Raw MS data were processed using

MaxQuant v2.0.3.0(15). Spectra were searched against the Homo sapiens UniProt reference proteome (release 2018-07, including isoforms) combined with a database of common proteomics contaminants. Reporter ion intensities were log2-transformed and normalized using median-MAD scaling. Data were filtered to remove reverse hits, common contaminants, and identifications based solely on site-modified peptides. Protein groups were retained if supported by at least two peptides and if complete reporter ion intensities were available for all samples in the TMT plex. Differential abundance analysis for all three MS datasets was performed using limma-assisted moderated t-tests (16) with Benjamini–Hochberg false discovery rate (FDR) correction for multiple testing.

ST Total Proteome (DIA): An amount of 5 million striatal cell samples was lysed in 1x SDC buffer consisting of 1% (w/v) sodium deoxycholate (SDC; Sigma-Aldrich), 10 mM dithiothreitol (DTT; Sigma-Aldrich), 40 mM chloroacetamide (CAA; Sigma-Aldrich), and 100 mM Tris-HCl (pH 8.0). Lysis was carried out at 95°C for 10 min to ensure complete protein denaturation and simultaneous reduction and alkylation. Proteins were enzymatically digested overnight at 37°C using endopeptidase Lys-C (Wako) and sequencing-grade trypsin (Promega) at a protein-to-enzyme ratio of 50:1. Peptide clean-up was performed using the AssayMAP Bravo automated liquid handling platform (Agilent Technologies). Peptides were analyzed using a label-free data-independent acquisition (DIA) workflow on an Orbitrap Exploris 480 mass spectrometer (Thermo Fisher Scientific) coupled to a Vanquish Neo UHPLC system (Thermo Fisher Scientific) operating in nano-flow mode with a two-hour reversed-phase LC gradient. Raw data were processed using DIA-NN v1.9 (17), with searches conducted against the Mus musculus UniProt reference proteome (release 2022-03, including isoforms) supplemented with a custom database containing HTT and HAP40 sequences (mouse_HTT_HAP40.fasta). Protein-level identification thresholds were set at Protein.Q.value ≤ 0.01, Lib.Q.Value ≤ 0.01, and Lib.PG.Q.Value ≤ 0.01. Quantification was performed by aggregating precursor intensities using maximum selection across each Run, Protein Group, and Gene. Protein intensities were log2-transformed and quantified using the MaxLFQ algorithm (18). Proteins were retained for further analysis if supported by at least two peptides and if log2-transformed intensities were present in at least 75% of all samples. Missing values were imputed column-wise using a left-censored normal distribution with a randomized Gaussian downshift.

Striatal cells Immunoprecipitation-MS (IP-MS). Following immunoprecipitation (1 mg input), on-bead digestion of the samples was performed in 6M urea (100 mM ammonium bicarbonate, pH 8.0) for 2 hours at 37°C with 500 ng of Lys-C in the presence of 5 mM tris(2-carboxyethyl)phosphine (TCEP; Sigma-Aldrich) and 20 mM CAA. Samples were subsequently diluted to 1.6 M urea and further digested with 1000 ng of trypsin overnight at 37°C. The supernatant was quenched with TFA (1%) and the peptides were cleaned using C18 columns (20 mg HNS S18V; the Nest Group) using standard techniques. Peptides were measured using a DIA workflow on an Orbitrap Exploris 480 mass spectrometer coupled to a Vanquish Neo UHPLC system operating in nano-flow mode utilizing a 60 min non-linear gradient. Data analysis was performed using Spectronaut 20 (Biognosys, 20.2.250922.92449) (19) against the canonical and reviewed Mus musculus database (release 2026-03; 17,246 entries) with default settings and cross-run normalization and imputation enabled. The data was also exported without imputation to filter for proteins that were only found in all biological replicates in at least one experimental group. Differential abundance analysis was performed as described above. For all MS datasets, differential abundance analysis was performed using limma-assisted moderated t-tests(16) with Benjamini–Hochberg false discovery rate (FDR) correction for multiple testing.

RNA extraction

For quantitative real-time PCR and RNA-sequencing, RNA was purified from mouse striatal cells (70% sub-confluent T175 flask) using a RNeasy kit (Qiagen). DNA contamination was removed from purified RNA by using the RNase-Free DNase set (Qiagen). RNA concentration was determined by measuring the absorbance at 260 nm, and purity was assessed via the 260/280 nm ratio.

Reverse Transcription and Quantitative Real-Time PCR

Quantitative real-time PCR was adapted as previously described (20). Single-stranded cDNA from total RNA was synthesized using the High-Capacity cDNA Reverse Transcription kit (Thermo

Fisher Scientific, 4374967). For qRT-PCR, 50 ng of cDNA per well was amplified in a 10 µL reaction containing TaqMan Gene Expression Master Mix (Thermo Fisher Scientific) and a TaqMan probe specific for mouse *Htt* (Assay ID Mm01213820_m1; primers span exons 64-65). For normalization of data, an endogenous mouse *Actb* (Mm02619580_g1, Thermo Fisher Scientific) FAM/MGB labelled probe was used. Real-time PCR was performed using the ViiA 7 real-time PCR system (Applied Biosystems). Samples were measured in triplicates. Quantification was performed using the ΔCt method.

RNA Sequencing

Total RNA samples were quantified using a Qubit Fluorometer, and RNA integrity was checked on a TapeStation (Agilent Technologies). Double-indexed stranded mRNA-Seq libraries were prepared using the Illumina Stranded mRNA Prep kit (50040534), starting from 500 ng of input material according to the manufacturer's instructions. Libraries were equimolarly pooled based on Qubit concentration measurements and TapeStation size distributions. The loading concentration of the pool was determined using a qPCR assay (Roche, 7960573001). Libraries were then sequenced on the Illumina NovaSeq X Plus platform using PE100 sequencing mode, with a target of 50 million reads per library.

RNA-Seq Data Processing and Differential Expression Analysis

RNA-Seq reads were aligned to the mouse genome (GRCm38, Ensembl release 87, version M12) using STAR (21) (v2.7.3a) with the following parameters: outSAMunmapped Within, outFilterType BySJout, outFilterMultimapNmax 20, alignSJoverhangMin 8, alignSJDBoverhangMin 1, outFilterMismatchNmax 999, outFilterMismatchNoverLmax 0.04, alignIntronMin 20, alignIntronMax 1000000, alignMatesGapMax 1000000. Read assignment to genes was performed with FeatureCounts (22) (v2.0.0) using -T 2 -t exon -g gene_id -s 2 -p. Differential expression analysis was conducted with DESeq2 (23) (v1.38.0), excluding genes with fewer than five counts in at least three samples (**Datasets S4 and S5**).

Principal Component Analysis (PCA)

Principal component analysis (PCA) was conducted in R (v4.5.0) using the prcomp function from the stats package to reduce the dimensionality of the dataset and identify the major axes of variation. Prior to PCA, expression values were log-transformed and scaled to have zero mean and unit variance across features. PCA plots were generated by visualizing the first two principal components, with samples colored by experimental group. For RNA-Seq expression profiles, PCA was applied to mouse striatal cell lines expressing either wild-type HTT (ST*Hdh*^{Q7}) or mutant HTT (ST*Hdh*^{Q111}), each with and without *Hap40* knockout.

Gene Ontology (GO) and KEGG enrichment analyses

Gene Ontology enrichment analyses were performed in R (v4.5.0) using the enrichGO function from the clusterProfiler package (24) (v4.16.0) to identify overrepresented terms. Statistical significance was assessed using a hypergeometric test, and p-values were adjusted for multiple testing with the Benjamini-Hochberg false discovery rate (FDR) method. Terms with FDR ≤ 0.05 were considered significant. Proteins significantly enriched with HTT^{Q23}-HAP40 complexes in WT cells or with apo-HTT^{Q23} in HAP40 KO cells, excluding those also enriched in HTT KO extracts, were analyzed. KEGG pathway enrichment analysis was carried out with shinyGO v0.8 (25) using the KEGG database as reference. Gene identifiers supplied as gene symbols were automatically mapped to Ensembl/STRING IDs via shinyGO's built-in conversion utility. Parameters for analysis included: human background, FDR cutoff of 0.001, redundancy removed, background: all protein-coding genes.

Ingenuity Pathway Analysis (IPA)

Proteomic and transcriptomic datasets were analyzed using Ingenuity Pathway Analysis (26) to identify enriched canonical pathways and biological functions. The significance of each pathway was calculated in IPA using a right-tailed Fisher's exact test, which estimates the probability that the observed association between the dataset and a given pathway occurs by chance. P-values were subsequently adjusted for multiple testing using the Benjamini-Hochberg false discovery rate

(FDR) method. Only the most significant pathways after p-value correction were selected for reporting.

CLEAR and lysosomal omics

For pathway-focused analyses, we used a curated list of genes associated with the Coordinated Lysosomal Expression and Regulation (CLEAR) network, obtained from Ingenuity Pathway Analysis (IPA) Database. In parallel, we used a reference list of lysosome-associated genes and proteins compiled from the literature (27, 28). In Figure 4D, transcriptomic data from ST*Hdh*^{Q7}-*Hap40*KO and ST*Hdh*^{Q111}-*Hap40*KO cells were intersected with the CLEAR gene list to identify significantly upregulated members of this regulatory network. In Figure 4E, differentially expressed genes from the same RNA-Seq analysis were filtered using the lysosomal reference list to highlight transcriptional changes in lysosome-associated genes. In Figure 4F, proteomic datasets from the same cell lines were compared against the lysosomal reference protein list to assess corresponding changes at the protein level.

Figures

Supplementary Figure S1

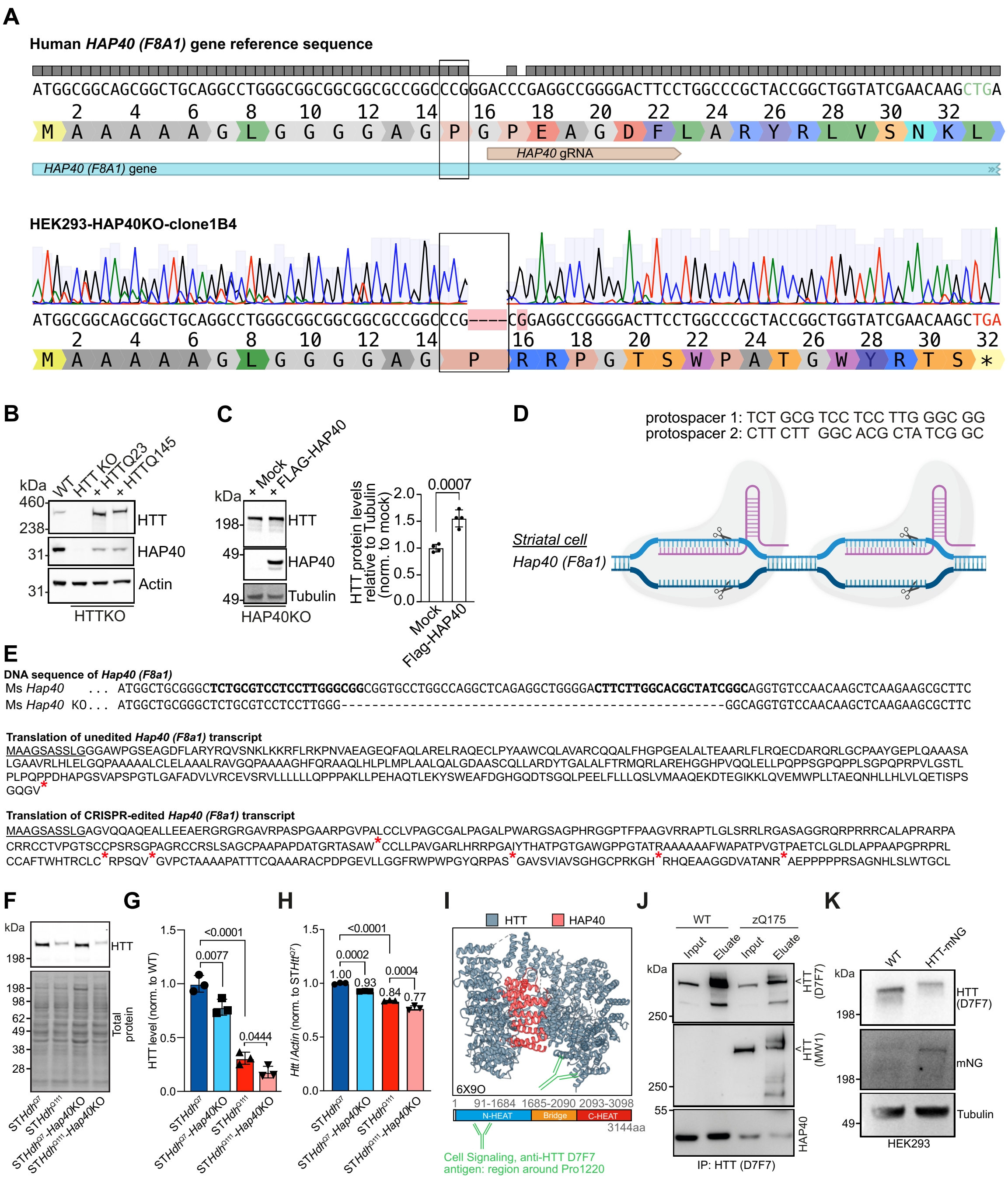

Fig. S1. Characterization of cell lines and mouse brains

A) Sequence chromatogram of HEK293-HAP40KO-clone1B4 showing the CRISPR/Cas9-induced insertion (indel) mutation. The indel causes a frameshift, resulting in a premature stop codon in the *HAP40* (*F8A1*) gene. **B)** Representative immunoblot of HEK293 total cell lysates. HEK293-HTTKO cells were transfected for 48 hours with plasmids for expression of full-length cmyc-HTTQ23 or cmyc-HTTQ145. Samples were immunoblotted using anti-HTT (MAB2166), anti-HAP40, and anti-beta-Actin antibodies. **C)** Left, representative immunoblot of HEK293-HAP40KO total cell lysates. HEK293-HAP40KO cells were transfected for 48 hours with plasmids for expression of FLAG-

HAP40 or mock (FLAG backbone plasmid). Samples were immunoblotted using anti-HTT (D7F7), anti-HAP40, and anti-tubulin antibodies. Right, quantification of the HTT protein levels relative to tubulin. Data are presented as mean ± SD of four biological replicates. Statistical significance was assessed by Student's t-test**. D)** Schematic representation of the CRISPR/Cas9 editing strategy utilizing two sgRNAs to induce a 50-base-pair deletion in the *Hap40* (*F8a1)* gene of mouse striatal cells. Sequence protospacer for each sgRNA is displayed. Created in BioRender. Wanker, E. (2026) https://BioRender.com/cr6sk7g. **E)** Top: DNA sequence alignment of wild-type and CRISPR-edited cells for the mouse *Hap40* (*F8a1)* gene. Dashed lines in the alignment indicate the absence of sequencing reads for the specific bases. Translation of the *Hap40* (*F8a1)* transcript is shown for the wild-type (middle) and CRISPR-edited cells (bottom). Underlined amino acids represent the unaltered amino acid sequence relative to the wild-type sequence and an asterisk denotes the position of a stop codon. **F)** Representative immunoblot of striatal total cell lysates. Membranes were probed with the anti-HTT (MAB2166) antibody. Total protein was visualized using No-Stain protein labelling reagent, which served as a loading control. **G)** Quantification of HTT (MAB2166) protein levels in **F**, expressed relative to total protein and normalized to ST*Hdh*^{Q7}. Data are presented as mean ± SD of three biological replicates. Statistical significance was determined using one-way ANOVA with Holm-Šídák correction for multiple comparisons. **H)** Analysis of steady-state *Htt* transcript levels in mouse striatal cells by qRT-PCR. Specific mouse probes for *Htt* mRNA and *Actin* mRNA were used, *Actin* mRNA was used as a reference gene. Data is expressed as means ± SD, n = 4-5 biological replicates. Statistical significance was determined via one-way ANOVA followed by Tukey's multiple comparisons test. **I)** Top: Cryo-EM structure of HTT-HAP40 (6X9O) illustrating the general location of the anti-HTT (D7F7) target antigen. Bottom: schematic representation of HTT protein sequence, highlighting the N-HEAT, Bridge, and C-HEAT regions, along with the general position of the anti-HTT (D7F7) target antigen in relation to proline 1220. **J)** Representative immunoblot of co-immunoprecipitations of HTT with anti-HTT (D7F7) antibody using mouse brain lysates from 12-month-old wild-type and zQ175 mice. co-IP fractions represent 5% input sample and 100% eluate. Samples were subjected to SDS-PAGE and immunoblotted for HTT using anti-HTT (MAB2166), anti-HTT (D7F7), anti-HTT (MW1, specific for HTT polyQ), and anti-HAP40 antibodies. Three independent experiments were performed. **K)** Representative immunoblot of HEK293 wild-type and HEK293-HTT-mNG knock-in total cell lysates. Membranes were probed with antibodies against anti-HTT (D7F7), anti-mNeonGreen (mNG), and anti-tubulin antibodies.

Supplementary Figure S2

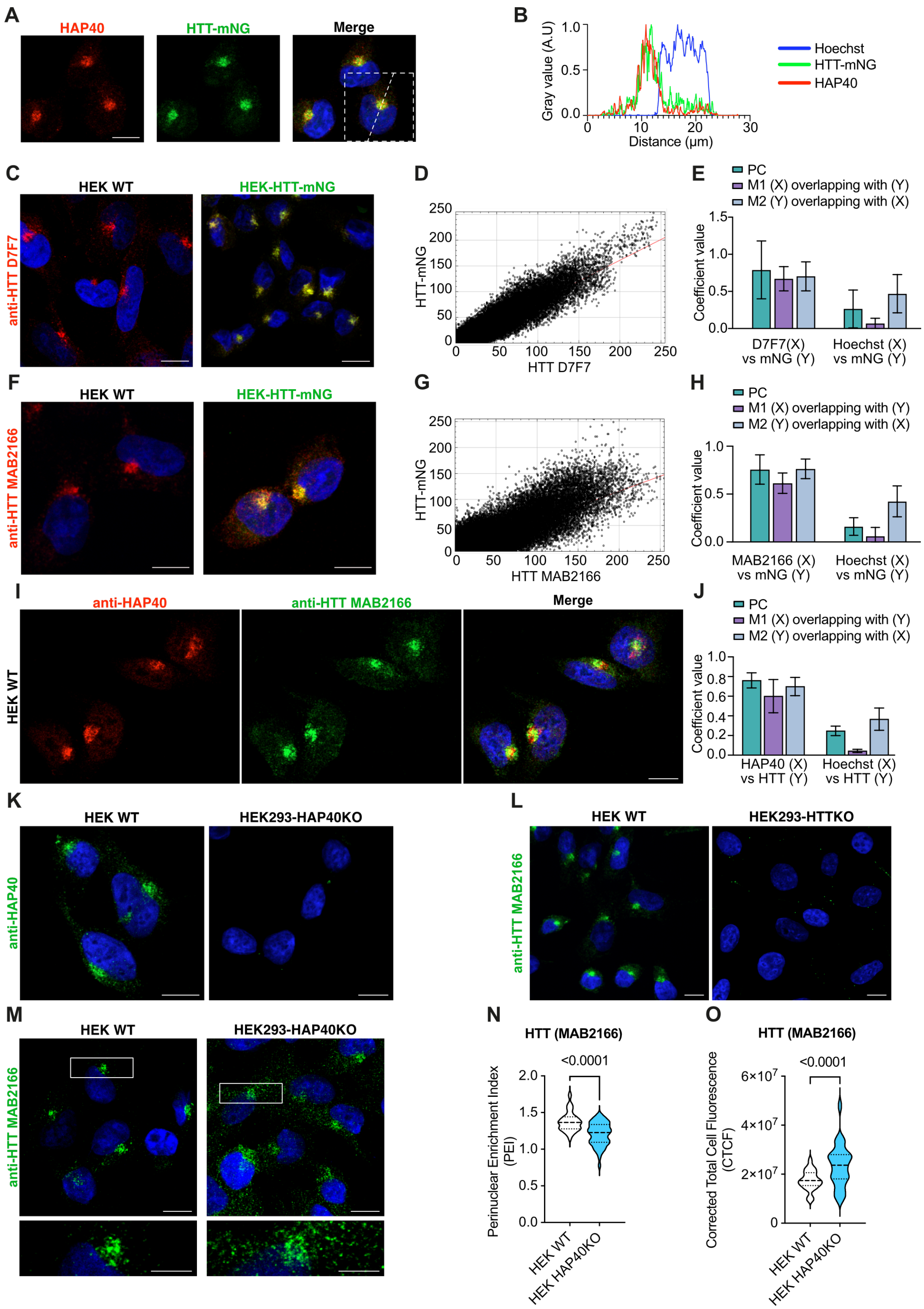

Fig. S2. Control experiments related to HTT-HAP40 confocal imaging

A) Representative confocal images of HEK293-HTT-mNG cells immunostained for HAP40 (red), mNeonGreen (mNG, green), with nuclei counterstained using Hoechst (blue). The merged image includes an inset showing the line used for line-scan analysis. Images were acquired at 63x magnification. Scale bar: 10 µm. **B)** Line-scan analysis of cell shown in **A**, displaying fluorescence intensity (gray values) from all three channels plotted against distance across the line. **C)** Representative confocal images of HEK293 wild-type and HEK293-HTT-mNG cells immunostained for huntingtin (HTT D7F7 antibody, red), mNeonGreen (green) and counterstained with Hoechst for nuclei (blue). **D)** Pixel intensity scatter plot showing the relationship between fluorescence intensities of huntingtin HTT (HTT D7F7 antibody, X-axis) and mNG (Y-axis). Each dot represents the intensity values of a single pixel in the two channels. These data were used to calculate Pearson's correlation coefficients and Manders' coefficients. **E)** Bar graph showing colocalization analysis between anti-HTT (D7F7) (X) and anti-mNG (Y) signals, as well as the colocalization of Hoechst (X) and HTT-mNG (Y) as a negative control. The analysis includes the average Pearson's coefficient (PC) and Manders' M1 (amount of signal from channel X overlapping with channel Y) and M2 (amount of signal from channel Y overlapping with channel X) coefficients. Data represent mean ± SD (n=3). **F)** Representative confocal images of HEK293 wild-type and HEK293-HTT-mNG cells immunostained for huntingtin (HTT MAB2166 antibody, red), mNeonGreen (green) and counterstained with Hoechst for nuclei (blue). **G)** Pixel intensity scatter plot showing the relationship between fluorescence intensities of huntingtin (HTT MAB2166 antibody, X-axis) and mNG (Y-axis). Each dot represents the intensity values of a single pixel in the two channels. These data were used to calculate Pearson's correlation coefficients and Manders' coefficients. **H)** Bar graph showing colocalization analysis assessing anti-HTT (MAB2166) (X) and anti-mNG (Y) signals, as well as the colocalization of Hoechst (X) and HTT-mNG (Y) as a negative control. The analysis includes the average Pearson's coefficient (PC) and Manders' M1 (amount of signal from channel X overlapping with channel Y) and M2 (amount of signal from channel Y overlapping with channel X) coefficients. Data represent mean ± SD (n=3). **I)** Representative confocal images of HEK293 wild-type cells immunostained for huntingtin (HTT MAB2166 antibody, green), HAP40 (red) and counterstained with Hoechst for nuclei (blue). **J)** Bar graph showing colocalization analysis assessing anti-HAP40 (X) and anti-HTT (MAB2166) (Y) signals, as well as the colocalization of Hoechst (X) and anti-HTT (MAB2166) (Y) as a negative control. The analysis includes the average Pearson's coefficient (PC) and Manders' M1 (amount of signal from channel X overlapping with channel Y) and M2 (amount of signal from channel Y overlapping with channel X) coefficients. Data represent mean ± SD (n=3). **K)** Representative confocal images of HEK293-HAP40KO cells immunostained for HAP40 (green) and counterstained with Hoechst for nuclei (blue). **L)** Representative confocal images of HEK293-HTTKO cells immunostained for huntingtin (HTT MAB2166 antibody, green) and counterstained with Hoechst for nuclei (blue). **M)** Representative confocal images of HEK293 wild-type and HEK293-HAP40KO cells immunostained for anti-HTT (MAB2166, green) and counterstained with Hoechst for nuclei (blue). All images were acquired at 63x magnification. Scale bars represent 10 µm for all, except for (**M**) zoom-in image, which represents 5 µm. **N)** Violin plot of perinuclear enrichment index (PEI) that represents the ratio of mean HTT (MAB2166) intensity within a fixed-width perinuclear ring relative to the mean HTT (MAB2166) intensity across the whole cell. A PEI value approaching 1 indicates diffusion of protein (less perinuclear enrichment). **O)** Violin plot of the corrected total cell fluorescence (CTCF) of HTT (MAB2166), which represents the background-corrected HTT abundance per cell. For **N** and **O**, data represent mean ± SD from three independent biological experiments, each with 15-20 cells per genotype, statistical significance was assessed by Mann-Whitney U test.

Supplementary Figure S3

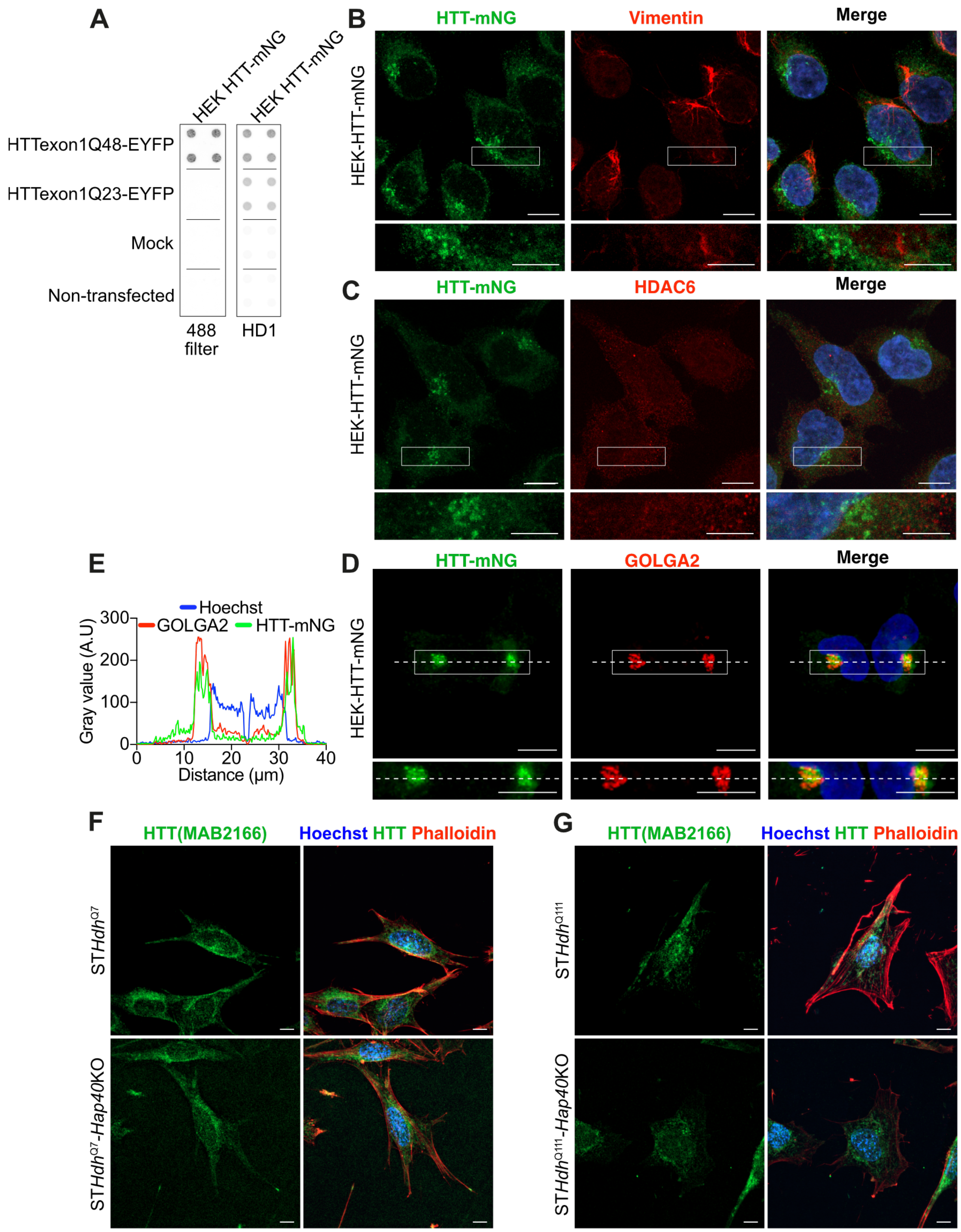

Fig. S3. Assessment of HTT aggregation and subcellular distribution
A) Native filter retardation of protein extracts obtained from HEK293-HTT-mNG cells transfected with HTTexon1Q48-EYFP, HTTexon1Q23-EYFP, pcDNA3.1 (Mock), or non-transfected. Membranes were probed with anti-HD1 to detect wild-type and mutant HTT. Fluorescence EYFP and mNG signals were acquired using the Alexa Fluor Plus 488 filter in the iBright imaging system. Representative confocal images of HEK293-HTT-mNG cells showing endogenous mNeonGreen

(green) signal, counterstained with Hoechst (blue), and immunostaining for **B)** Vimentin, **C)** HDAC6, and **D)** GOLGA2. Box represents the zoom-in region of interest shown below. **E)** Line-scan analysis of dotted line shown in **D**, displaying fluorescence intensity (gray values) from all three channels plotted against distance (μm) across the line. Representative confocal images of **F)** ST*Hdh*^{Q7} and ST*Hdh*^{Q7}-*Hap40*KO cells and **G)** ST*Hdh*^{Q111} and ST*Hdh*^{Q111}-*Hap40*KO cells immunostaining for HTT (MAB2166 antibody), Phalloidin, with Hoechst counterstaining. For all images, scale bars represent 10 μm and 5 μm for zoomed-in images.

Supplementary Figure S4

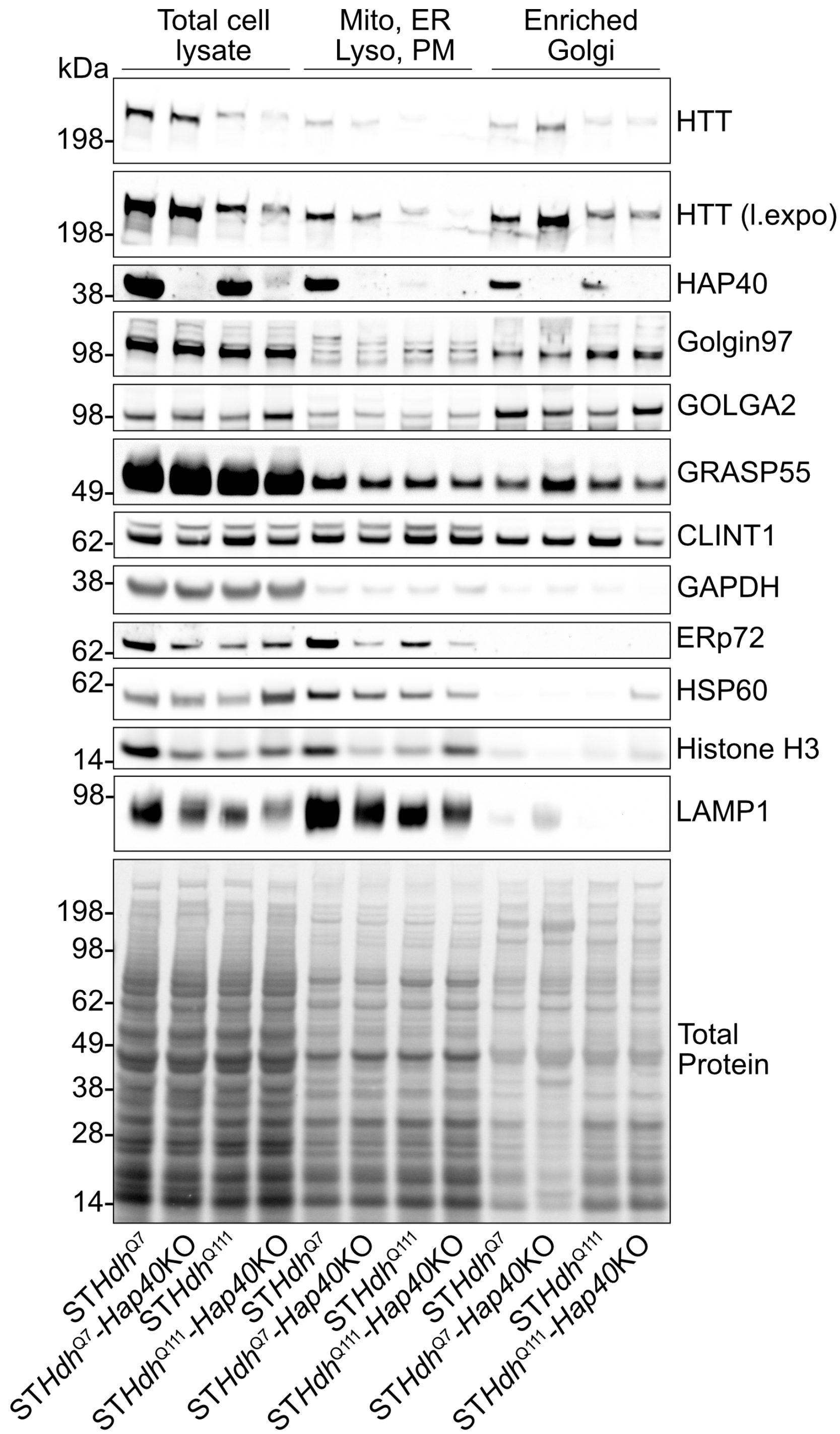

Fig. S4. Subcellular enrichment of HTT and HAP40 in striatal cells
Representative immunoblots of striatal total cell lysates and enriched fractions for mitochondria (Mito), endoplasmic reticulum (ER), lysosomes (Lyso), plasma membranes (PM), and Golgi membranes. Fractions were probed with antibodies against different organelle markers in addition to HTT and HAP40. Longer exposure of HTT (l.expo). Total protein was visualized using No-Stain protein labelling reagent, which served as a loading control.

Supplementary Figure S5

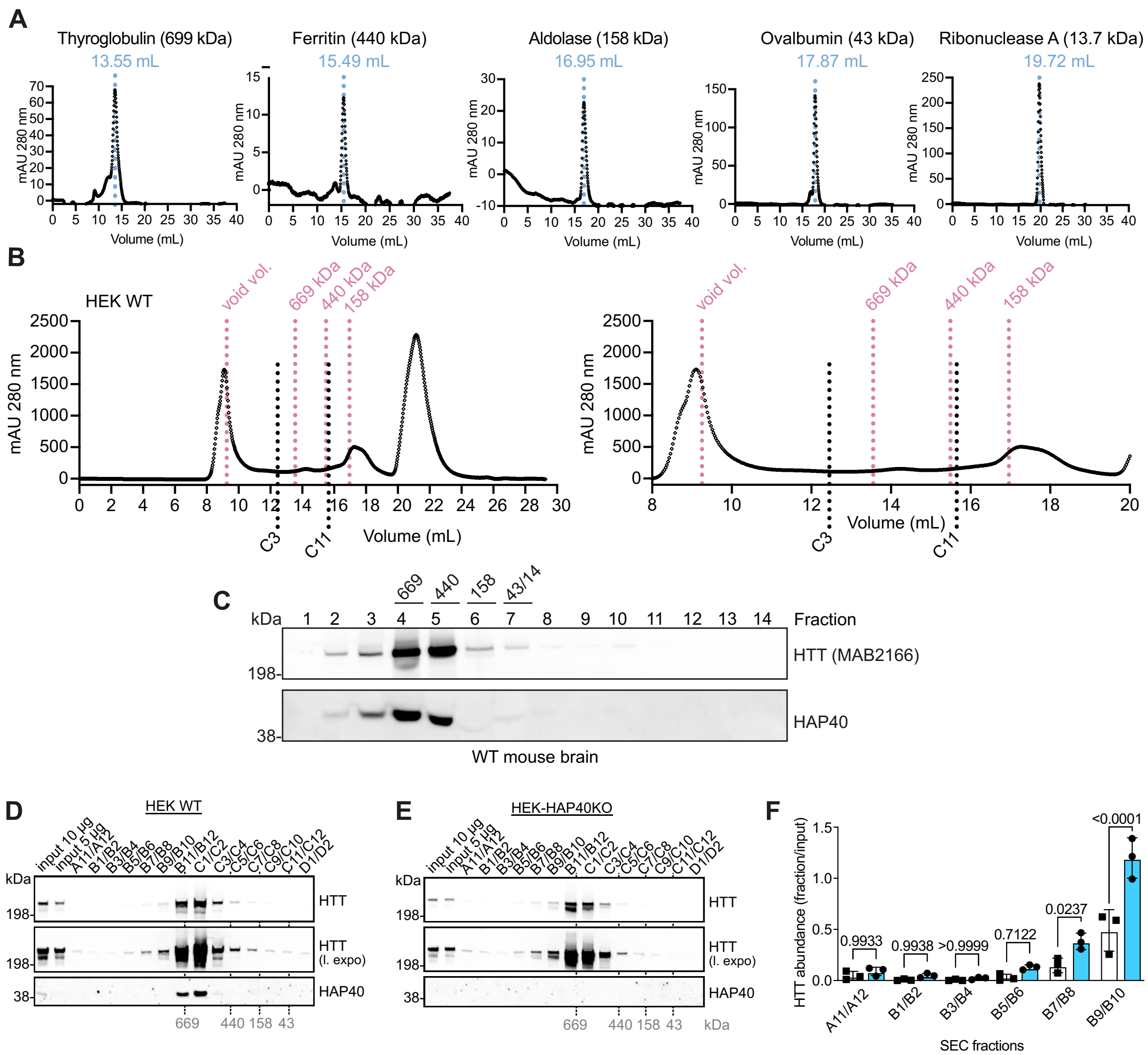

Fig. S5. Size-exclusion chromatography analysis

A) Elution profiles of the molecular weight markers, including Thyroglobulin, Ferritin, Aldolase, Ovalbumin, and Ribonuclease A used for calibration of Superose 6 Increase 10/300 GL column. The x-axis represents the absorption measured at 280 nm, while the y-axis indicates the volume at which the marker protein elutes from the column. The elution volume is also noted above the peaks. **B)** Elution profile of HEK WT lysate analyzed by size-exclusion chromatography (SEC) using a Superose 6 Increase column. The molecular weights of marker proteins and their corresponding elution volume (indicated by the magenta dotted line) are schematically shown at the top of the diagram. At the bottom, fractions C3 and C11 are indicated, eluting at 12.45 mL and 15.65 mL (black dotted line), respectively. A magnified section of the diagram is presented on the right side. **C)** Representative SEC immunoblots of wild-type mouse brain total lysates. Fractions were pooled by combining three wells to obtain a total of 14 final fractions. Proteins were acetone-precipitated, and 50% of each fraction in 1x LDS was subjected to SDS-PAGE followed by immunoblotting using anti-HTT (MAB2166) and anti-HAP40 antibodies. Molecular weight markers are displayed above the fractions. Representative SEC immunoblots of **D)** HEK WT and **E)** HAP40KO total lysates probed with anti-HTT(D7F7) and anti-HAP40 (Atlas) antibodies. Short and long exposures (l.expo) are shown for HTT. **F)** Quantification of HTT protein levels among fractions A11 to B10 relative to input (5 µg). Data are presented as mean ± SD from three biological replicates; statistical significance was assessed by two-way ANOVA with Šídák's multiple comparisons test.

Supplementary Figure S6

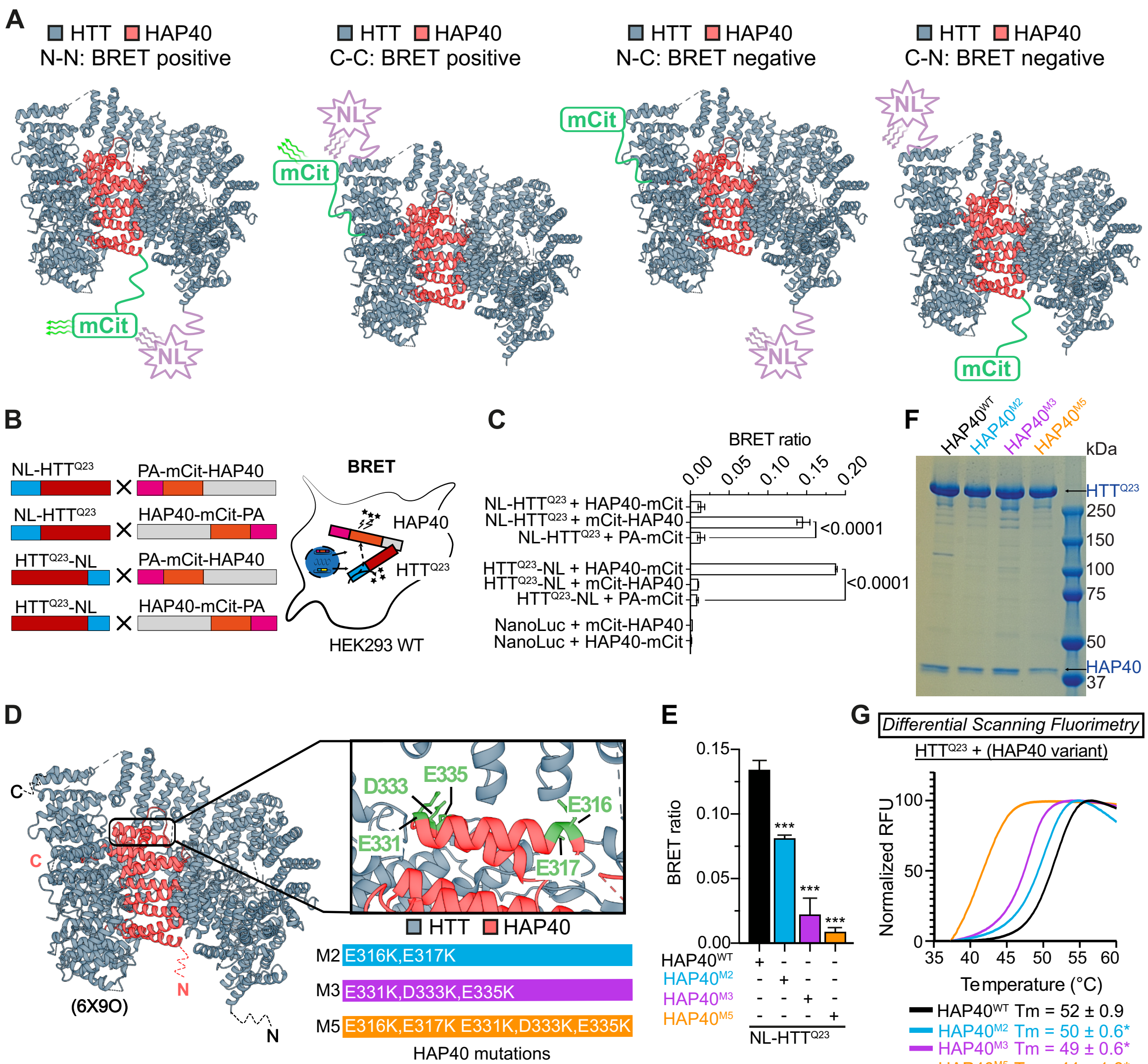

Fig. S6. Detection of HTT-HAP40 interactions by BRET and differential scanning fluorimetry A) Cryo-EM structure of HTT-HAP40 (PDB: 6X9O) illustrating the different tagging orientations and BRET screening combinations. Screening with HTT and HAP40 tagged at the same termini (C–C or N–N) resulted in high BRET ratio values, whereas opposite orientations (N–C or C–N) showed non-significant BRET signals, likely due to the increased distance between donor and acceptor. **B)** Schematic representation of the BRET assay workflow. Expression vectors encoding c-myc-NL and PA-mCit tagged proteins were co-transfected into HEK293 cells. Binary interactions were detected *in vivo* using in-cell bioluminescence resonance energy transfer (BRET). **C)** BRET ratios of binary interactions between NanoLuc- and mCitrine-tagged full-length HTT^{Q23}, HAP40, and respective controls. Data are presented as mean ± SD (n=2), each with three technical replicates. Statistical significance was determined via two-way ANOVA followed by Bonferroni's multiple-comparisons test. **D)** Left: Cryo-EM structure of HTT-HAP40 (6X9O). HAP40 is shown in red and HTT in blue. Right: Zoomed-in view displaying the five conserved HAP40 amino acid residues (green: E316, E317, E331, D333, E335) and the three HAP40 mutant variants generated based on the number of amino acids converted into lysine. **E)** BRET ratios of binary interactions between c-myc-NL-HTT^{Q23} and PA-mCit-HAP40 (wild-type and mutants). Data are presented as mean ± SD, n=2, each with three technical replicates. Statistical significance was determined via two-way ANOVA followed by Dunnett's multiple-comparisons test. **F)** Representative SDS-PAGE of purified

full-length HTT^{Q23} in complex with wild-type HAP40 or the mutant variants $HAP40^{M2,\ M3,\ and\ M5}$. **G)** Differential scanning fluorimetry profiles and calculated melting temperature (Tm) values for full-length HTT^{Q23} and wild-type HAP40 (black), $HAP40^{M2}$ (teal), $HAP40^{M3}$ (magenta), and $HAP40^{M5}$ (orange). Melting temperatures were determined from the inflection point of the curves obtained by fitting the data to the Boltzmann sigmoidal function. Significant (≥2 °C) destabilizations relative to the wild-type HAP40 are indicated with an asterisk. Data are presented as mean ± SD (n=3).

Supplementary Figure S7

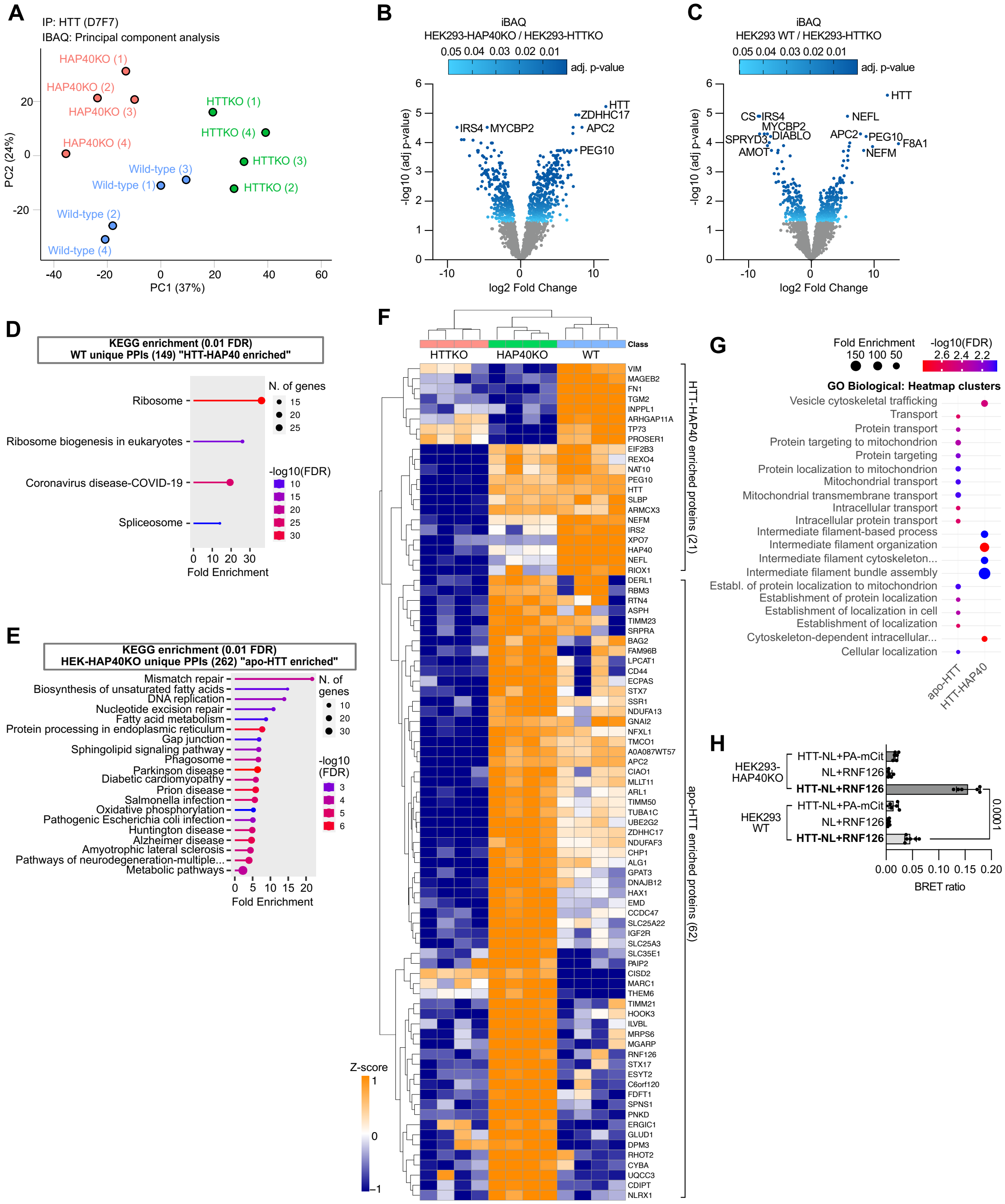

Fig. S7. Detection and analysis of HTT-associated proteins by co-IP-MS and BRET assays
A) Principal component analysis of HEK293 HTT-IP-MS datasets. Points represent individual biological samples, colored by experimental group (wild-type, HTTKO, HAP40KO). The axes display PC1 and PC2, with the proportion of variance explained by each component indicated. **B)** and **C)** Volcano plots of HTT-IP-MS iBAQ data comparing HEK293-HAP40KO vs HEK293-HTTKO and wild-type vs HEK293-HTTKO. The x-axes display $\log_2$ fold changes, the y-axes show $-\log_{10}$ adj. p-values (Benjamini–Hochberg). Shades of blue dots denote significantly enriched proteins (adj. p-value ≤ 0.05); gray dots represent proteins that do not reach significance (adj. p-

value > 0.05). **D)** KEGG pathway enrichment for proteins significantly enriched in wild-type/HEK293-HTTKO and **E)** in HEK293-HAP40KO/HEK293-HTTKO HTT-IP-MS samples. Only pathways with a Benjamini–Hochberg FDR < 0.01 are shown. Plots show pathways ranked by fold enrichment and the number of constituent proteins is indicated by circle size and the -$\log_{10}$ adj. p-value in color scale. **F)** Heatmap showing hierarchical clustering (Euclidean distance) of row-wise Z-scaled iBAQ intensities for proteins meeting the stringent enrichment threshold (FDR ≤ 0.001) across the three HEK293 cell lines. **G)** Gene ontology (Biological Process) enrichment for the HTT-HAP40-enriched and apo-HTT-enriched proteins. Each term is represented by a circle whose diameter indicates the fold-enrichment value, while the color gradient reflects the Benjamini–Hochberg FDR. Only terms with FDR < 0.05 are displayed. **H)** BRET ratios of binary interactions between NanoLuc- and mCitrine-tagged full-length HTT^{Q23} and RNF126 and respective controls. Interactions were tested in HEK293 wild-type and HEK293-HAP40KO cells. Data are presented as mean ± SD, n=2, each with three technical replicates.

Supplementary Figure S8

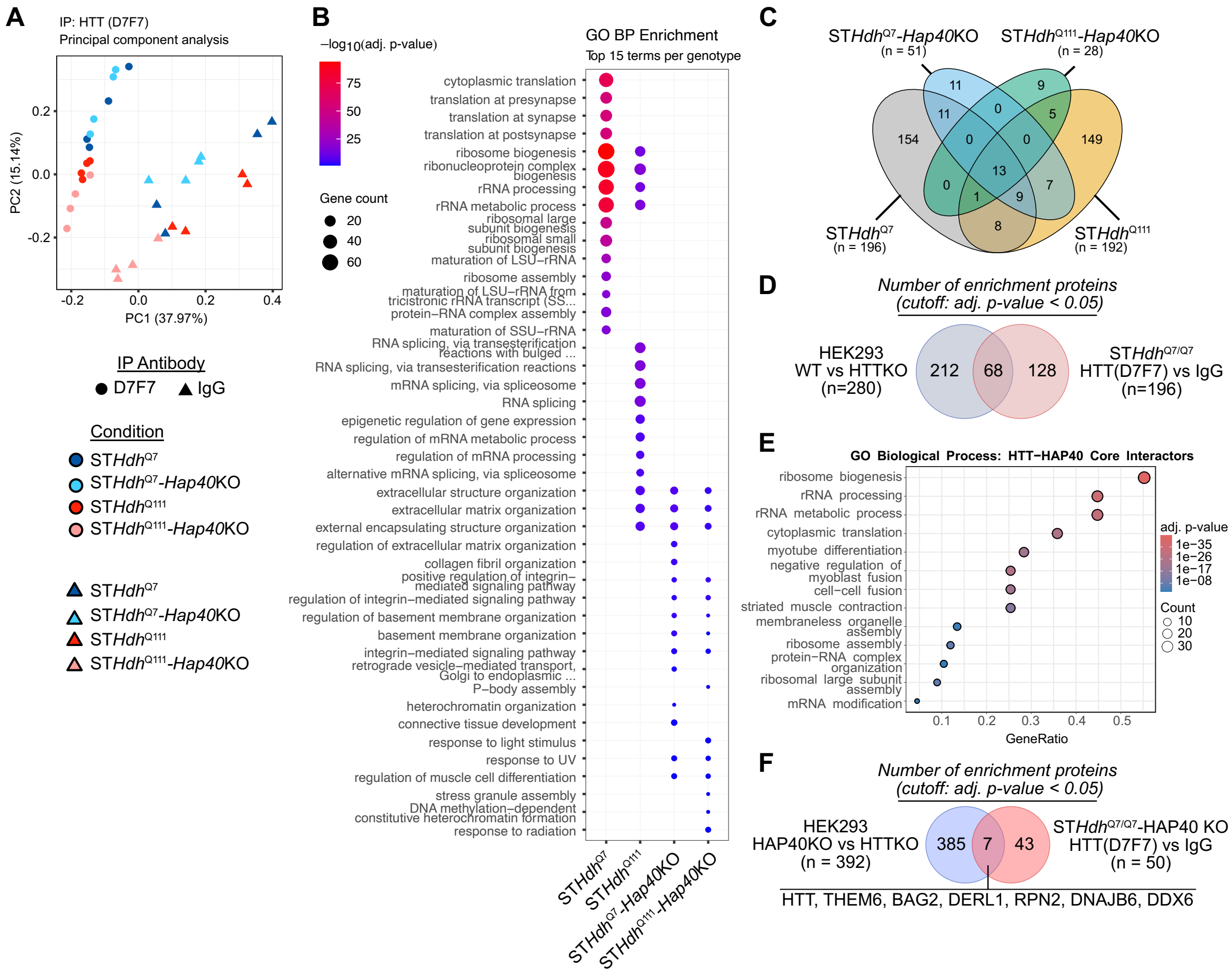

Fig. S8. HTT-IP-MS analysis of striatal cells across wild-type, mutant HTT, and HAP40KO genotypes.

A) Principal Component Analysis of the striatal Co-IP data. The PCA plot visualizes the proteomic variance across 32 samples based on 1,480 proteins identified. The first principal component (PC1) separates specific pull-downs (D7F7, circles) from non-specific controls (IgG, triangles). The second principal component (PC2) reflects differences related to the HTT genotype (Q111 vs. Q7) and HAP40 status. Biological replicates of the D7F7 groups cluster closely, indicating experimental reproducibility for the specific enrichment. In contrast, the IgG control samples show higher dispersion, reflecting the inherent stochastic nature of non-specific background noise. **B)** Gene Ontology enrichment analysis of the HTT interactome. The dot plot displays the top 15 significantly enriched Biological Process terms identified for each genotype. The size of the dots represents the number of proteins (Gene count) associated with each term, providing an estimate of the biological weight of the process. The color intensity represents the statistical significance as -log$_{10}$ (adj. p-value), with higher values (red) indicating stronger enrichment. **C)** Comparison of HTT protein interactors across genotypes. The Venn diagram illustrates the distribution of proteins significantly enriched in D7F7-immunoprecipitates compared to their respective IgG controls. Numbers indicate the count of proteins for each contrast. A core set of 13 proteins was identified across all four groups, representing stable HTT interaction partners independent of polyQ length or HAP40 presence. **D)** Venn diagram shows significantly enriched (adj. p-value < 0.05) proteins identified by HTT-IP-MS that are shared between HEK293 WT and ST*Hdh*^{Q7} cells. **E)** Gene Ontology Biological Process enrichment analysis of the shared 68 core HTT-HAP40 interacting proteins between HEK293 WT and ST*Hdh*^{Q7} cells**. F)** Venn diagram shows significantly enriched (adj. p-value < 0.05) proteins identified by HTT-IP-MS that are shared between HEK293-HAP40KO and ST*Hdh*^{Q7}-*Hap40*KO cells.

Supplementary Figure S9

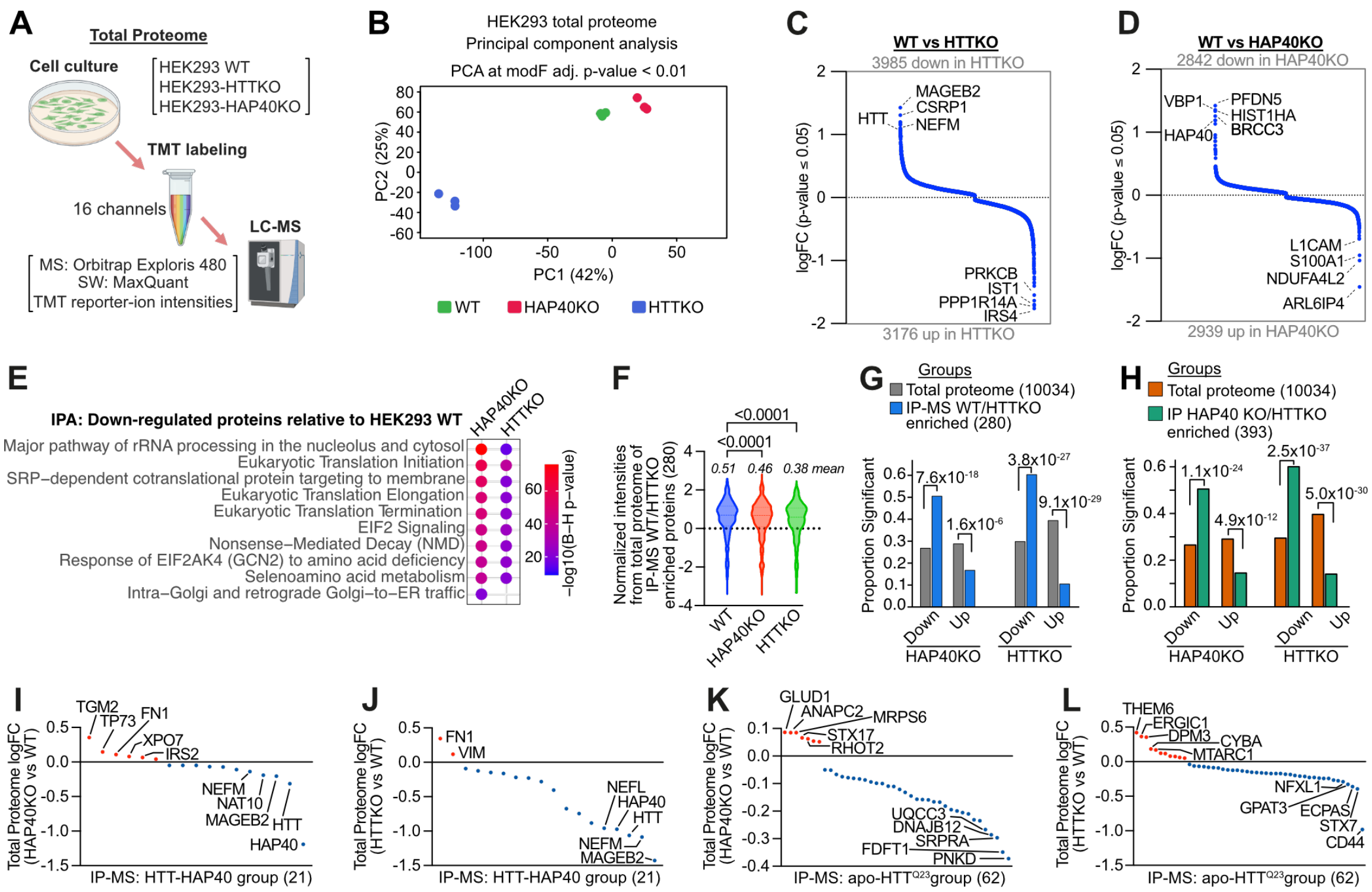

Fig. S9. Proteomic profiling of HTT- and HAP40-dependent protein changes in HEK293 cell lines

A) Overview of the HEK293 total proteome workflow. Three HEK293 cell lines (wild-type, HTTKO, and HAP40KO) were processed for Tandem Mass Tag (TMT-16-plex) labeling and subsequent LC-MS/MS analysis. Four independent biological replicates were performed for each genotype. The graphic was created in BioRender. Wanker, E. (2026) https://BioRender.com/ci9qwne. **B)** Principal component analysis of HEK293 total proteome datasets. Points represent individual biological samples, colored by experimental group (wild-type, HTTKO, HAP40KO). The axes display PC1 and PC2 with the proportion of variance explained by each component indicated. **C)** Scatter plot of protein-level changes between HEK293 wild-type and HTTKO cells (total proteome TMT-MS). The vertical axis shows the log fold change (logFC) of each protein, a significance cutoff of adj. p-value < 0.05 (Benjamini-Hochberg FDR correction) is indicated. Positive values denote higher abundance in wild-type, negative values denote higher abundance in the HTTKO. **D)** Same representation as in **C** but contrasting wild-type with HAP40KO cells. Axes and statistical thresholds are identical. **E)** Ingenuity Pathway Analysis (IPA) of proteins significantly downregulated (adj. p-value < 0.05) in both HAP40KO and HTTKO versus wild-type HEK293 cells. Canonical pathways are ranked by Benjamini-Hochberg FDR-adjusted p-values. **F)** Cross-reference of co-IP-MS hits (280 WT/HTTKO enriched proteins) against the total proteome TMT-MS dataset. Normalized intensities from the three HEK293 cell lines were used for comparison. **G)** Enrichment analysis of differentially regulated proteins. Two groups were examined: (i) the 280 co-IP-MS WT/HTTKO enriched proteins and (ii) the global HEK293 TMT-MS proteome in either HAP40KO or HTTKO cells. For each group, proteins were classified as significant or non-significant (adj. p-value ≤ 0.05) based on the TMT-MS data. Fisher's exact test was applied to assess enrichment; p-values are displayed above the bars. **H)** Enrichment analysis of differentially regulated proteins. Two groups were examined: (i) the 393 IP-MS HAP40KO/HTTKO enriched proteins and (ii) the global HEK293 TMT-MS proteome in either HAP40KO or HTTKO cells. For each group, proteins were classified as significant or non-significant (adj. p-value < 0.05) based on the TMT-MS data. Fisher's exact test was applied to assess enrichment; p-values are displayed above the bars. **I)** LogFC values for the 21 proteins that clustered together in the hierarchical analysis shown in **Fig. S7F** (HTT-HAP40

enriched protein set) plotted against the total proteome TMT-MS data for HAP40KO versus wild-type. **J)** Same 21-protein set as in **I**, now plotted for the HTTKO versus wild-type comparison. All statistical analyses employed Benjamini-Hochberg correction for multiple testing unless otherwise noted. **K)** LogFC values for the 62 proteins that clustered together in the hierarchical analysis shown in **Fig. S7F** (apo-HTT-enriched protein set) plotted against the total-proteome TMT-MS data for HEK293-HAP40KO versus wild-type. **L)** Same 62 protein set as in **K**, now plotted for the HEK293-HTTKO versus wild-type comparison.

Supplementary Figure S10

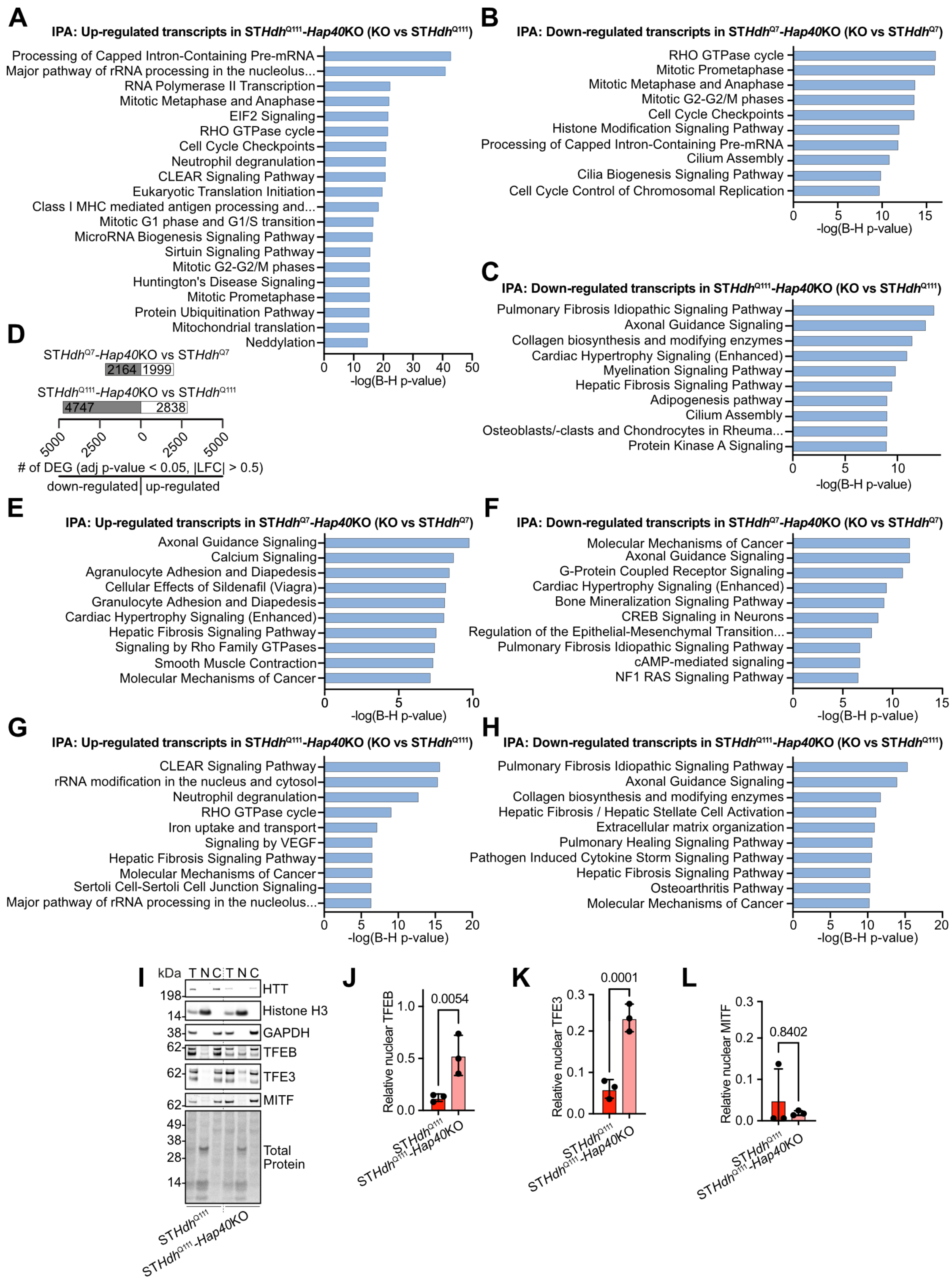

Fig. S10. Analysis of transcriptome datasets of cell lines and subcellular distribution of CLEAR pathway activating transcription factors

A-C) IPA of canonical pathways based on significantly altered transcripts (adj. p-value ≤0.05, no LFC cutoff applied) in *Hap40*KO cells relative to their respective controls: **A)** Upregulated

transcripts in ST*Hdh*^{Q111}-*Hap40*KO vs ST*Hdh*^{Q111} cells. **B)** Downregulated transcripts in ST*Hdh*^{Q7}-*Hap40*KO vs ST*Hdh*^{Q7} cells. **C)** Downregulated transcripts in ST*Hdh*^{Q111}-*Hap40*KO vs ST*Hdh*^{Q111} cells. **D)** Numbers of significantly upregulated (white) and downregulated (gray) transcripts (adj. p-value ≤0.05, |LFC| > 0.5) in the two *Hap40*KO lines relative to their respective parental controls. The total number of differentially expressed genes is shown in each bar. **E-H)** IPA of canonical pathways based on significantly altered transcripts with an additional effect size threshold (adj. p-value ≤ 0.05, |LFC| > 0.5) in *Hap40*KO cells relative to their respective controls: **E)** Upregulated and **F)** downregulated transcripts in ST*Hdh*^{Q7}-*Hap40*KO vs ST*Hdh*^{Q7} cells. **G)** Upregulated and **H)** downregulated transcripts in ST*Hdh*^{Q111}-*Hap40*KO vs ST*Hdh*^{Q111} cells. For **A-C** and **E-H**, pathways were ranked by Benjamini-Hochberg FDR-adjusted p-values. **I–L)** Subcellular fractionation analysis of CLEAR network transcription factors in ST*Hdh*^{Q111} and ST*Hdh*^{Q111}-*Hap40*KO cells under steady-state conditions. **I)** Representative immunoblots showing protein levels of HTT, the nuclear marker Histone H3, the cytoplasmic marker GAPDH, and the CLEAR network transcription factors TFEB, TFE3, and MITF in total lysate (T), nuclear (N), and cytoplasmic (C) fractions. Total protein staining served as a loading control. **J–L)** Densitometric quantification of relative nuclear levels for **J)** TFEB, **K)** TFE3, and **L)** MITF. Data are presented as mean ± SD (n = 3 independent experiments). Statistical significance was determined using an unpaired two-tailed Student's *t*-test (p-values indicated above bars).

Supplementary Figure S11

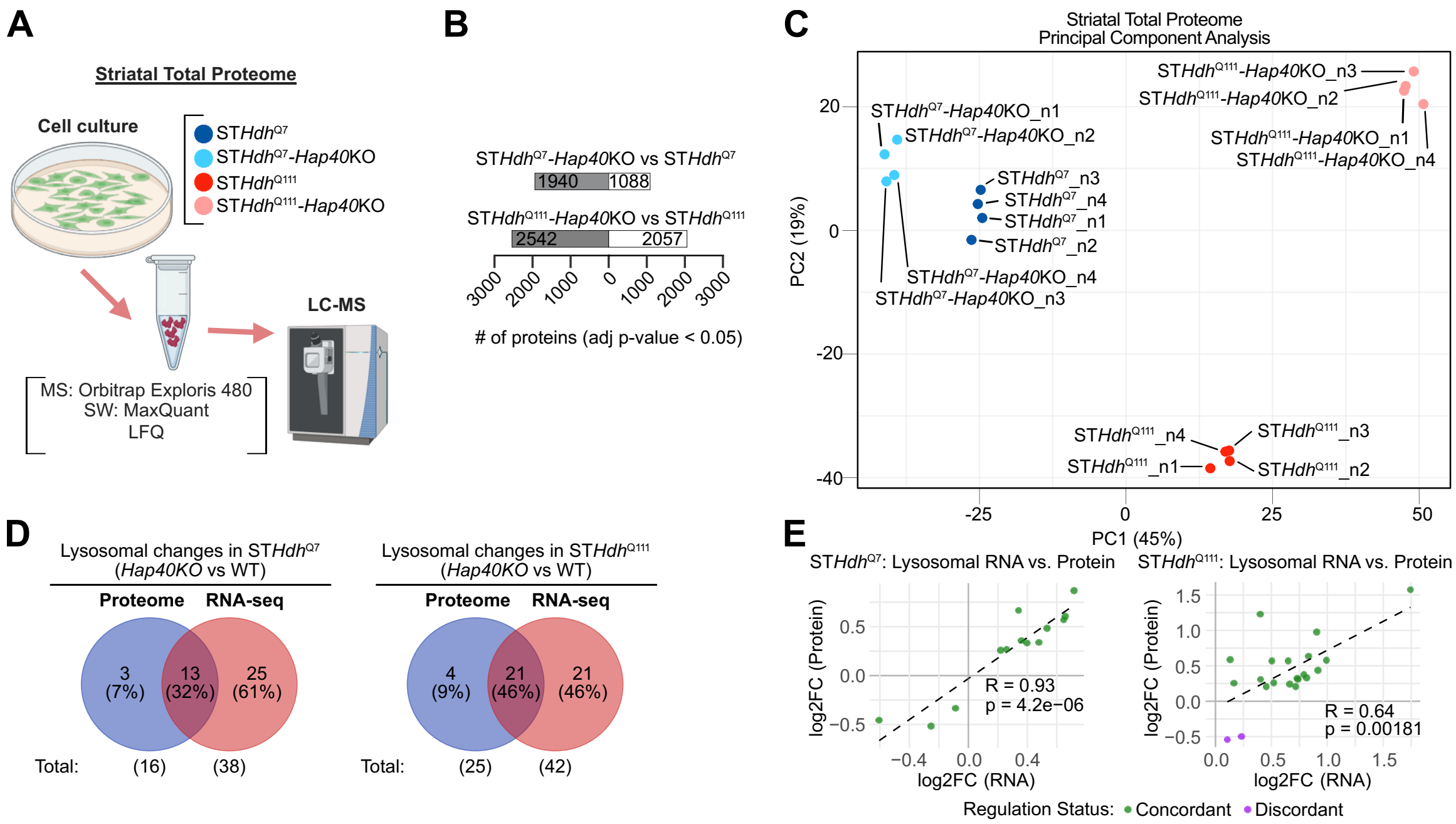

Fig. S11. Analysis of proteome datasets of striatal cell lines and comparison of omics data sets

A) Overview of the mouse striatal total proteome workflow. Four mouse striatal cell lines (ST*Hdh*^{Q7}, ST*Hdh*^{Q7}-*Hap40*KO, ST*Hdh*^{Q111}, ST*Hdh*^{Q111}-*Hap40*KO) were subjected to label-free quantitative (LFQ) LC-MS/MS. Four independent biological replicates were prepared for each genotype. Created in BioRender. Wanker, E. (2026) https://BioRender.com/ci9qwne. **B)** Numbers of significantly upregulated (white) and downregulated (gray) proteins (adj. p-value < 0.05, no LFC cutoff applied) in the total proteome of striatal cells from the two *Hap40*KO lines relative to their respective parental controls. The total number of differentially expressed proteins is shown in each bar. **C)** Principal component analysis of the striatal total proteome dataset. Points represent individual biological samples, colored by experimental group. The axes display PC1 and PC2. **D)** and **E)** Cross-comparison of lysosomal gene and protein expression changes upon *Hap40* loss in ST*Hdh*^{Q7} and ST*Hdh*^{Q111} cells relative to parental controls. **D)** Venn diagrams illustrating the overlap between altered lysosomal proteins (proteome) and transcripts (RNA-seq) for ST*Hdh*^{Q7} (left) and ST*Hdh*^{Q111} (right) cells. Total numbers of identified altered lysosomal proteins and transcripts are indicated in parentheses below. **E)** Correlation plots comparing log2 fold-changes (log2FC) at the transcript (x-axis) and protein level (y-axis) for lysosomal targets in ST*Hdh*^{Q7} (left) and ST*Hdh*^{Q111} (right) cells. Points are color-coded by regulation status (green: concordant; purple: discordant). Pearson's correlation coefficients (R) and corresponding p-values are indicated.

Supplementary Figure S12

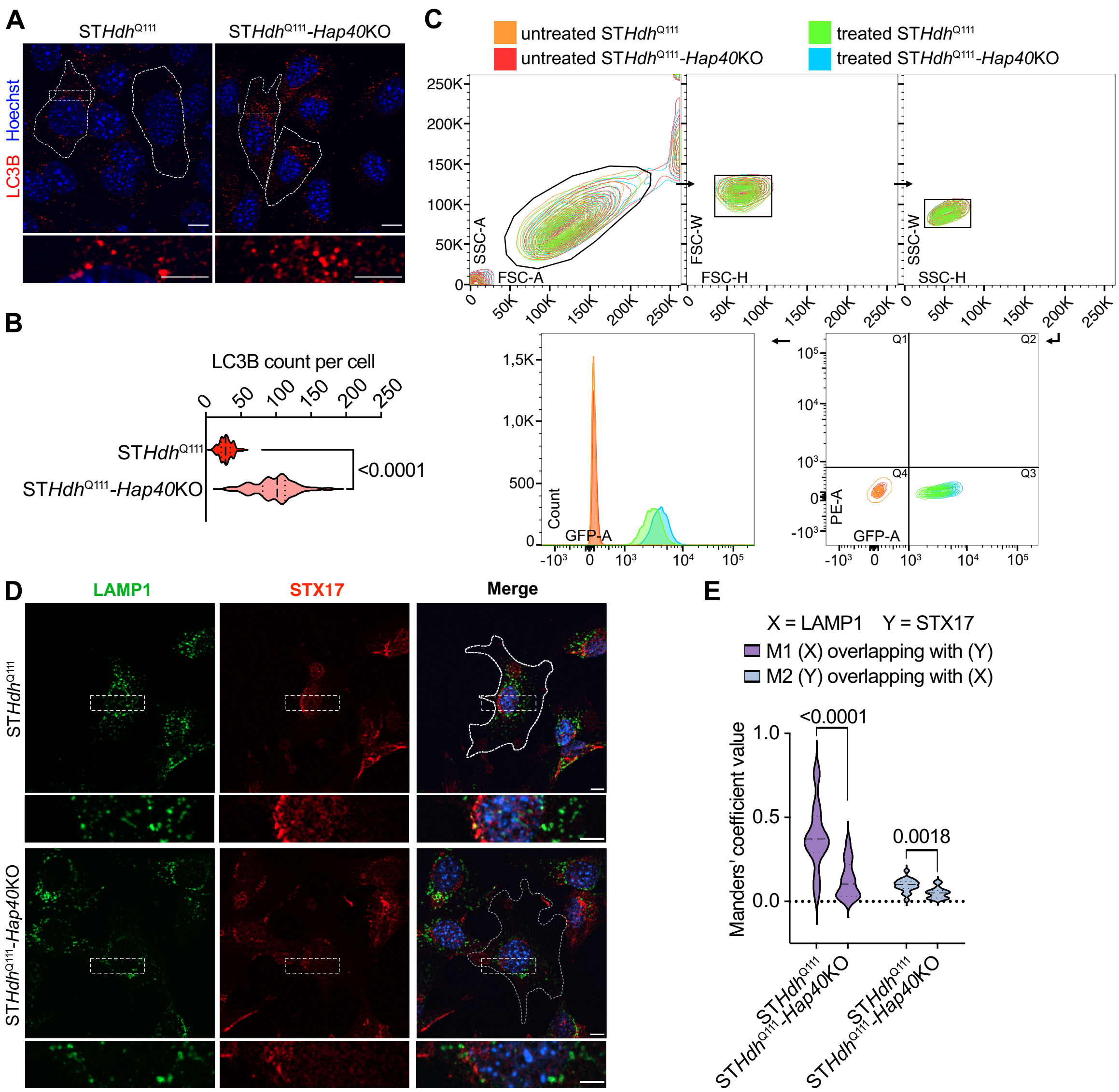

Fig. S12. Assessment of autophagic vesicles across HTT and HAP40 genotypes in striatal cells

A) Representative confocal images of striatal ST*Hdh*^{Q111} and ST*Hdh*^{Q111}-*Hap40*KO cell lines immunostained for LC3B. Box represents the zoomed-in region of interest shown below. Cell outlines define the region of interest used for downstream analysis. **B)** Quantification of LC3B count per cell. Fifteen cells per genotype were analyzed across three independent biological replicates; data are shown as mean ± SD. Statistical significance was assessed by one-way ANOVA with Tukey's multiple comparison test. **C)** Flow cytometry data related to **Fig. 5F**. Representative gating strategy used to assess the mean fluorescence intensity (MFI) of autophagic vacuoles in 10,000 cells per biological replicate. Cells were treated with green autophagic vacuole labelling reagent from the Autophagy Detection Kit. Initial gating used forward scatter versus side scatter (FSC-A vs SSC-A) to isolate the cell population; doublets were excluded by plotting FSC-H vs FSC-W followed by SSC-H vs SSC-W. Untreated cells were used as a negative control to set the gate for the final population of fluorescent cells. Fluorescence was detected using 488 nm laser (GFP-A) and the 561 nm laser (PE-A). **D)** Representative confocal images of striatal ST*Hdh*^{Q111} and ST*Hdh*^{Q111}-*Hap40*KO cell lines immunostained for LAMP1 and STX17. Box represents the

zoomed-in region of interest shown below. Cell outline is shown as an example of the region of interest used for downstream analysis. **E)** Bar graph showing Manders' colocalization coefficients assessing LAMP1 (X) and STX17 (Y) signals. Data represent mean ± SD from two independent biological replicates, each with 16 to 18 cells analyzed per genotype. For all images, scale bars represent 10 μm and 5 μm for zoomed-in images.

Supplementary Figure S13

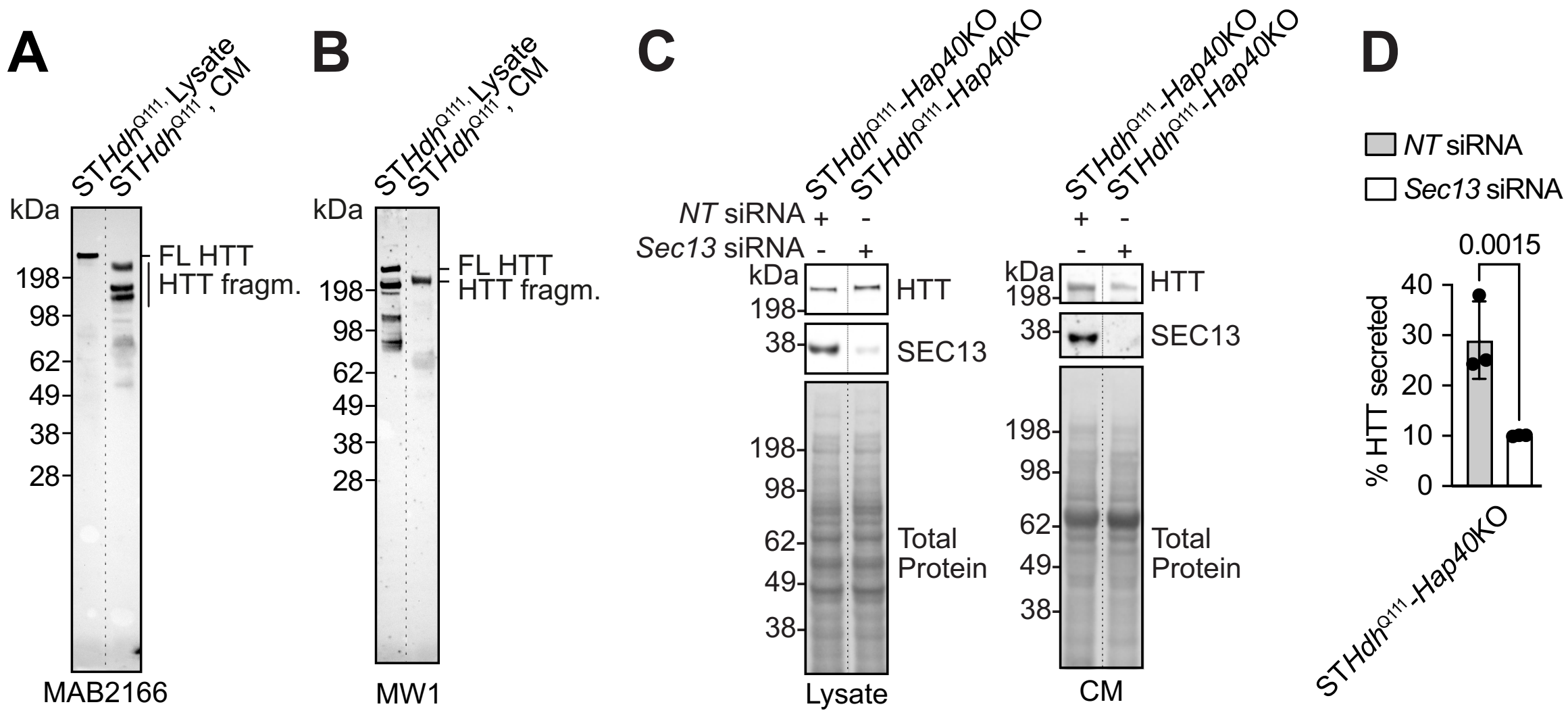

Fig. S13. Characterization of full-length and N-terminal huntingtin fragments and effect of *Sec13* knockdown on HTT secretion.

Representative immunoblots comparing striatal cell lysates and conditioned media (CM) from ST*Hdh*^{Q111} cells probed with **A)** anti-HTT (MAB2166) antibody and **B)** anti-polyQ (MW1) antibody, indicating full-length HTT (FL HTT) and N-terminal HTT fragments (HTT fragm.). **C)** Representative immunoblots of striatal lysates and conditioned media (CM). Cells were treated with siRNA targeting *Sec13* or with a non-targeting siRNA for 72 hours. Membranes were probed with anti-HTT (MAB2166), anti-SEC13 and total protein staining. **D)** Quantification assessing the percentage (%) of HTT secreted into the conditioned media after siRNA treatment, related to **C**. Data are presented as mean ± SD of three biological replicates. Statistical significance was assessed by two-way ANOVA with Tukey's multiple comparisons test.

Supplementary Figure S14

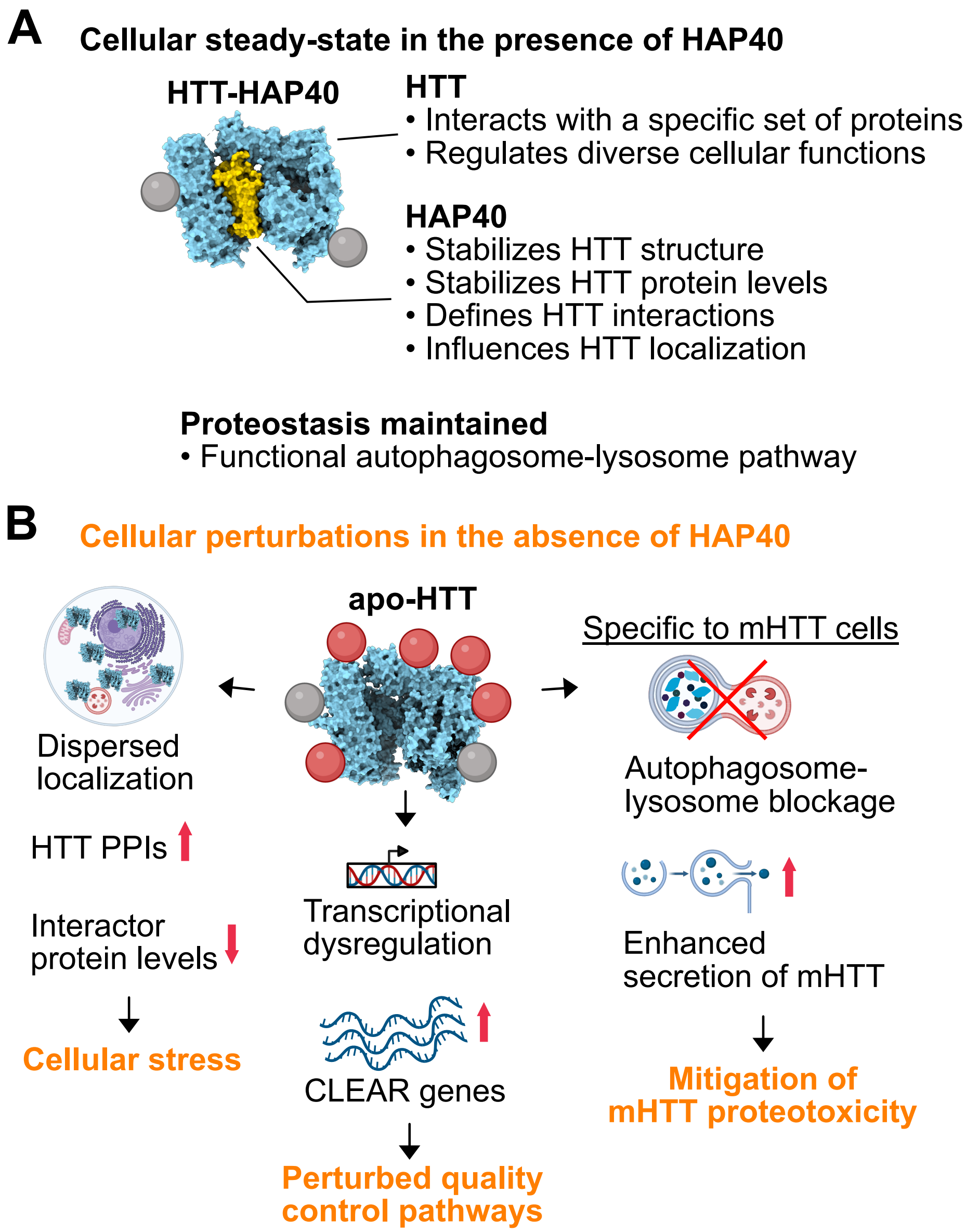

Fig. S14. Study model of HAP40 function
A) The HTT-HAP40 heterodimer governs the stability and functional repertoire of HTT. In the presence of HAP40, HTT adopts a stable conformation, localizes to specific subcellular structures, controls the level of associated proteins, and defines which proteins interact with HTT. A primary outcome of HAP40-mediated regulation of HTT is the preservation of autophagosome-lysosome flux and the control of lysosomal activity. **B)** In contrast, loss of HAP40 alters HTT localization and destabilizes apo-HTT conformation, leading to unregulated protein-protein interactions and altered interactor protein levels leading to cellular stress. Additionally, loss of HAP40 causes transcriptional dysfunction, resulting in the upregulation of CLEAR network genes composed primarily of lysosomal genes, that control the activity of quality-control pathways. In the presence of mHTT, loss of HAP40 leads to impaired autophagosome-lysosome flux, to which cells respond by enhancing secretion to mitigate mHTT proteotoxicity. Created in BioRender. Wanker, E. (2026) https://BioRender.com/smle33q.

Legends for Datasets

Dataset S1 (separate file). iBAQ-based proteomic data from IP-MS experiments identifying HTTQ23-associated proteins in HEK293 WT, HEK293-HAP40KO, and HEK293-HTTKO cells.

Dataset S2 (separate file). Identification of core HTT-HAP40 interactors and apo-HTT-dependent proteins derived from cross-species IP-MS comparisons.

Dataset S3 (separate file). TMT-based total proteome profiles of HEK293 WT, HEK293-HAP40KO, and HEK293-HTTKO cells.

Dataset S4 (separate file). Transcriptome profiles of striatal cell lines.

Dataset S5 (separate file). Differentially expressed transcripts (|log2 fold change| > 0.5) in striatal ST*Hdh*^{Q7} and ST*Hdh*^{Q111} cells upon *Hap*40 knockout.

Dataset S6 (separate file). Total proteome profiles of striatal cell lines.

Dataset S7 (separate file). Comparison of lysosomal RNA and protein level changes in ST*Hdh*^{Q7} and ST*Hdh*^{Q111} cells upon *Hap*40 knockout.

Supplementary References

1. R. Jung, *et al.*, Mutations causing Lopes-Maciel-Rodan syndrome are huntingtin hypomorphs. *Hum Mol Genet* **30**, 135–148 (2021).

2. F. Trettel, Dominant phenotypes produced by the HD mutation in STHdhQ111 striatal cells. *Human Molecular Genetics* **9**, 2799–2809 (2000).

3. J. Schindelin, *et al.*, Fiji: an open-source platform for biological-image analysis. *Nat Methods* **9**, 676–682 (2012).

4. S. Bolte, F. P. Cordelières, A guided tour into subcellular colocalization analysis in light microscopy. *Journal of Microscopy* **224**, 213–232 (2006).

5. E. Scherzinger, *et al.*, Huntingtin-Encoded Polyglutamine Expansions Form Amyloid-like Protein Aggregates In Vitro and In Vivo. *Cell* **90**, 549–558 (1997).

6. V. V. Senichkin, E. A. Prokhorova, B. Zhivotovsky, G. S. Kopeina, Simple and Efficient Protocol for Subcellular Fractionation of Normal and Apoptotic Cells. *Cells* **10**, 852 (2021).

7. A. Agrotis, N. Pengo, J. J. Burden, R. Ketteler, Redundancy of human ATG4 protease isoforms in autophagy and LC3/GABARAP processing revealed in cells. *Autophagy* **15**, 976–997 (2019).

8. P. Trepte, *et al.*, LuTHy: a double-readout bioluminescence-based two-hybrid technology for quantitative mapping of protein–protein interactions in mammalian cells. *Molecular Systems Biology* **14** (2018).

9. P. Trepte, *et al.*, A quantitative mapping approach to identify direct interactions within complexomes. [Preprint] (2021). Available at: https://www.biorxiv.org/content/10.1101/2021.08.25.457734v1 [Accessed 30 September 2025].

10. M. G. Alteen, *et al.*, Delineation of functional subdomains of Huntingtin protein and their interaction with HAP40. *Structure* (2023). https://doi.org/10.1016/j.str.2023.06.002.

11. R. J. Harding, *et al.*, Design and characterization of mutant and wildtype huntingtin proteins produced from a toolkit of scalable eukaryotic expression systems. *Journal of Biological Chemistry* **294**, 6986–7001 (2019).

12. N. C. Hubner, *et al.*, Quantitative proteomics combined with BAC TransgeneOmics reveals in vivo protein interactions. *J Cell Biol* **189**, 739–754 (2010).

13. J. Rappsilber, M. Mann, Y. Ishihama, Protocol for micro-purification, enrichment, pre-fractionation and storage of peptides for proteomics using StageTips. *Nat Protoc* **2**, 1896–1906 (2007).

14. P. Mertins, *et al.*, Reproducible workflow for multiplexed deep-scale proteome and phosphoproteome analysis of tumor tissues by liquid chromatography–mass spectrometry. *Nat Protoc* **13**, 1632–1661 (2018).

15. J. Cox, M. Mann, MaxQuant enables high peptide identification rates, individualized p.p.b.-range mass accuracies and proteome-wide protein quantification. *Nat Biotechnol* **26**, 1367–1372 (2008).

16. M. E. Ritchie, *et al.*, limma powers differential expression analyses for RNA-sequencing and microarray studies. *Nucleic Acids Res* **43**, e47 (2015).

17. V. Demichev, C. B. Messner, S. I. Vernardis, K. S. Lilley, M. Ralser, DIA-NN: neural networks and interference correction enable deep proteome coverage in high throughput. *Nat Methods* **17**, 41–44 (2020).

18. J. Cox, *et al.*, Accurate Proteome-wide Label-free Quantification by Delayed Normalization and Maximal Peptide Ratio Extraction, Termed MaxLFQ. *Mol Cell Proteomics* **13**, 2513–2526 (2014).

19. R. Bruderer, *et al.*, Extending the Limits of Quantitative Proteome Profiling with Data-Independent Acquisition and Application to Acetaminophen-Treated Three-Dimensional Liver Microtissues *[S]. *Molecular & Cellular Proteomics* **14**, 1400–1410 (2015).

20. A. Boeddrich, *et al.*, A proteomics analysis of 5xFAD mouse brain regions reveals the lysosome-associated protein Arl8b as a candidate biomarker for Alzheimer's disease. *Genome Medicine* **15**, 50 (2023).

21. A. Dobin, *et al.*, STAR: ultrafast universal RNA-seq aligner. *Bioinformatics* **29**, 15–21 (2013).

22. Y. Liao, G. K. Smyth, W. Shi, featureCounts: an efficient general purpose program for assigning sequence reads to genomic features. *Bioinformatics* **30**, 923–930 (2014).

23. M. I. Love, W. Huber, S. Anders, Moderated estimation of fold change and dispersion for RNA-seq data with DESeq2. *Genome Biol* **15**, 550 (2014).

24. G. Yu, L.-G. Wang, Y. Han, Q.-Y. He, clusterProfiler: an R package for comparing biological themes among gene clusters. *OMICS* **16**, 284–287 (2012).

25. S. X. Ge, D. Jung, R. Yao, ShinyGO: a graphical gene-set enrichment tool for animals and plants. *Bioinformatics* **36**, 2628–2629 (2020).

26. A. Krämer, J. Green, J. Pollard, S. Tugendreich, Causal analysis approaches in Ingenuity Pathway Analysis. *Bioinformatics* **30**, 523–530 (2014).

27. M. Palmieri, *et al.*, Characterization of the CLEAR network reveals an integrated control of cellular clearance pathways. *Human Molecular Genetics* **20**, 3852–3866 (2011).

28. M. Sardiello, *et al.*, A Gene Network Regulating Lysosomal Biogenesis and Function. *Science* **325**, 473–477 (2009).
